# Craving money? Evidence from the laboratory and the field

**DOI:** 10.1126/sciadv.adi5034

**Published:** 2024-01-12

**Authors:** Elise Payzan-LeNestour, James Doran

**Affiliations:** University of New South Wales Business School, UNSW Sydney, Kensington NSW 2052, Australia.

## Abstract

Continuing to gamble despite harmful consequences has plagued human life in many ways, from loss-chasing in problem gamblers to reckless investing during stock market bubbles. Here, we propose that these anomalies in human behavior can sometimes reflect Pavlovian perturbations on instrumental behavior. To show this, we combined key elements of Pavlovian psychology literature and standard economic theory into a single model. In it, when a gambling cue such as a gaming machine or a financial asset repeatedly delivers a good outcome, the agent may start engaging with the cue even when the expected value is negative. Next, we transported the theoretical framework into an experimental task and found that participants behaved like the agent in our model. Last, we applied the model to the domain of real-world financial trading and discovered an asset-pricing anomaly suggesting that market participants are susceptible to the purported Pavlovian bias.

## Teaser

In gambling contexts, humans may crave monetary rewards in the same way that they crave primary rewards such as food.

## INTRODUCTION

Following a long-standing tradition [dating back to Rescorla and Solomon (*1*)], psychologists believe that human behavior reflects the interaction between two segregated learning systems [for recent evidence, see (*2*)]. The first, called the Pavlovian system, identifies associations between conditioned and unconditioned stimuli (e.g., a light cue predicting reward delivery/a painful shock) and then reacts to predictive cues (e.g., approach the reward cue/avoid the same cue when it is predictive of a shock). In contrast, the so-called “instrumental” or “deliberative” system determines the expected outcome of potential actions and selects actions based on their learned value (termed “utility” in economics).

While in many circumstances Pavlovian reactions to cues facilitate instrumental learning (e.g., if the aforementioned light cue is close to the location of reward delivery, engaging with the cue correlates to getting the reward), they can sometimes impair it and induce the opposite behavior to what is required in a given context—a phenomenon known as “Pavlovian misbehavior” (*3*–*5*). This phenomenon was demonstrated with rats approaching a light predictive of food even when that led to food omission (*6*); chickens that were unable to learn to move away from food to obtain it (*7*); and, more recently, humans who were unable to learn to withhold action to obtain a reward in a Go/NoGo paradigm, which has been broadly used as an assay of Pavlovian misbehavior (*8*–*10*).

Neuroscientific evidence suggests that Pavlovian misbehavior pervades human life in various ways, such as the way cigarette smokers choose to self-administer a nicotine-free cigarette rather than obtain an intravenous injection of nicotine (*11*), or when people crave a particular food that they (their deliberative self) do not expect to enjoy—sometimes referred to as “craving” or “irrational/cue-triggered wanting” (*12*).

Here, we apply the craving concept to the domain of repeated risk-taking [following the lead of (*13*, *14*) among others]. We propose that when a gambling cue such as a gaming machine or a financial asset repeatedly delivers a positive outcome, the Pavlovian value and corollary “wanting for” the cue may become sufficiently large that under certain conditions, the agent may engage with it (i.e., play the machine or invest in the asset) even when the expected value is negative—that is, the instrumental and Pavlovian systems are in conflict. We call this hypothesis “Craving by Design” (henceforth, CbD), in reference to recent research suggesting that recreational gamblers are prone to being “addicted by design” if repeatedly exposed to modern gaming machines (*15*).

The CbD hypothesis provides a unified concept to make sense of seemingly disparate phenomena in human risk-taking, including apparent “thrill of gambling” [obtaining utility from the pleasure of risk-taking regardless of whether this improves current wealth (*16*)], apparent “loss chasing” [the desire to gamble to recoup prior losses (*17*)], and the so-called “machine zone”—where casino gamblers are mesmerized by a gaming machine with an apparent disregard for the outcomes (*15*). Under the CbD hypothesis, all these phenomena reflect Pavlovian misbehavior.

The CbD hypothesis may also explain why financial investors continue making bets that frequently deliver a good outcome yet have a negative value expectation due to the occasional occurrence of large losses (i.e., outcomes are negatively skewed). In the finance sector, recent history shows the pitfall of negative skewness in several high-profile cases. For example, financial derivatives called accumulators—sometimes known as “I kill you later” contracts—caused huge losses for wealthy equity investors in Asia in 2009. Similarly, in the property finance sector, ceaseless investing in collateralized debt obligations fueled by apparent greed led to huge losses in 2008 (*18*)—a behavior described as “picking up pennies in front of a streamroller” (*19*). Or as former corporate executive C. Prince famously said in 2007: “As long as the music is playing, you’ve got to get up and dance”—referring to his continued investment in leveraged buyouts even after it had become apparent that doing so would be harmful.

The CbD hypothesis provides a neurobiological foundation for this type of behavior, namely, the tendency to take actions whose payoff profile combines negative skew and negative expected value—henceforth, the “picking pennies bias.” As such, it should be of interest to economists in view of recent acknowledgment that the picking pennies bias is puzzling from the perspective of the corpus of established knowledge in economics. Some economists have noted that the “usual suspect” to explain the bias—that the agent is unaware of the negative value of gambling (“faulty cognition”)—cannot fully account for the anomaly and purely motivational elements seem to also be at stake (*20*, *21*).

Here, we focus on one such motivational factor, to complement the foregoing “usual suspect” as well as other classical explanations for the bias (more below). We set out to demonstrate, theoretically then empirically, that a perfectly informed agent endowed with standard risk preferences from the lens of “prospect theory”—a widely used model of choice under uncertainty (*22*)—can exhibit the picking pennies bias as a result of Pavlovian misbehavior.

## RESULTS

### Formalization of the CbD hypothesis

#### Base model: Standard prospect theory framework

To show this, we combine key elements of prospect theory and the psychology literature on Pavlovian influences into a single framework. We consider situations where an agent decides between prospects with at most two distinct outcomes. This simplified structure captures a wide range of contexts, including the laboratory experiments run for this study. Let *L* = (*x*, *p*; *y*) denote a lottery that yields outcome *x* with probability *p* and outcome *y* with probability 1 − *p*. We assume that *x* is positive, and *y* is negative (i.e., *L* is “mixed”). The agent is to decide between *L* and a sure outcome of 0. Outcomes are evaluated as gains and losses relative to a fixed reference point, which we assume is 0, in line with previous experimental literature (*23*). Assume the agent perfectly knows the statistics of *L* [so there is no “ambiguity” (*24*)]. In this case, *L* is evaluated as

U(L)=w(p)×v(x)−w(1−p)×v(y)  (1)

where *v*(.) is the so-called “value function” and *w*(.) is the “probability weighting function.” For *v*(.), we use the following functional form [originally proposed by (*25*)], which has been widely applied in economics

$$v\left(x\right)=\left\{ \begin{array}{c} x^{\alpha 1}\;\text{if}\;x\geq 0\\ -\lambda |x{|}^{\alpha 2}\;\text{if}\;x<0 \end{array}\right.$$

where λ is the loss aversion parameter (λ > 1 means that the agent is loss averse), α_1_ governs the degree of risk aversion for gains (0 < α_1_ < 1 means that the agent is risk averse for gains; α_1_ = 1 means that the agent is risk neutral for gains), and α_2_ governs the degree of risk seeking for losses (0 < α_2_ < 1 means that the agent is risk seeking for losses; α_2_ = 1 means that the agent is risk neutral for losses). Unless specified otherwise, we take α_1_ = α_2_ = α for simplicity and to avoid scaling issues (*26*). All the conclusions reported in this study apply to either specification. A popular functional form for *w*(.) is *w*(*p*) = exp. {−(−ln*p*)^α3^}, with 0 *<* α_3_ ≤ 1 (*27*). Here, we assume no probability weighting (α_3_ = 1) unless stated otherwise, chiefly to simplify the exposition and in line with recent work suggesting that this feature often plays only a minor role (*28*, *29*).

To model decision, we use the popular logit/softmax rule (chiefly for simplicity and without loss of generality). The agent chooses *L* with probability

$$P\left(L\right)=\frac{e^{\beta U\left(L\right)}}{1+e^{\beta U\left(L\right)}}$$ (2)

The parameter β governs the degree of choice randomness or “trembling” (if β = 0, choice is completely random; if β → ∞, choice is deterministic).

To clearly show that the picking pennies bias can emerge within the standard framework used in economics, we shall assume here that our perfectly informed agent features standard risk preferences [say λ ~ 2 and α ~ 0.8, which is in the range of expected values for these parameters, looking across past empirical studies aimed at estimating Prospect Theory parameters (*23*)] and no trembling (β → ∞). We further assume that these characteristics are stable (they do not change over time). It is obvious that in this framework, without further assumptions, the agent always rejects *L* if the expected value of *L* is negative [*EV*(*L*) = *p x* + (1 − *p*) *y <* 0].

#### CbD model

Further assume that the above decision is recurrent, occurring at discrete intervals or “trials” *1*, *2…*, *T*. After each decision, the agent observes the outcome delivered by *L*, and she is to decide again whether to take the same lottery *L.* Let *t* denote the current trial and 1*_x_*(*k*) denote the indicator function taking value 1 if *L* delivers *x* at trial *k* and 0 otherwise. We model craving, denoted by *DA*(*t*), as the following function of the Pavlovian cumulative reward value *V*(*t*)

$$\mathrm{DA}(t)=\text{max}\{f[V(t)],0\},\;\text{where}\;\{\begin{array}{c} f:u\to \frac{1}{1+e^{-\kappa_{1}u}}-\kappa_{2}\\ V\left(t\right)=\sum_{k=1}^{t}\theta^{t-\kappa }1_{x}\left(\kappa \right) \end{array}$$ (3)

We set 0.1 ≤ κ_1_ ≤ 1, 0.5 ≤ κ_2_ ≤ 1, and 0.9 ≤ θ ≤ 1 (see below for the interpretation of these parameters). We chose the logistic form for the craving function chiefly for tractability reasons; the specific form is not pivotal so long as the function is bounded (to reflect neuron saturation, a basic aspect of neuronal dynamics).

**Fig. 1.**
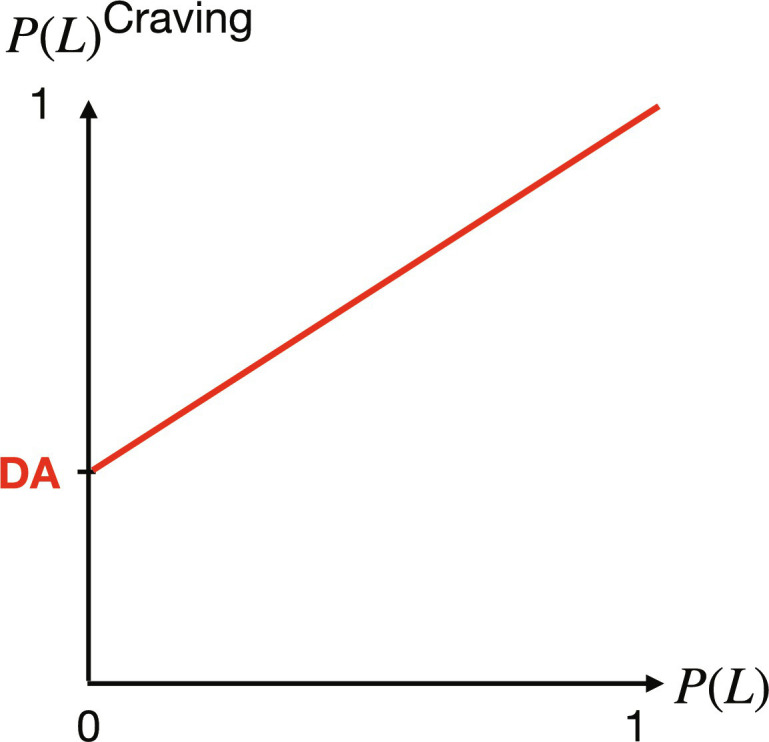
Gambling bias The craving factor *DA*(*t*) defined in Eq. 3 takes value “DA” in this example. *X* axis: *P*(*L*), the probability to gamble computed by the instrumental system. *Y* axis: *P*(*L*)^craving^, the distorted probability to gamble (Eq. 4). For values of *P*(*L*) < 1, craving increases the probability that the agent chooses to gamble.

Following Rutledge *et al.* (*13*, *14*), we modeled the Pavlovian influence on instrumental behavior as a distortion of the instrumental action probability. At trial *t* + 1, the probability to choose *L* is

$${P(L)}^{\text{craving}}(t+1)=\mathrm{DA}(t)+[1-\mathrm{DA}(t)]P(L)\;$$ (4)

where *P*(*L*) is the gambling probability that prevails in the absence of Pavlovian influence (see Eq. 2). Our motivation for modeling the Pavlovian influence as a distortion of action probability rather than action value [as is commonly done in the literature (*5*–*9*)] is that the gambling bias induced by the Pavlovian influence is directly apparent under this specification of the model (see Fig. 1). However, it should be noted that modeling the Pavlovian influence as a modulation of the instrumental value instead is identical within the current framework (see Supplementary Text for details).

### Model interpretation and direct extensions

#### Pavlovian value and craving power of a gambling cue

The core element of the CbD model is the Pavlovian cumulative reward value *V*(*t*) in Eq. 3, which is equivalent to the cached Pavlovian learned value acquired through “temporal difference” (TD) reinforcement learning as proposed in the literature [e.g., (*5*, *30*, *31*)]. The temporal discounting parameter θ is inversely related to the learning rate in the standard TD learning framework (increased “forgetting” in the agent implies a higher learning rate).

Here, we postulate that Pavlovian learning works equally for directly experienced and observational outcomes, both to reflect recent findings [see (*32*) in particular] and to discipline the model—allowing for different weights for the two types of outcomes would not qualitatively change our main model predictions, but it would make the model more complicated (adding at least one free parameter). In either specification, the key idea of the model is that craving, which is computationally captured by the incentive value of the gambling cue [*DA*(*t*)], increases with the Pavlovian value of the cue [*V*(*t*)], which itself reflects the strength of the association between *L* and the monetary reward *x* (reward probability *p*).

Previous work suggests that the extent to which a Pavlovian cue can trigger craving increases not only with reward probability but also with reward size (*x*) (*33*–*35*). The CbD model can readily reflect this by amending the definition of *V*(*t*) as follows. Define *V*(1) ∝ 1*_x_*(1)*x*, and then, for *t >* 1

$$V\left(t\right)\propto \theta^{t-1}V\left(1\right)+\sum_{k=2}^{t}\theta^{t-k}1_{x}\left(\kappa \right)\times \left(\frac{x}{\omega +x+\sum_{u=1}^{k-1}\theta^{k-u}1_{x}\left(u\right)x}\right)\;$$

where ω is a positive constant reflecting saturation in neuronal responses. In this expression for the Pavlovian learned value *V*(*t*), which was inspired by (*36*), reward value is normalized by the recent reward history $\sum_{u=1}^{k-1}\theta^{k-u}1_{x}\left(u\right)x$ , to account for the well-evidenced phenomenon of “divisive normalization” (*37*). The basic idea is that the perception of a stimulus depends not on its absolute intensity but rather on its intensity relative to what the agent expects (which is assumed to depend on the recent outcome history).

#### The “gating” κ parameters can explain variations in craving propensity within and across individuals

In the CbD model, the central parameter *DA*(*t*), which measures craving for the gambling cue *L* [so-called “cue-triggered wanting” (*38*)], is a function of the Pavlovian cumulative reward value but not its equivalent—contrary to the theory in early literature on Pavlovian influences (*30*). Rather, we allow for the representation of the Pavlovian value to be modulated by the κ parameters, which capture the motivational state of the agent.

**Fig. 2.**
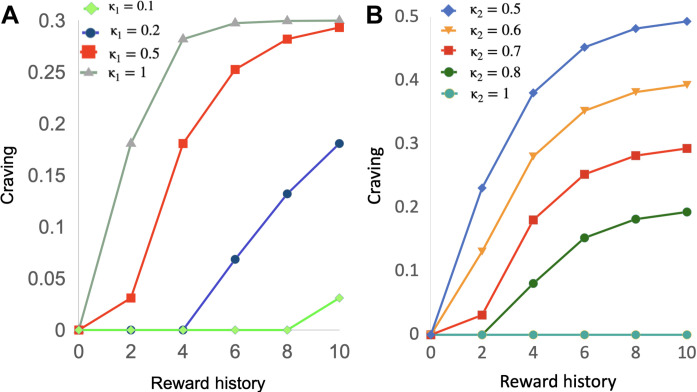
The strength of craving depends on the agent’s motivational state. The graphs show how the strength of craving (*y* axis), which is a function of reward history (*x* axis), is modulated by the κ parameters. (**A**) Impact of varying the value of the parameter κ_1_ on the craving factor for the case θ = 0.9, κ_2_ = 0.7. (**B**) Impact of varying κ_2_ for the case θ = 0.9, κ_1_ = 0.5.

This feature of the model was inspired by the work of Zhang *et al.* (*31*) [among several others, see (*9*) for example]. The parameter κ_1_ corresponds to the “gating parameter” κ in their model [while the second gating parameter κ_2_ can be interpreted as being related to response vigor” (*39*)]. Higher (resp. lower) values for κ_1_ (resp. κ_2_) increase craving (Fig. 2).

Allowing for a modulation of the representation of the Pavlovian value as a function of the agent’s current motivational state is believed to be important in order to account for instant shifts in Pavlovian responses that follow a physiological change without any change in the learned Pavlovian value, e.g., a sudden state of food deprivation increasing wanting for a food cue independently of the learned value of the cue (*40*). Recent research further suggests that in the present framework, one key determinant of the κ parameters is the perception of “controllability” in the agent, that is, the extent to which the agent feels that her actions affect the outcomes (lower controllability would increase κ_1_) (*10*, *41*). Other determinants may be the agent’s current “appetite” for monetary rewards (κ_1_ could increase with factors such as deprivation and perhaps the degree of personal intrinsic interest in money), as well as genetic and epigenetic factors thought to explain inter-individual differences in the susceptibility to reward sensitization (*42*, *43*).

### Model predictions

#### “House money effect”

The CbD model predicts increased risk-taking after a series of good outcomes, as seen in the celebrated “house money effect” (*44*). In our framework, the house money effect occurs because craving [*DA*(*t*)] increases with the frequency of good outcomes observed in the past (see the “Pavlovian value and craving power of a gambling cue” section). This differs from the conventional explanation in economics, which singles out path-dependent reference points (*45*, *46*). In the CbD model, the agent behaves as if her reference point has changed, but we should recall that it does not change by assumption (it is fixed to 0). Thus, the CbD model can predict the house money effect in settings where the reference point cannot realistically vary within the sample period [for recent work on the speed of adjustment of the reference point, see (*47*)].

#### Settings where the Pavlovian influence is synergistic with the instrumental action

As highlighted in Introduction, in many cases, the Pavlovian influence is congruent with the goal of the instrumental system. The CbD model captures this. We should recall indeed that craving increases the probability that the agent will choose to gamble (Fig. 1). Therefore, whenever the gamble has positive instrumental value [*U*(*L*) > 0] and the agent’s behavior exhibits randomness [*P*(*L*) < 1], the gambling bias shown in Fig. 1 will help the instrumental system implement the optimal course of action (gambling) by boosting the probability of choosing that action. The more stochastic this behavior is (the smaller β), the higher the synergy between the two systems (N.B., in the case of the ideal—noise free—chooser, the Pavlovian boost is redundant).

#### Case where the Pavlovian and instrumental systems are in conflict: Picking pennies bias

The opposite scenario occurs when the instrumental and Pavlovian systems are clearly in conflict, which is our main focus in this study. This will happen when 0.5 < *p* < 1 and *EV*(*L*) < 0. In this context, the instrumental value *U*(*L*) is unambiguously negative. {Note that the implication [*EV*(*L*) < 0 ⟹ *U*(*L*) < 0] holds under the assumption that the agent is perfectly aware of the statistics underlying *L*, and she is risk/loss averse. We relax both assumptions below.} So normally (in the absence of craving), the agent systematically rejects negatively skewed lotteries of this kind, i.e., *P*(*L*) = 0 (assuming no randomness in choice here for the sake of the argument). However, because the reward probability of the gamble is large (*p >* 0.5), the Pavlovian value *V*(*t*) and hence craving [*DA*(*t*)] can become sufficiently large to lead the agent astray. This is illustrated in Fig. 1 [see the point *P*(*L*) = 0 on the *x* axis]: Because of craving, the agent chooses *L* with probability *DA* in the example shown on the graph. This means that if, for example, *DA* = 0.1, the agent chooses *L* 10% of the time; if *DA* = 0.2, the agent chooses *L* 20% of the time. Such values for the craving factor seem plausible. To see this, assume the agent just observed ten $2 outcomes in a row with the lottery *L =* (2,0.8; −40). This scenario is highly likely to occur with such a lottery given the high reward probability (0.8). Assume for instance that θ = 0.9, κ_1_ = 0.5, and κ_2_ = 0.7 (Fig. 2, red curve—these parameter values correspond to the average fitted values across the task participants in our experiments, see next). In this case, the craving factor *DA* is negligible for the first two trials; it is around 0.15 at trial 4 and plateaus around 0.25 from trial 6.

#### Risk/loss tolerance versus craving: Dynamics of the bias

A key prediction of the model, which the previous example illustrates, is that the picking pennies bias only starts after Pavlovian conditioning has taken place. Initially, the craving factor *DA* is negligible, so there is no gambling bias; the bias only appears after the Pavlovian value *V*(*t*) has had time to build.

This pattern for the dynamics of the bias is a hallmark of craving inasmuch as it cannot be generated by allowing for risk-seeking preferences in the base model (the model devoid of the craving component). To appreciate this, relax the assumption α_1_ = α_2_ and allow for risk-seeking preferences both for gains and losses (i.e., α_1_ *>* 1 and α_2_ *<* 1). Further assume no trembling (for simplicity and without loss of generality). From Eq. 1, it is clear that if α_1_ is above the threshold $\frac{\text{ln}\left(\lambda \frac{1-p}{p}{|y|}^{\alpha_{2}}\right)}{\text{ln}x}$ , the agent always chooses *L* [i.e., *U*(*L*) *>* 0], and the agent always rejects *L* otherwise. Similarly, assuming “loss tolerance” (*48*), i.e., λ *<* 1, one infers from Eq. 1 that the agent always takes *L* if λ is below a certain threshold, and the agent always rejects *L* otherwise. We can therefore conclude that the gambling bias generated by risk/loss tolerance is such that either the agent always gambles (in the case of a noise-free chooser) or gambles with fixed (time invariant) probability (in the case of a stochastic chooser). In either case, this form of bias does not conform to the bias generated by craving in the CbD model.

#### Effect of training on the bias

Recent research suggests that the picking pennies bias predicted by the CbD model shall decrease with training, in relation to the foregoing “controllability” factor (*10*, *41*). That is, the Pavlovian influence will be strongest when the agent has little experience with the decision task at hand (making the action values computed by the instrumental system unreliable) and progressively weaker as the agent gets used to the task.

This feature of the Pavlovian bias makes it distinct from habit-related biases. In psychology, “habit” refers to the “autopilot” behavioral mode that proceeds once instrumental learning has taken place (*49*–*51*). In short, when the instrumental system has learned that action *A* is optimal in context *s* after many repetitions, behavior may transition to the habit mode: identifying *s* automatically triggers *A*, which conveniently obviates the need to keep track of the learned instrumental action values and is therefore adaptive when these values are reliable. Take for example the current framework in which the agent is to repeatedly decide whether to gamble. Deliberation would typically take place at the beginning of the task as described by the base model above (assuming no craving here): The agent computes *U*(*L*), and because it is negative in the context of interest here [0.5 < *p* < 1 and *EV*(*L*) < 0], the agent decides to reject *L*. If the task is repeated for many trials, at some point behavior will likely become habitual: The agent systematically rejects *L* without having to think about it, which saves mental resources (*51*).

Habit-formed behavior can, however, be harmful when the learned instrumental values are inaccurate. This will occur whenever the instrumental system uses a model-free reinforcement learning approach to learn action values (*52*) and past outcome history is not representative of the outcome distribution. The current context of interest [0.5 < *p* < 1 and *EV*(*L*) < 0] demonstrates this clearly. To see this, relax the assumption that the agent computes the instrumental value of gambling based on the statistics of *L* and assume instead that the agent uses a model-free approach such as the standard “Q-learning” method or the like (*53*, *54*). We denote by *Q*(*L*) the model-free instrumental value of gambling. Because of the frequent streaks of good outcomes delivered by the negatively skewed *L*, the instrumental value of gambling is typically positive [*Q*(*L*) > 0], and hence, the agent is likely to take the lottery [*P*(*L*) >> 0] until a bad outcome is eventually observed, depressing *Q*(*L*) [see (*54*) for an illustration of this behavior in simulations]. However, if the model-free agent has already switched to the habit mode when the bad outcome occurs, he will be insensitive to the fact that *Q*(*L*) is negative (*49*, *50*), and the previously reinforced action (gambling) will tend to continue.

The conclusion, therefore, is that habit in a model-free instrumental chooser can generate a gambling bias. However, by nature, habit needs time to develop, implying that the habit-related bias is not observed at the beginning of the task, whereas the gambling bias generated by craving is expected to be strongest at the beginning—when the uncontrollability feeling is purportedly at its highest (*10*).

#### Overcoming the bias: Self-control and nudging

Recent work provides evidence for diminished Pavlovian misbehavior in individuals who can actively overcome the Pavlovian influence through prefrontal control mechanisms (*9*). Together with these recent insights, the CbD hypothesis predicts that the picking pennies bias generated by craving should be reduced in individuals endowed with greater self-control. Inhibiting the Pavlovian influence could lead to a decreased value for the κ_1_ parameter in those individuals. Whatever the individual’s cognitive control capabilities, the bias generated by craving vanishes in contexts where the agent makes her decision before any reward history is observed (i.e., before Pavlovian conditioning has taken place). For example, if the agent is offered a “commitment device” (*55*) whereby she can commit to a course of action before any outcome is observed, the likelihood of observing the bias shall decrease relative to the baseline case with no commitment device.

### Experimental design

#### Gambling paradigm

Our main motivation for developing the CbD model was to show that in theory, a perfectly informed and “standard” (in terms of risk attitude) agent can nonetheless exhibit the picking pennies bias due to craving. Next, we set out to test this idea and study the importance of the craving versus faulty cognition factors in contexts where people are not informed about outcome statistics (as is often the case in reality). To that goal, we transported the abstract framework of the CbD model into an experimental task and asked undergraduate students to perform one run of this task at the University of New South Wales (UNSW) Business Experimental Research Laboratory.

We reasoned that the task should be both engaging (to address a common criticism that experimental designs relying on choices between lotteries fail to engage task participants) and “neutral” (*56*): not evocative in any sense and, at the same time, not abstract. For example, we refrained from framing the task as a financial decision-making problem both to avoid influencing participant behavior and to ensure that everyone can grasp the principle of the task, including financially illiterate participants [which may apply to a substantial portion of the sample in view of documented financial illiteracy among the general population (*57*)].

**Fig. 3.**
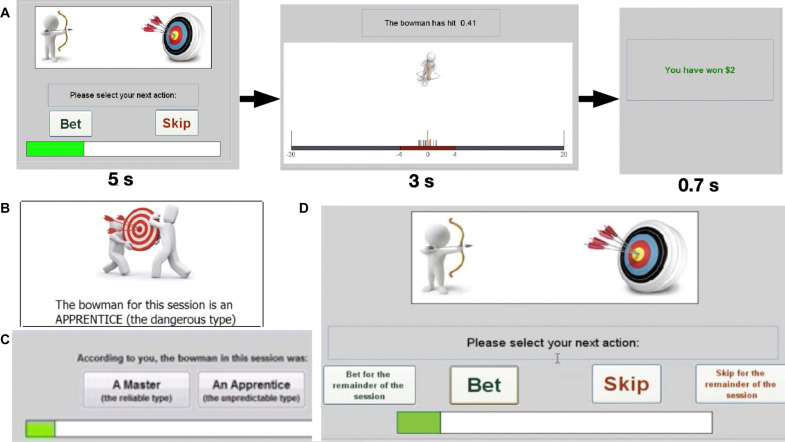
Experimental design. (**A**) The task consists of fifteen 20-trial blocks or “sessions,” each with a different bowman who is either a master (in which case the expected value of betting that the next shot will fall within 4 m away from the target is positive) or an apprentice (in which case betting has negative expected value). Each trial of the task starts with a choice phase in which the participant is to decide whether to bet that the next shot will fall within the winning range (5 s, self-paced), followed by a visual observation of the result (the history of the previous shots is also shown) for 3 s, and explicit feedback regarding the outcome obtained at the trial (0.7 s). In experiment 1, participants were not informed of the nature of the bowman in each session; however, it could be deduced by observing the shot realized on each trial. (**B**) In experiment 2, participants were made aware of the nature of the bowman before each session began. Apart from that feature, the task was the same as the one used in experiment 1. (**C**) The task used in experiment 3 is an augmented version of the task used in experiment 1 in which participants were asked to report their belief about the bowman type at the end of each session. (**D**) In the task used in experiment 4, in each trial, the participant can exercise the option to bet/skip for all the remaining trials of the session by clicking on the “bet for the remainder of the session”/“skip for the remainder of the session” buttons situated next to the regular bet/skip buttons. Apart from that feature, the task is the same as the one used in experiment 1. For a demo of the task used in each experiment, see http://bowmangame.weebly.com/.

Taking these requirements into account, we arrived at the following design. The experimental task resembles a video game in which, at each trial *t*, a bowman is shooting at a target on a wall. The wall is represented by a line; the target corresponds to the zero mark on that line, and the shot realized, denoted by *X_t_*, can be anywhere on the line. The participant must decide whether to bet that the coming shot will fall up to 4 m away from the target on both sides (i.e., *X_t_* ∈ [−4;4]). A winning bet yields $2; a losing bet (*X_t_* ∉ [−4;4]) results in a loss of $40. The alternative to betting is to “skip,” which guarantees $0. Immediately after deciding whether to bet or skip, the participant sees the realized shot, is informed of the outcome, then proceeds to the next trial (Fig. 3A).

The participant plays 15 independent sessions comprising 20 trials each. Participant payoff consists of her final net accumulated outcomes from all sessions, plus/minus a starting account balance, the value of which is revealed to the participant upon completion of the task. See Supplementary Text for a detailed description of this payoff rule—recently developed as an incentive device for participants in demanding cognitive tasks (*54*, *58*). The rule achieves this by addressing the issue of “diluted incentives” (*59*), averting wealth effects, and generating a bimodal payoff distribution—high performers typically earn more than $100, while mediocre players leave the laboratory with either very little (the $5 show-up reward) or even a debt (in one of our experimental treatments, more next). If the participant does not make a decision within the allotted time [5 s; elapsed time is indicated by a timer, see Fig. 3A], she loses $1 and does not see the hit realized. [Across all the experimental sessions run for the study, there were very few missed trials and participants responded well before the allotted time (table S1).]

We designed the task instructions to ensure that everyone could grasp the stochastic structure of the task, even those unfamiliar with basic statistical concepts. The participant is told that each session has a different bowman, either a “master” or an “apprentice,” with equal chance of success. Animations or “distribution builders” showing 300 successive sample shots from master and apprentice bowmen are displayed onscreen, allowing the participant to grasp the shot distributions from the different kinds of bowmen through repeated sampling of the distributions.

From these animations, it is apparent that the shots from a master bowman are normally distributed around the target, i.e., mean is 0, and that for each possible value of the SD of the shots—which, the participant is informed, is drawn from a uniform distribution [0.1,2] for each master bowman—the probability of a winning bet is close to 1. Therefore, the expected value of betting is positive when the bowman is a master. (The specific calculation can be found in Supplementary Text; N.B., this is not supposed to be performed by the participant; more next.)

It is equally apparent from the animations that the expected value of betting is negative when the bowman is an apprentice. To be more specific, the shots from an apprentice bowman are Cauchy distributed with a dispersion parameter fixed to 1, so the probability of a winning bet when the bowman is an apprentice is around 0.8. The exact value and how to compute it is provided in Supplementary Text.

The participant is not supposed to perform these explicit computations, and it is not assumed that she knows the exact value during the task. The main conclusions in the analyses below are the same if we assume that their probability estimates are imprecise. It is only assumed, in light of recent work (*60*–*62*), that the animations allow the participant to intuitively grasp that the expected value of betting is positive with a master bowman and negative with an apprentice, and that the information can “sink in” (*63*) when presented in this format. This methodology and evidence that it worked is expounded in Supplementary Text.

Where the participant is familiar with statistical concepts, the stochastic structure of the task is explained more comprehensively through explicit statistics, both in the online instructions and in frequently asked questions (FAQs) distributed just before the participant performs the task (for reproducibility, more details on the procedures are provided in Supplementary Text).

#### Applying the model to the task

##### (i) Perfect information

In the “no ambiguity” version of the task, before each session begins, the participant is told the nature of the bowman. Therefore, like the agent in our model, she knows what to expect if she chooses *L*. (In the context of the experimental task, choosing *L* means engaging with the bowman.) Simply noting that *EV*(*L*) > 0 in sessions with a master bowman, one can directly apply the result shown in the “Settings where the Pavlovian influence is synergistic with the instrumental action” section: In sessions with a master, the behavior of a participant who is under the Pavlovian influence will be closer to the rational benchmark (which is to bet) than the behavior of a participant who is not.

Likewise, noting that *L =* (2,0.8; −40) in sessions with an apprentice, one can apply all the results shown in the “Case where the Pavlovian and instrumental systems are in conflict: Picking pennies bias” section: In sessions with an apprentice, the instrumental value of gambling is negative, yet a participant susceptible to craving bets with positive probability after observing a series of shots within the winning range. What this means at the session level is that a “standard” (i.e., risk/loss averse) agent exhibits time inconsistency: She typically skips in the early trials of the session but then tends to bet later during the session. If devoid of the craving component, that same agent would never bet—or seldom bet, if we allow for some degree of choice stochasticity (see the “Risk/loss tolerance versus craving: Dynamics of the bias” section).

##### (ii) What if the task participant is not instructed about the nature of the bowman?

In this more “realistic” version of the task, the nature of the bowman is not revealed before the session begins, but it can be inferred through observing the outcome delivered by the bowman on each trial. This allows the participant to compute the probability of a winning bet. To be more specific, assume Bayesian beliefs and denote the shot history available until (included) trial *t* − 1 by $\underline{X_{t-1}}$ = (*X*_1_, *X*_2_,···, *X*_*t*−1_). The participant’s estimated probability of a winning bet at trial *t* is

$$p(t)=P(M_{1}|\underline{X_{t-1}})\;\times \;p_{g}+P(M_{2}|\underline{X_{t-1}})\;\times \;p_{c}$$ (5)

where *P*(*M*_1_|$\underline{X_{t-1}}$ ) [resp. *P*(*M*_2_|$\underline{X_{t-1}}$ )] denotes the posterior probability that the bowman is a master (resp. an apprentice), *p_g_* is the probability of a winning bet when the bowman is a master, and *p_c_* is the probability of a winning bet when the bowman is an apprentice. Both posterior probabilities are small at the beginning of each session (note the prior value is 1/2 by design) and the value of *P*(*M*_1_|$\underline{X_{t-1}}$ ) typically grows in a session with a master (for computational details, see Supplementary Text). Recall that by design *p_g_* ≈ 1 and *p_c_* ≈ 0.8. So *p*(*t*) is well approximated by

$$p(t)\approx P(M_{1}|\underline{X_{t-1}})\;\times \;1+P(M_{2}|\underline{X_{t-1}})\;\times \;0.8\;$$ (6)

We can then apply the base model, replacing *p* with *p*(*t*) in the specification given by Eq. 1. Assuming no trembling here for the sake of the argument, one infers that a participant devoid of the craving component chooses to bet at trial *t* if *p*(*t*) is above the following probability threshold $\bar{p}$

$$\bar{p}=\frac{\lambda }{{\left(\frac{2}{40}\right)}^{\alpha }+\lambda }$$ (7)

As one would expect, the threshold increases with the participant’s degree of loss aversion (λ). For example, for the standard agent featuring λ = 2 and α = 0.8, $\bar{p}$ is about 0.95. If λ = 1 instead, i.e., the agent is “loss neutral,” $\bar{p}$ decreases to 0.91. If λ *<* 1 (i.e., the agent is loss tolerant), $\bar{p}$ is below 0.91.

In the first trials of each session, it is unknown whether the bowman is a master [*P*(*M*_1_|$\underline{X_{t-1}}$ ) small]. Therefore, a participant with standard risk preferences systematically skips [*p*(*t*) *<* $\bar{p}$ , so *U*(*L*) < 0]. The agent starts betting [*p*(*t*) *>*  $\bar{p}$ ] only when/if the evidence that the bowman is a master becomes large enough. This often happens in sessions with a master after a few trials, and sometimes in sessions with an apprentice too, when the past outcomes “look” Gaussian and the participant is deluded that the bowman is a master [*P*(*M*_1_|$\underline{X_{t-1}}$ ) large]. See (*54*) for an illustration of this behavior in simulations.

However, whatever the past outcomes, the participant shall systematically skip after observing a “black swan,” here defined as a shot falling more than 7 m away from the target. Tail events of this kind never happen with a master, so seeing one shall make it clear to the participant that the bowman is an apprentice: *P*(*M*_2_|$\underline{X_{t-1}}$) ≈ 1, so *p*(*t*) ≈ 0.8 (see Eq. 6). We are thus back to the previous configuration of perfect information with *L =* (2,0.8; −40), and the results shown in the “Case where the Pavlovian and instrumental systems are in conflict: Picking pennies bias” section apply here. That is, under no trembling, a “normal” participant never bets. In contrast, a participant susceptible to craving exhibits the picking pennies bias—betting with positive probability despite knowing that the bowman is an apprentice.

Note that the second behavioral signature of the Pavlovian influence discussed for the “no ambiguity” version of the task also applies here: In the sessions with a master, a participant who is susceptible to craving starts betting earlier than a participant who is not, which illustrates the synergistic interaction of Pavlovian and instrumental decision-making in that case.

#### Experimental strategy

To summarize, in the instantiation of the theoretical framework in the laboratory, the picking pennies bias manifests when a task participant bets with positive probability despite knowing that the bowman is an apprentice. That means either betting with positive probability in a session with an apprentice despite being informed of the bowman’s type (in the no ambiguity version of the task) or betting with positive probability after observing a shot falling more than 7 m away from the target (in the more realistic task setting).

Comparing the prevalence of the bias in the two versions of the task is crucial to test the CbD hypothesis against the faulty cognition account for the bias. If we were to observe the bias in the realistic version of the task alone, we could not fully rule out the possibility that the participants who exhibited the bias (henceforth, the “penny-pickers”) were unable to infer that the bowman was an apprentice upon observing a black swan. Although we can control for this possibility (more next), it cannot yield insights into the relative importance of the faulty cognition and craving factors in the field unless we study behavior in both versions of the task and ascertain the participants’ beliefs regarding the bowman type.

This motivated us to compare the prevalence rate of the bias in experiment 1 (*N* = 124), where the task participants performed one run of the realistic version of the task, and experiment 2 (*N* = 44; same cohort as in experiment 1), where the task participants performed the no ambiguity version (Fig. 3B). We also ran experiment 3 (*N* = 52; same cohort as in the previous experiments), which augmented the design of experiment 1 by asking participants at the end of each session whether the bowman was a master or apprentice (Fig. 3C). A correct answer yielded $10, and an incorrect answer resulted in a loss of $10. This rule was emphasized in the task instructions to ensure that participants were provided with the right incentives. We compared the accuracy of the penny-pickers to the accuracy of the other participants.

Besides controlling for the possibility that some of the participants may have been unable to infer that the bowman was an apprentice after observing a black swan in the learning version of the task, we aimed to control for other potential explanations for the picking pennies bias that are unrelated to craving and hence would “compete” with the CbD hypothesis if we were to observe the bias in participants. We considered seven categories of competing explanations, four of which can be tested against the CbD hypothesis through probing the dynamics of the bias in the laboratory (and assessing whether they “match” the model predictions), while the remaining two required us to run two separate experimental treatments.

#### The bias could reflect overestimation of the probability of a winning bet in the sessions with an apprentice

As stated earlier, we designed the instructions to ensure that participants could easily understand the probability of a winning bet, and also introduced strong incentives encouraging participants to study the information provided. Still, we cannot fully rule out the possibility that penny-pickers overestimate *p_c_*, the probability of a winning bet in the sessions with an apprentice (due perhaps to the participants’ limited attention span), whereupon they mistakenly think that betting has a positive expected value. If so, they should bet consistently, including in the first trials of the sessions with an apprentice, in contrast to the picking pennies bias generated by craving in the CbD model.

The bias could also reflect the “gambler’s fallacy”: In both versions of the task, immediately after seeing a black swan, penny-pickers may mistakenly think that “the disaster event has happened already, now a near miss is due so I shall bet.” If so, the occurrence of the bias should be restricted to the trials immediately following the occurrence of black swans during the task, opposite to that in the CbD model. In addition, a participant susceptible to the gambler’s fallacy will skip after a prolonged series of near misses (mistakenly thinking that a tail event is “due”)—again a pattern at odds with the CbD model.

#### The bias could reflect “probability matching”

In probability matching—a widespread behavioral tendency thought to be a product of evolution (*21*), the frequency of choice of a given action matches the probability of success with that action, i.e., in the current task setting, betting 80% of the time when the winning bet probability (the objective probability in the no ambiguity version of the task, the estimated probability in the learning variant) is 0.8. A participant that follows such a decision rule starts betting early within a session, as can be shown in simulations (fig. S1), at odds with what the CbD model predicts.

#### The bias could reflect risk seeking/loss tolerance

As shown above, the picking pennies bias generated by high levels of risk/loss tolerance and the one generated by craving are of different nature. At the session level, the former manifests itself from the beginning and it is time invariant, whereas the latter is only observed later during the session.

#### The bias could reflect choice randomness and limited attention/memory

Therefore, to summarize, probing the dynamics of the bias exhibited by the task participants at the session level is a litmus test of the foregoing explanations against the CbD hypothesis. The bias could also reflect choice randomness (“trembling”) rather than craving. To correct for the occurrence of random mistakes, our count of the penny picking instances at the session level only included instances in which the participant bet at least twice in a session with an apprentice, for the no ambiguity version of the task/at least twice after observing a black swan in a session, for the learning version of the task. (Note: All the results reported below are robust to including all instances in the count.)

In the learning version of the task, the bias could also come from the penny-pickers’ overlooking/forgetting the previous occurrence of a black swan because of limited attention/memory. This possibility motivated us to compute the fraction of penny-pickers using only the subsample of black swan events associated with a $40 loss. We reasoned that those should be quite salient (given the contrast between loss and gain magnitudes in the current task—$40 versus $2) and hence memorable. Therefore, using such a data filter should help sift out instances of the bias that are potentially generated by limited attention/memory. We reasoned that if the percentage of penny-pickers remained qualitatively unchanged after applying this filter, this would rule out participant oversight/forgetting as being among the key factors underlying the picking pennies bias here.

#### The bias could reflect habit in model-free instrumental learners and/or participant fatigue

As explained above, the CbD model assumes that the instrumental system uses a model-based approach, but if the participant was to use a model-free approach instead, then a habit-related gambling bias could in principle emerge (see the “Effect of training on the bias” section). Habit is (arguably) unlikely to occur in the learning version of the task given that for the participant each session starts “anew” with a new bowman and action values must be relearned from scratch. However, habit may well develop in the no ambiguity version of the task, which is quite repetitive. That is, in the late sessions of the game, the participant could exhibit a tendency to automatically decide what to do with each kind of bowman. If the instrumental value of betting in a session with an apprentice [*Q*(apprentice, bet)] happens to be positive when behavior becomes habitual, the model-free participant tends to bet irrespective of the outcomes.

This scenario is perhaps not the most likely given recent evidence that task participants are more likely to use a model-based approach in the current experimental paradigm (*54*), perhaps not surprisingly. [Note: This could arise from the fact that the task instructions spell out the nature of the stochastic structure underlying outcomes, thereby nudging participants into approaching the task in a model-based (probabilistic) manner; moreover, the model-based approach markedly outperforms model-free “competitors” in this setting (*52*).] We still examine this hypothesis along with the possibility that at some point during the game, participant behavior shows signs of fatigue (recall that the participant plays 15 sessions overall and the game lasts for about 30 min), and as a result, the participant starts betting with positive probability in the sessions with an apprentice. Critically, under both hypotheses, the strength of the gambling bias increases with session number (the bias should be absent in the first sessions of the game and maximal in the final sessions), whereas the opposite is true for the bias generated by craving (see the “Effect of training on the bias” section). Thus, probing the dynamics of the bias exhibited by the task participants across sessions is a litmus test of both hypotheses against the CbD hypothesis.

#### The bias could reflect the thrill of gambling

The picking pennies bias could be nonconsequentialist in nature, in the sense that penny-pickers would choose to gamble when it is “perilous” (in the sessions with an apprentice) because this brings them “thrill” irrespective of the monetary outcomes (*16*). To test this hypothesis against the CbD hypothesis, we exploited the idea that the bias caused by craving can be alleviated by allowing the participant to commit to skip in the sessions with an apprentice (see the “Overcoming the bias: Self-control and nudging” section). In contrast, under the thrill of gambling hypothesis, the participant is reluctant to commit to skip in the sessions with an apprentice since it “kills” the thrill—if anything, the agent may want to commit to bet, to increase the thrill. Therefore, the CbD hypothesis predicts a decreased prevalence rate of the bias in contexts where the agent can commit to skip, whereas the thrill of gambling hypothesis predicts no effect of the commitment device on the prevalence of the bias—and possibly a positive effect if both a commitment to skip and a commitment to bet is offered to the agent.

This motivated us to run experiment 4 (*N* = 60; same cohort as in the previous experiments), an augmented version of experiment 1 where during each trial, the participant has the option to commit to either bet in all remaining trials of the session or skip in all remaining trials of the session (Fig. 3D). The rule is that once the participant exercises the option, she is not allowed to change her mind thereafter. At odds with the thrill of gambling hypothesis, the CbD hypothesis predicts that if the task participants were to use the commitment device in experiment 4, which we expected in light of prior experimental findings (*64*), then there should be a reduced prevalence of the picking pennies bias in that experiment relative to that in experiments 1 and 3.

We designed the task interface and instructions for experiment 4 to avert “experimental demand effects” and other artifacts in participant behavior. For example, the participant could use the option feature merely because it is presented, or to speed up the pace of the task. The participant could also deviate from the optimal strategy because she feels bored or finds the solution of the game “too easy to be true” (*65*). Our design controls for these potential confounding factors (the detailed procedure is spelled out in Supplementary Text).

#### The bias could reflect limited liability

The picking pennies bias generated in the laboratory may have little external validity given that the laboratory participant faces limited liability: The worst outcome is $5 as per the laboratory rules. To assess the importance of the limited liability factor, we ran experiment 5 (*N* = 47; same cohort as in the previous experiments), which replicated the original experimental settings (for the learning version of the task: experiments 1 and 3) except for that the participants in experiment 5 incurred real monetary losses (up to $95, a meaningful amount relative to their standard of living) in case of negative performance in the task (for more details on the procedure, see Supplementary Text).

We reasoned that finding a decreased prevalence rate of the picking pennies bias when participants were exposed to real losses (experiment 5) relative to when they were not (experiments 1 and 3) would suggest that limited liability is a significant factor underlying the bias in experiments 1 and 3, while finding no significant decrease would suggest the opposite, given that we had satisfactory a priori power for this test—as in the other statistical tests run for the study (see table S15 and Supplementary Text for details).

**Fig. 4.**
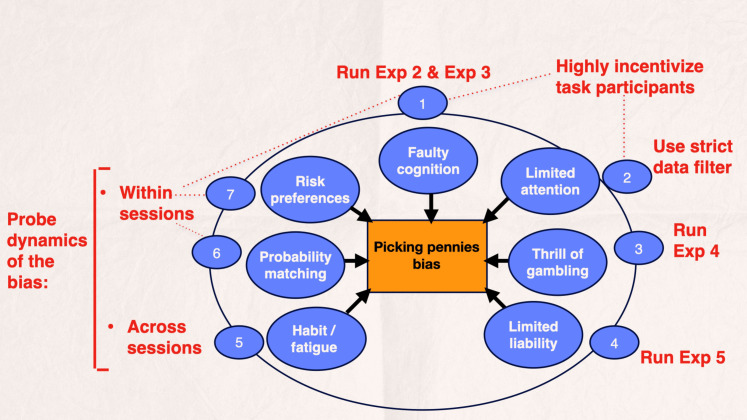
Explanations competing with the CbD hypothesis and experimental strategy to control for each. Seven categories of factors could lead participants to gamble in a session with an apprentice (“Picking pennies bias”): (1) Participants may be unaware that the expected value of gambling is negative. Specifically, they may (i) be unable to infer that the bowman is an apprentice in the learning version of the task, (ii) misunderstand the task instructions and as a result overestimate the probability of a winning bet with an apprentice, and (iii) exhibit the gambler’s fallacy. (2) Because of limited attention and limited memory, participants may sometimes forget that a black swan occurred earlier in the session. (3) Participants may want to experience the “thrill of gambling” during the task. (4) The bias could reflect the fact that a participant’s worst-case take-home earnings are $5. (5) Participants may use a model-free instrumental approach to perform the task, and their behavior could become partly habitual at some point during the game. They may also become fatigued. (6) Participant behavior could follow the probability matching rule. (7) Participants could be risk/loss tolerant. We controlled for each hypothesis by comparing the prevalence of the bias in experiment 1 versus experiment 2 and running experiment 3 [to assess (1i)]; incentivizing participants to pay attention during the task instructions and the game itself [to address (1) and (2)]; checking whether the bias still prevails in the subsample after applying the filter described in the main text to sift out instances of the bias that could arise from participant oversight, forgetting, and “trembling” [to address (2)]; running experiment 4 [to address (3)]; running experiment 5 [to address (4)]; and studying the dynamics of the bias within sessions—to see when it starts [to address (1ii), (1iii), (6), and (7)], and across sessions—to see how it evolves as the game progresses [to address (5)].

Figure 4 provides a visual summary of our experimental strategy. We reasoned that in this experimental setting, if we were to consistently observe the picking pennies bias across all experimental treatments except for that in experiment 4, and if the measured bias further exhibited the unique dynamics described by the CbD model, this would provide evidence for the CbD hypothesis.

### Evidence for the CbD hypothesis

#### The picking pennies bias is a significant and robust pattern of participant behavior in both versions of the task

Table 1 shows that in the no ambiguity version of the task (experiment 2), the bias is observed in 52% of participants, and in the learning version, it is consistently high across experiments except for experiment 4 (as predicted by the CbD hypothesis—we examine experiment 4 separately below), with an average prevalence rate of 54%. The table further shows that within participants, the bias is often observed in more than two sessions during the game. For example, in the no ambiguity version of the task, about 78% of the penny-pickers exhibited the bias in at least three sessions.

**Table 1.** Prevalence of the picking pennies bias in each experiment. The table shows the percentage of penny-pickers among the task participants and the percentage of penny-pickers who pick pennies in at least two, three, and four sessions, for each experiment. The mean number of sessions in which the penny-picking behavior is observed is also reported (as well as the SD) for each experiment.

	Percentage of penny-pickers	Penny picking frequency				
		Pick pennies in at least (%):			Mean	SD
2 sessions	3 sessions	4 sessions				
Experiment 1	59.7	70.3	44.6	32.4	2.5	1.6
Experiment 2	52.3	91.3	78.3	47.8	3.3	1.9
Experiment 3	44.6	96.7	73.9	60.9	4.4	2.4
Experiment 4	33.3	85	65	35	2.7	1.7
Experiment 5	51.1	83.3	75	54.2	3.6	2

For the learning version of the task, we derived conservative estimates for the prevalence of the bias, applying the data filter that corrects for the limited attention/memory confounding factor (Fig. 4, #2) and further restricting the sample to “big black swans” to account for the possibility that penny-pickers failed to recognize that a shot falling more than 7 m away from the target can only happen with an apprentice bowman (Fig. 4, #1i). To control for this possibility, we increased the threshold used to define a black swan to 8 and 9 m in two separate analyses. The estimated percentage of penny-pickers remains significant in the subsample, with an average of 36% across experiments under the most conservative criterion to measure the bias (table S2). It appears, therefore, that the bias is not reducible to limited attention/memory or faulty learning of the bowman type. (N.B., We checked that the conclusions of all the analyses below hold across all measurements for the picking pennies bias.)

#### Ruling out explanations for the bias that compete with the CbD hypothesis

To strengthen the evidence that the bias is not reducible to faulty learning by the penny-pickers, we compared the prevalence rate of the bias when learning was involved in the task (experiments 1, 3, and 5) and when it was not (experiment 2). The null hypothesis that the prevalence rate of the bias is the same cannot be rejected [χ^2^ test for proportions: χ^2^(1) = 0.39, *p* = 0.53, *V* = 0.05], despite satisfactory power for this test (see table S15 and Supplementary Text). This finding suggests that faulty learning is not among the root causes of the bias in the current gambling paradigm. It may nonetheless be a factor in the study of interindividual differences in the propensity for the bias. One can reject the null hypothesis of similar accuracy (defined as the fraction of correct replies regarding the bowman type) between the penny-pickers and the other participants in a two-sample *t* test [mean accuracy for the penny-pickers, 0.83; for the other participants, 0.91; *t*(97) *=* 4.67, 95% confidence interval = (0.056 to 0.134), *p* < 0.001, *D* = 0.99].

**Fig. 5.**
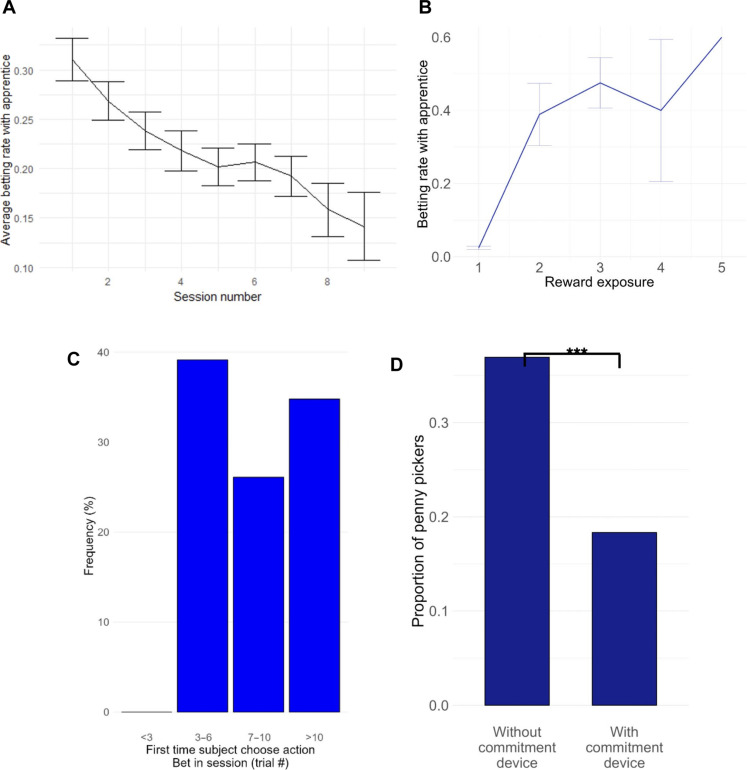
Main experimental findings. (**A**) The probability of the bias decreases over time. The graph reports the betting rate of the penny-pickers in sessions with an apprentice, averaged at the session level, as a function of session number, pooling the data from all the experiments run for the study. Error bars show SEM. (**B**) The probability of the bias increases with reward exposure. *X* axis: Reward frequency in the previous trials. The variable was split into five bins of equal range. Error bars show SEM. *Y* axis: Betting rate in the sessions with an apprentice computed across all sessions and task participants in the “no ambiguity” version of the task (experiment 2). (**C**) The graph shows the distribution of first instances where penny-pickers bet with an apprentice in a session as an average, computed at the participant level using the data for experiment 2. For the equivalent of (B) and (C) for the learning version of the task, see fig. S1. (**D**) The proportion of penny-pickers is significantly smaller in experiment 4 than in experiments 1 and 3 according to a χ^2^ test (* < 0.10, ** < 0.05, and *** < 0.01, see the main text for the detailed statistics). The proportions reported on the graph are those obtained under the conservative measurement of the picking pennies bias (table S2), without loss of generality (the conclusion is the same with the other measurements).

The penny-pickers did not see more black swans than the other task participants did (table S3). In each experiment, the following key patterns hold: Across sessions, the probability to pick pennies decreases over time (Fig. 5A); within sessions, the probability increases with the Pavlovian cumulative reward value (Fig. 5B). The penny-pickers typically skipped in the first trials of each session with an apprentice (in all experiments) and also in the sessions with a master for those performing the learning version of the task (Fig. 5C and figs. S1 to S4). The bias started occurring quite late in the session (table S4). Together, these findings are consistent with the CbD hypothesis but inconsistent with several of the competing hypotheses for the bias listed in Fig. 4, namely, habit, risk preferences, underestimation of the probability of a winning bet with an apprentice, and probability matching.

As for the gambler’s fallacy (Fig. 4, #1iii), we should recall that if it was among the root causes of the picking pennies bias, a significant portion of penny-pickers would choose to bet immediately after the occurrence of a black swan. Only a minority of them did (fig. S5, type 1). An agent susceptible to the fallacy would also tend to skip after a series of good outcomes (mistakenly thinking that a tail event is “due”), which is at odds with actual behavior (Fig. 5B).

Next, we found that the thrill of gambling explanation for the bias (Fig. 4, #3) is falsified by the findings obtained in experiment 4. Many of the participants (48 of 60) used the option to lock in their decision, and the option to skip was predominantly used. Specifically, 26 subjects exclusively used the option to skip in all remaining trials of the session (henceforth, “skip-in-all”), while only 2 participants exclusively used “bet-in-all.” Among the 20 participants who used both options, skip-in-all was used 4.85 times on average (median, 5 times; mode, 3 times), whereas bet-in-all was used only 2.55 times on average (median, 2; mode, 1). Such asymmetry (higher popularity of the option to skip) casts doubt on the thrill of gambling hypothesis, and it also rules out the possibility that participants used the options simply because they found it easier to commit to a given course of action rather than having to deliberate on each trial. (For if they had used the options chiefly for that reason, the frequency of choosing bet-in-all would not markedly differ from that for skip-in-all.) The second key finding is that the proportion of penny-pickers is significantly smaller in experiment 4 than in experiments 1 and 3 [χ*^2^*(1) = 7.64, *p* = 0.006, *V =* 0.19; Fig. 5D]. Together, these findings provide evidence for the CbD hypothesis against the thrill of gambling hypothesis.

Last, we assessed the importance of the limited liability factor (Fig. 4, explanation #4) by comparing the prevalence rate of the bias when the participants were at risk of real and significant monetary losses from participating in the experiment (experiment 5) and when they were not (experiments 1 and 3). The null hypothesis that the proportion of penny-pickers is similar in the two cases cannot be rejected [χ^2^(1) = 0.11, *p* = 0.75, *V* = 0.03], despite satisfactory power for the test (table S15). This finding suggests that the prevalence of the bias in the first four experiments is not an artifact related to the limited downside risk to participants’ earnings in laboratory experiments.

The entire collection of findings is reinforced in regression analyses. We ran a set of logistic regressions predicting the picking pennies bias across participants as well as logistic mixed-effects regressions predicting the occurrence of the bias among penny-pickers (see Materials and Methods). We further found that the CbD model fits the penny-pickers’ behavior significantly better than the base model does (see Materials and Methods and fig. S10) by uniquely capturing key aspects of the penny-pickers’ behavior in the task (figs. S6 and S7).

Therefore, while no single piece of evidence is decisive, the CbD hypothesis offers a unified account for the broad set of experimental findings related to the picking pennies bias. The bias is consistently observed in contexts where the CbD model predicts its prevalence (experiments 1, 2, 3, and 5), and it is significantly decreased in the specific context where the model predicts a decreased prevalence (experiment 4). Furthermore, the dynamics of the bias observed in the laboratory appears to “match” the distinctive patterns of the bias generated by craving in the CbD model.

#### Penny-pickers bet more than the other participants in sessions with a master bowman

Also consistent with the CbD hypothesis, we found that in sessions with a master, the penny-pickers bet more than the other participants. The mean difference in betting rates between the two groups is significant according to a *t* test run at the participant level [mean difference: 0.173; *t*(218.51) = 9.021, *p* < 0 0.001, Cohen’s *D* = 1.198; data from experiments 1, 3, and 5 pooled; the results are similar for experiment 2].

#### Summary of experimental results

The findings thus support the idea that the Pavlovian injunction (“engage with the gambling cue”) boosts the probability of gambling when it is optimal (in the sessions with a master) while leading the penny-pickers astray when the instrumental and Pavlovian systems are in conflict (in the sessions with an apprentice).

### Test of the CbD hypothesis in the field

#### Applying the CbD hypothesis to financial investing scenarios

Recall that in the CbD model, the agent susceptible to craving knows that the expected value of the negatively skewed lottery is negative, yet she wants to take the lottery because of the frequent rewards attained. Appraised in the context of financial investing, this suggests that the pitfall of negative skewness spares no one and even the most knowledgeable investors may be prone to the picking pennies bias, potentially resulting in asset-pricing distortions.

To be more specific, the CbD model, together with evidence that the purported Pavlovian bias affected a significant portion of our laboratory participants, points to the possibility that the marginal trader knowingly engages in a disadvantageous trade because of a craving for the trading cue (the asset being traded). Assume for example that the returns from buying an asset feature negative skew and negative expected value as seen in the lottery *L* in the CbD model (see the “Case where the Pavlovian and instrumental systems are in conflict: Picking pennies bias” section). Recall that in such a case, when the craving factor *DA* is large enough, i.e., after repeatedly observing a good outcome with *L*, the agent may take *L* despite knowing that doing so has negative expected value. Similarly, here, after frequently recording good outcomes from buying the asset in the recent past, the trader may want to buy the asset despite knowing that the expected value of doing so is negative. (In the language of the model, if/when the Pavlovian value of the buying cue becomes large enough, the trader may crave for it.) Likewise, in contexts where selling an asset yields outcomes akin to the outcome profile of lottery *L* in the CbD model (negative expected value, negative skew), the trader may develop a craving for the selling cue.

Crucially, in both the CbD model and the experimental paradigm used for the study, the negative expected value of *L* is exogenous (it does not come from the agent’s behavior), whereas in a market context the negative expected value reflects the craving phenomenon itself. To appreciate this, assume that craving affects the buyers of a given asset. Their craving will cause an upward pressure on the equilibrium asset price (since they increase their demand for the asset as a result of craving it). If this pressure offsets countervailing forces from the selling side of the market, craving will cause a price increase (i.e., it will reduce the expected value of buying the asset). Likewise, if craving affects the sellers of the asset, it will exert a downward pressure on the equilibrium asset price, which may offset countervailing forces from the demand side, resulting in a decrease in value from selling the asset. Assuming the expected value in the absence of craving is positive, it is possible that craving turns the value from positive to negative.

#### Empirical strategy to test the CbD hypothesis with financial data

In the final stage of the study, we aimed to test this application of the CbD hypothesis in settings where investor craving can disrupt the asset price in the sense previously described. To control for the faulty cognition factor (applying the same logic we used in the laboratory), we chose the options markets as the setting for our tests. We leveraged the fact that the average options trader is more knowledgeable than the ordinary investor, and within the options markets, the sellers are thought to be particularly knowledgeable (see Materials and Methods). Therefore, we chose to narrow our focus to this specific group of agents in our tests of the CbD hypothesis. We reasoned that it is highly likely that options sellers who engage in an investment that has consistently yielded negative outcomes are aware their investment has negative value. The likelihood of this being true should be even higher if we exclude retail investors from the analysis and only focus on the most knowledgeable group of investors (more below).

It is well known in financial economics that options selling normally has positive expected value (see Materials and Methods). So we asked: If options sellers were prone to craving a specific category of options, and if this craving were strong enough, could it potentially turn the expected value from selling these options from positive to negative? Both the CbD model and the evidence from the laboratory point in this direction. Therefore, in the next stage of building our empirical strategy, we tried to maximize the chance of observing this purported options-pricing anomaly (options selling having negative expected value) by identifying the appropriate category of options to focus on in our tests.

Current financial economics literature compels us to focus on call options specifically, as the anomaly is unlikely to exist with put options. (The buying pressure is too large for them, so even though seller craving would depress put option prices, it would not cause the expected value from selling them to turn negative; see Materials and Methods.)

Within the category of call options, the CbD model compels us to narrow our focus to call options with the highest craving power for the sellers, in the sense that selling these options often yields good payoffs, and the median payoff is large (see the “Pavlovian value and craving power of a gambling cue” section). This prompted us to focus specifically on call options priced below $1, which we refer to as “the cheap calls,” as they fit the required profile. Selling the cheap calls indeed yields larger median rewards than selling those above $1, along with a higher reward probability (see Materials and Methods). Therefore, the chance that seller craving is strong enough to result in a negative expected value for the seller should be greatest with the cheap calls.

**Fig. 6.**
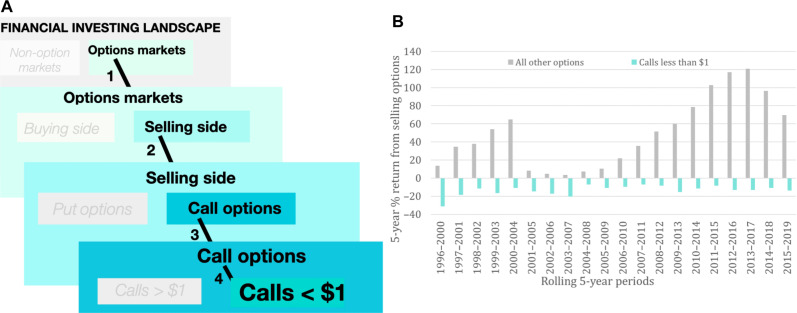
Summary of empirical strategy and main discovery. (**A**) To maximize the chance of observing the purported asset-price distortions generated by craving, we chose to (1) focus on options markets because, in the finance community, it is believed that options traders are more knowledgeable than non-option investors; (2) within options markets, focus on the selling side because options sellers are believed to be more knowledgeable than buyers; (3) focus on selling call options specifically because it is unlikely that craving may generate price distortions of put options; and (4) focus on selling call options priced below $1 (so-called “cheap calls”) because, compared to selling other call options, it has higher craving power according to the CbD model. (**B**) The graph reports the 5-year rolling average dollar returns from cheap call selling (green) versus selling all other options (gray). Cheap call selling has consistently had negative expected value (“the cheap call selling anomaly”).

Thus, combining knowledge from financial economics and the CbD model led us to test for the idea that selling cheap calls has negative expected value (henceforth, “the cheap call selling anomaly”). A visual summary of our empirical strategy is provided in Fig. 6A.

#### Evidence for the cheap call selling anomaly

To test for the cheap call selling anomaly, we used all options in the Optionmetrics database from 1996 to June 2019 and computed returns from buying and selling options—both for all options pooled together and for the call options separately (see Materials and Methods). The findings point to the existence of the cheap call selling anomaly. We indeed found that options selling provides positive value, negatively skewed payoffs (reinforcing well-established results) except for the cheap calls: Selling these calls has negative expected value (Fig. 6B and table S6). The results of an extensive set of fixed-effects regressions (to control for firm specific variables and option specific characteristics) strengthen the evidence that the pattern of negative expected value from cheap call selling exists, and that it is unique to the cheap calls (see Materials and Methods).

We also provide evidence that the demand for the cheap calls is high and that the cheap call selling anomaly reflects the presence of selling pressure on the cheap calls (see Materials and Methods). We further found a positive relationship between the degree of craving power of a call option for sellers and the size of the discount from selling this call (fig. S8 and Materials and Methods). This finding is consistent with the idea that the observed selling pressure on the price of the cheap calls is caused by craving in the sellers of these calls.

Relatedly, turning to the nonstandard options markets to assess the generalizability of our findings, we identified the equivalent of cheap call selling in this case, i.e., nonstandard options featuring the same craving power for sellers as with standard cheap call selling—and found the same anomaly (see Materials and Methods). This finding suggests that what makes the cheap calls “special” (anomalous) is their craving power for sellers rather than some other characteristic.

To further test the idea that the cheap call selling anomaly comes from seller craving, we examined conflicting explanations for the anomaly. First, we speculated on whether the anomaly could reflect faulty cognition in the sellers of the cheap calls—they might mistakenly think that selling them has positive value. Recall that we chose to focus on the selling side of the options markets because options sellers are thought to be generally knowledgeable (Fig. 6); admittedly though, it is unlikely that this applies to all options sellers, and one would expect significant heterogeneity among this group. Therefore, we examined, using the data provided by the International Securities Exchange (ISE) from May 2005 to 2019, whether cheap call selling comes more from the class of “customers”—encompassing both retail investors and knowledgeable traders such as hedge funds, or from the class of “firms”—which only includes knowledgeable traders. We reasoned that if we were to attribute the anomaly primarily to trades by agents from the customer class, we would surmise that the anomaly largely reflected limited knowledge about cheap call returns, rather than craving. Finding otherwise would suggest that the anomaly is not reducible to faulty cognition.

We further found that the widely employed covered call strategy (i.e., being long on a stock while selling a call based on this stock) using the cheap calls has consistently yielded lower returns than merely being long on the underlying stock, in contrast to covered call strategies using other call options (fig. S9). Given that it is most likely that cheap call selling is part of a covered call strategy in many (if not most) cases, this finding makes it difficult to believe that the sellers of the cheap calls ignored the negative value of their selling.

Last, we considered the possibility that cheap call selling is rational (i.e., it has positive instrumental value for the sellers). We explored three categories of scenarios: (i) Cheap call selling could be part of a hedging strategy, (ii) it could be part of a trading strategy aimed to provide liquidity across a range of options (for firms), and (iii) it could be part of a strategy to achieve higher “Sharpe ratios.” (The Sharpe ratio is one of the most widely used methods for measuring risk-adjusted relative returns in the investment management industry.) Several aspects of our dataset point against each scenario (see Supplementary Text). We concluded, therefore, that the possibility that the anomaly has a rational origin is not the most likely one.

## DISCUSSION

To summarize, the current study applies to the gambling domain the well-established idea that the Pavlovian system can sometimes disrupt instrumental behavior. From a neurobiological perspective, the penny-picker erroneously betting in response to a betting cue associated with monetary rewards is comparable to the rat approaching a light predictive of food even when that leads to food omission [in the seminal paradigm of (*6*)]. Both scenarios epitomize Pavlovian misbehavior. In addition, closely resembling our study were findings in a Go/NoGo task where human subjects erroneously acted in response to reward-predictive stimuli when they should have withheld action (*8*).

Notably, the extent of Pavlovian misbehavior appeared to decrease in a variant of the Go/NoGo paradigm where the participants were informed of the action contingencies for each fractal image used in the task (*66*). In contrast, the purported Pavlovian bias appeared to be equally present in the “no ambiguity” and learning versions of our gambling paradigm. These dissimilarities suggest important differences in the nature of the two paradigms and the purported Pavlovian influence in each one. As such, the present study adds to the psychology literature by generalizing the Pavlovian influence evidenced in the pioneering Go/NoGo studies (*8*–*10*, *66*) to other forms of Pavlovian biases [as called for by the authors of these studies (*10*)].

Our study also contributes to the financial economics literature. First, we provide an answer to the puzzle mentioned in Introduction, which is that harmful [“exuberant” (*20*)] risk-taking in economic decision-making appears to be too ubiquitous to be reducible to faulty cognition under imperfection information (*21*). Here, we show that even a perfectly informed agent can exhibit harmful risk-taking because Pavlovian misbehavior affects everyone, including the most knowledgeable people. Bringing together this idea and well-established knowledge from financial economics led us to discover the cheap call selling anomaly. Our empirical findings suggest that the sellers of the cheap calls are like the penny-pickers in the CbD model and laboratory experiments we conducted.

That said, it is worth emphasizing certain limitations of our study. First, it is important to stress that the evidence for Pavlovian influences on gambling decisions is only suggestive. An important question here is external validity, in particular, the extent to which the experimental findings reflect a true effect rather than a false positive. To help resolve this, we reported that the picking pennies bias was replicated in identical laboratory conditions but with a different population, different experimenters, and in a different country (table S20 and the Supplementary Materials). The next objective for researchers would be to generalize the findings obtained in our gambling paradigm by testing for Pavlovian misbehavior in other gambling tasks.

We also caution against interpreting our experimental findings as an indication that the factors summarized in Fig. 4 do not matter in the field. The findings suggest only that the picking pennies bias is not reducible to these factors and that Pavlovian misbehavior is another factor to consider to identify the root causes of the bias in the field. The degree of importance of the different factors underlying the bias, and how they combine in the field, is an important question for future research. For example, in contexts where people face significant downside risk, the purported impact of craving on gambling may sometimes be offset by the fear of an occasional massive loss. In contrast, in contexts where such fear is diminished due to limited liability, this liability factor and craving would “conspire” to generate harmful gambling. Our experimental design did not allow us to further explore this interaction because manipulating the liability factor is inherently limited in laboratory experiments given current ethics requirements. (It should be noted that even in the final experiment run for the study, liability was increased though still limited.) However, perhaps such manipulations could be implemented in field studies.

The study raises many other questions for future research. For example, the experiments run for the study were not designed to probe the mechanisms underlying the purported Pavlovian bias in gambling. In light of the literature on Pavlovian influences, two obvious mechanisms to consider are “sign-tracking” [also known as “autoshaping”—the tendency to treat a cue predicting reward as if it is the reward itself (*11*, *42*)] and the “Pavlovian-instrumental transfer” [when a Pavlovian cue for a reward induces an animal to perform more avidly an instrumental action previously associated with that reward (*67*)]. Harmful gambling caused by the Pavlovian system could come from either mechanism or a combination of the two. Addressing this question will require designing more sophisticated laboratory experiments in future research.

It would also be interesting to examine whether financial investors will consciously adapt their decision-making to overcome the vagaries of Pavlovian influences after dissemination of our research, just as the dissemination of past research on stock market “anomalies” has reduced the extent to which such anomalies are observed (*68*). While it does not seem possible to prevent our brains from being susceptible to Pavlovian influences (*9*), learning about their existence could make it possible for people to consciously correct their decisions. There is some suggestion that self-control might work, and indeed, the evidence suggests that in some people the inferior frontal gyrus can successfully inhibit Pavlovian influences (*9*). Another route to overcome Pavlovian misbehavior could be “self-nudging” (*69*). For example, once they have been made aware of the gambling bias generated by craving, people may be able to successfully employ decision-making tools such as the commitment device offered to the participants in the fourth experiment in this study. If that were to be the case, then it may motivate researchers to identify other manifestations of Pavlovian misbehavior in everyday life in order to help people protect themselves against Pavlovian influences across domains—yet another potential area for future research.

## MATERIALS AND METHODS

### Experimental design

All the laboratory experiments documented in the article were approved by the UNSW Human Research Ethics Advisory Panel, and written consent was obtained from all task participants.

### Statistical analysis (experimental data)

#### Regressions

We ran a logistic regression predicting the picking pennies bias in all five experiments (table S7). The independent variables include dummy codes for experiments 1 to 5, the total number of black swans seen throughout the experiment, and whether the participant is more likely to start betting early, late, or never in a session. We ran three models, each using a different threshold value to define a black swan event (7, 8, and 9 m from target).

It appears that the participants were less likely to pick pennies in experiment 4 compared to experiment 1, confirming the findings documented in the main text regarding the effect of the commitment device on the prevalence rate of the bias. There is no effect of the total number of black swans seen, which confirms that the penny-pickers saw the same number of black swans as the other participants did on average. The results further confirm that the penny-pickers were not more likely to bet early than other task participants.

In a complementary logistic regression ascertaining the proportion of sessions in which participants correctly guessed the bowman type in experiments 3 and 5 (“accuracy”), we found no effect of accuracy except under the 7 m criterion to measure penny picking (table S8).

Next, we ran a logistic mixed-effects model predicting betting in sessions with an apprentice after a black swan is seen (table S9). The independent variables are the foregoing experiment dummies, the time from black swan occurrence (“first bet”), the decision in the previous trial, the current net accumulated outcomes (“wealth”), the mean outcome from the last five trials, the session number, and how far in the tail the black swan occurred. We also ran a complementary model that predicts betting decisions using data from all participants and all sessions to compare the behavior of the penny-pickers to that of the other task participants (table S10). That model augments the previous mixed-effects model with variables for bowman type and participant type (penny-pickers versus others) along with interaction terms for penny picking and the previous decision, wealth, the last five outcomes encountered, and session number.

It appears that betting at time *t* − 1 increased the chance of betting at time *t* (table S9); however, the effect is not exclusive to the penny-pickers and is more prevalent in the other task participants (table S10). These findings suggest that although “choice stickiness” [the tendency to persevere in a given course of action (*70*)] might have influenced participant behavior, it is unlikely to be a major factor underlying penny picking. The prevalence of the bias seems unrelated to the feeling of being “in the red” (table S9, variable wealth), which suggests that our payment rule (which aimed to prevent wealth effects from influencing participant behavior) worked. The results confirm that the likelihood of the picking pennies bias decreases over time (table S9, variable “Session number”) and increases with reward exposure (table S9, variable “Last 5 outcomes”), as predicted by the CbD model.

#### Model comparison analyses

We studied how well the CbD model fits participant behavior in the task relative to the base behavioral model—which does not allow for craving. The free parameters of the models are β, α_1_, α_2_, α_3_, and λ (base model) and β, α_1_, α_2_, α_3_, λ, κ_1_, κ_2_, and θ (CbD model). For each task participant, we fitted the free parameters of each model by maximizing the log-likelihood (LL) computed over the set of trials for the first eight sessions of the task (optimization step). The ensuing parameter estimates were then used to predict the participant’s choices in the last seven sessions (out-of-sample validation step). The goodness-of-fit of the model was measured either by the negative LL out of sample or by the percentage of correct action predictions out of sample (for the latter method, model behavior was simulated using the predicted choice probability to generate one action on each trial). We checked that the main conclusions hold under either method. We found that the CbD model fits the penny-pickers’ behavior significantly better than the base model (paired *t* test comparing the negative LL out of sample of the CbD model to the one of the base model: *N* = 120, *t =* −9.46; *p* < 0.0000001, two-tailed; Cohen’s *D =* −0.86; fig. S10A). We checked that the procedure has satisfactory model recovery (fig. S11) and ran an extensive set of robustness checks. For example, we reran the analysis under the assumption that task participants were risk neutral (α_1_, α_2_, α_3_, and λ are set to 1). Whichever setting we used, the conclusion was always that the CbD model fits the penny-pickers’ behavior significantly better than the base model (see Supplementary Text).

### Empirical strategy

#### Options sellers are likely to be aware of the performance of their trading

Evidence suggests that options trading requires constant performance monitoring, resulting in option prices containing more information and reacting more quickly to new information relative to other asset categories (*71*). Evidence further suggests that within options markets, sellers are thought to be more knowledgeable than buyers on average (*72*, *73*).

#### The purported options pricing anomaly is unlikely to occur with put options, but it may occur with call options

The options pricing anomaly posited in the main text can occur only if the selling pressure caused by seller craving dominates the buying pressure (in short, “supply > demand”). Financial economists do not expect to observe this with put options given the strong demand for insurance against downside risk that underlies put buying (and corollary volatility premium embedded in puts) [see (*74*, *75*), among many others]. The demand for insurance is, however, significantly lower for call options.

### Statistical analysis (financial data)

We compiled all options trades in the Optionmetrics database from 1996 to June 2019. The sample for a given day contains all traded options with a nonzero bid price, a bid-ask spread between 0 and 25% (removing negative and wide bid-ask spreads is necessary to account for potential data errors and unreasonable—nontradable—prices), and a maturity less than 60 days. (We analyzed options with a maturity above 60 days separately, and all the results reported below conform to that sample.) For each option *i* in the sample, we computed both the standard return metric

$${\text{MID}}_{i,t}=\frac{\text{Mid}\;{\text{Price}}_{i,T}-\text{Mid}\;{\text{Price}}_{i,t}}{\text{Mid}\;{\text{Price}}_{i,t}}\;$$ (8)

where Mid Price*_i_* denotes the mid-point price of option *i* (the average of the bid- and ask-price) and *T* is the close of the last day of trading (*T > t*), and the following “corrected” returns that incorporate transaction costs

$${\text{SELL}}_{i,t}=\frac{\text{Ask}\;{\text{Price}}_{i,T}-\text{Bid}\;{\text{Price}}_{i,t}}{\text{Bid}\;{\text{Price}}_{i,t}}$$ (9)

$${\text{BUY}}_{i,t}=\frac{\text{Bid}\;{\text{Price}}_{i,T}-\text{Ask}\;{\text{Price}}_{i,t}}{\text{Ask}\;{\text{Price}}_{i,t}}$$ (10)

Ask Price*_i_* and Bid Price*_i_* denote option *i* ask price and bid price, respectively. To account for the possibility that the percent returns are driven by outliers, we also compute the dollar return metrics Mid Price_*i*,*T*_ − Mid Price_*i*,*t*_, Ask Price_*i*,*T*_ − Ask Price_*i*,*t*_, and Bid Price_*i*,*T*_ − Bid Price_*i*,*t*_ for MID, SELL, and BUY dollar returns, respectively. One can think of the MID return as reflecting the balance of supply and demand before execution, and the SELL (resp. BUY) return as reflecting the price at which sellers (resp. buyers) are willing to trade the option. Overall, there are 183,983,273 option observations in the sample, 17,071,406 unique options, and 9723 unique firms.

#### Replication of well-established findings

Table S6 shows descriptive statistics of returns from a long position in the option (MID and BUY returns) and from a short position (SELL returns), both for all options pooled together, and for the call options only. (The results for puts only are qualitatively the same as the results for all options pooled so we do not show them here). For all options pooled together, the average and median MID returns are −8.76 and −68.43%, respectively. The average MID dollar return is −$0.97, and SKEW > 0. These findings confirm the established evidence that options buying (resp. selling) provides EV < 0 (resp. EV > 0) and SKEW > 0 (resp. SKEW < 0). See, e.g., (*76*, *77*), among others. In our data, this remains true after incorporating transaction costs: BUY (resp. SELL) returns are significantly negative (resp. positive) with SKEW > 0 (resp. SKEW < 0).

#### Cheap call selling anomaly

The picture is very different when focusing on the cheap calls (table S6, middle column): Their average SELL returns are negative (−23.81%) and negatively skewed (SKEW = −13.32). Notably, even without incorporating transaction costs, cheap call selling results in *EV <* 0 (the average MID returns are indeed 7.41%, implying negative average returns from shorting these options). Notably, the average BUY returns of the cheap calls are also negative, implying that once transaction costs are incorporated, cheap call trading results in an average expected loss for both buyers and sellers.

#### Selling cheap calls has high craving power in the sense of the CbD model relative to selling other calls

Both reward size and reward probability are higher with cheap call selling. The median returns from cheap call selling are 88% versus only 44% from selling calls above $1. Moreover, cheap call selling ends up profiting the seller 70.4% of the time versus 40.2% of the time with the other call options. See table S6 and fig. S8.

#### Evidence for a selling pressure on the cheap calls

We computed the difference between SELL and MID returns, a method used in previous work to measure the average selling pressure (*78*). The difference is −31.22% (or −16c) for the cheap calls versus 7.78% for the other calls (table S6), which is indicative of a selling pressure producing 31.22% more negative returns from cheap call selling, and an absence of selling pressure in the case of the other calls. More evidence for a selling pressure affecting the cheap calls price is provided in Supplementary Text.

#### Evidence that the size of the anomaly increases with the craving power of the option

As shown in the “Pavlovian value and craving power of a gambling cue” section, the larger the potential reward and the higher the likelihood of being rewarded, the higher the craving power of the Pavlovian cue. Used in the financial investment environment, this means that the larger the median returns from the investment and the higher the likelihood of getting good returns, the higher the craving power of the investment cue. Given that the seller of a call wins when the call expires out of the money, the craving power associated with call selling increases with the likelihood of the call finishing out of the money. Therefore, a natural metric to measure the degree of a call’s craving power for sellers is the likelihood of the call finishing out of the money × the median return from selling the call. Figure S8 shows that there is a positive relationship between the degree of a call’s craving power for sellers and the expected value reduction from selling such calls.

#### Fixed-effects regressions

We ran fixed-effects regressions, controlling for firm-specific and options-specific characteristics, using the methodology followed in many prior financial economics studies (*79*)

$$\begin{array}{c} R_{i,t}=\alpha_{i,t}+\beta_{1}{\text{MP}}_{i,t}+\beta_{2}\Delta_{i,t}+\beta_{3}{\text{IV}}_{i,t}+\beta_{4}{\text{Mat}}_{i,t}+\beta_{5}{\left\{\text{MP}<1\right\}}_{i,t}\\ +\beta_{6}{\text{BM}}_{i,t}+\beta_{7}{\text{Size}}_{i,t}+\beta_{8}1{\text{YR}}_{i,t}+\beta_{9}{\text{SP}}_{i,t}+\epsilon_{i,t} \end{array}$$ (11)

where *R*_*i*,*t*_ is either of MID_*i*,*t*_, SELL_*i*,*t*_, and BUY_*i*,*t*_ (we ran three separate regressions). The variables MP_*i*,*t*_, ∆_*i*,*t*_, and IV_*i*,*t*_ respectively denote the price, delta (the price sensitivity of an option to the change in price of the underlying asset), and implied volatility of option *i* on day *t*. Mat_*i*,*t*_, 1YR_*i*,*t*_, BM_*i*,*t*_, and Size_*i*,*t*_ denote remaining time to maturity, the prior 1 year (52-week) return, book-to-market ratio, and firm size. SP_*i*,*t*_ denotes the bid-ask spread (an important control here as the bid-ask spread directly affects selling profitability). {MP *<* 1}_*i*,*t*_, our main variable of interest, is a dichotomous variable that equals 1 if the price of option *i* is less than one dollar, and equals 0 otherwise. We found a positive (resp. negative) coefficient on this variable in the specification that uses the MID (resp. SELL) returns as dependent variable [table S11, regression (2), panels A and B]. We also found that the average predicted MID returns of the cheap calls are positive [computed from the coefficients on the independent variables reported in table S11, and the average values $\bar{\text{MP}}$ = 4.08, $\bar{\text{SIZE}}$ = 699*M*, $\bar{1Y\;R}$ = 2.94, $\bar{\text{BM}}$ = 0.39, $\bar{\text{∆}}$ = 0.54, $\bar{\text{IV}}$ = 0.47, $\bar{\text{Mat}}$ = 25.46, $\bar{\text{SP}}$ = 0.33], and that relative to selling the other calls, cheap call selling results in 31% worse returns (table S11A). As for the SELL specification, the average predicted SELL returns are negative for the cheap calls, whereas they are positive for the other calls (table S11B). These findings thus show that the pattern of negative EV from cheap call selling is specific to the cheap calls and presents itself consistently even after adding the necessary control variables. We ran a set of supplementary regressions to check the robustness of all these conclusions (see Supplementary Text).

#### The anomaly is also observed for options defined on negative beta assets

We reran the main analysis with options defined on negative beta assets, that is, assets whose returns are negatively correlated to the equity markets (for example the priced index VIX, the so-called “market fear gauge” reflecting the volatility implied from option prices on the S&P 500). These options have a payoff structure that is opposite to that of traditional options, meaning that in this case, cheap puts have craving power for sellers. Therefore, there should be a “cheap put selling anomaly” in those markets if the anomaly truly reflects craving in the sellers. The results reported in table S12 support this prediction.

#### Evidence that firms are behind the cheap call selling anomaly to an even greater extent than customers

We used the data provided by the ISE from May 2005 to 2019 to determine the identity of the sellers of the cheap calls and the nature of the transactions underlying the cheap call selling anomaly. There are four types of transactions: open buy, closing buy, open sell, and closing sell. For example, if an investor opens a new position to buy 100 options on a given asset, the transaction is an open buy. If he then wants to sell these options that he previously bought, the transaction is a closing sell. Alternatively, if the investor wants to sell 100 options on a given asset to go “short,” the transaction is an open sell. If the investor wants then to close that short position by buying back 100 options, it is a closing buy transaction. It is important to distinguish between the four types as only open sell transactions may reflect the craving motive. (Likewise, only open buy—not closing buy—transactions can be tied to the gambling motive.) The ISE data provide access to the daily volume of each transaction type for all individual options, separated by trader class. This allowed us to examine if cheap-call open sell transactions come more from the class of customers—which encompasses both retail investors and knowledgeable traders such as hedge funds—or from the class of firms—which only includes knowledgeable traders (institutional or proprietary).

We found that firms engage more in cheap-call open sell transactions than customers do. Moreover, for firms, the volume of open sell transactions is larger than the volume of open buy transactions, whereas the reverse is true for customers (table S13). The evidence that the cheap call selling anomaly comes more from firms than from customers is strengthened when regressing the volume of open sell call transactions on all the independent variables used in our main regression for customers and firms separately. It appears that among the new open sell positions initiated by firms, there are 54 additional positions for the cheap calls relative to the other calls (table S14, coefficient of the {MP < 1} dummy); the corresponding number for customers (11 additional positions) is significantly lower [χ^2^(1) = 97.5, *p* < 0.0001].

## Supplementary Materials

### This PDF file includes:

Supplementary text Figs. S1 to S12 Tables S1 to S20 References
