# Supplementary material for: Craving money? Evidence from the laboratory and the field
Source: Sci Adv. 2024 Jan 12;10(2):eadi5034. doi: 10.1126/sciadv.adi5034 (PMC10786414; doi:10.1126/sciadv.adi5034)
Supplement: Supplementary file 1 — Supplementary text Figs. S1 to S12 Tables S1 to S20 References [file sciadv.adi5034_sm.pdf]

Science Advances
AAAS

Supplementary Materials for

Craving money? Evidence from the laboratory and the field

Elise Payzan-LeNestour and James Doran

Corresponding author: Elise Payzan-LeNestour, elise@unsw.edu.au

Sci. Adv. **10**, eadi5034 (2024)
DOI: 10.1126/sciadv.adi5034

This PDF file includes:

Supplementary text
Figs. S1 to S12
Tables S1 to S20
References

SUPPLEMENTARY TEXT

Alternative specifications of the CbD model

As part of checking the robustness of our findings, we ran the model comparison described in the main text with a version of the CbD model in which the Pavlovian influence is a fixed free parameter (DA), like in Rutledge et al. (*13, 14*). From the *n*-th exposure to the gambling cue *L*, the influence becomes positive and biases the probability of choosing *L* as described in the main text. That is, from the *n*-th trial, the probability of choosing *L* becomes:

$$P(L)^{\text{craving}} = DA + (1 - DA)\, P(L), \tag{S1}$$

where $0 < DA < 1$, and the value of $P(L)$ is given by Eq. (2) in the main text.

We checked that the results of the model comparison are qualitatively unchanged for different values of the parameter *n* (we ran the model comparison separately for *n* = 4,3,2).

We also checked that the main conclusions are unchanged when using a version of the model in which the Pavlovian influence modulates the instrumental value of the gambling action, rather than affecting choice probability.

We tested two models. In the first, the incentive salience parameter *DA* boosts the instrumental value in an additive fashion:

$$v(x)^{craving} = \begin{cases} (x + DA)^{\alpha} \; if \; x \geq 0 \\ -\lambda |x|^{\alpha} \; if \; x < 0 \end{cases}, \tag{S2}$$

with $DA > 0$. Assuming no probability weighting (for simplicity and without loss of generality) and solving $V(L) = 0$ using Eq. (1) in the main text and the foregoing specification for v^{craving}, one finds that if the craving parameter *DA* is above $\left(\frac{1-p}{p}\right)^{1/\alpha} \lambda^{1/\alpha}|y| - x$, the agent chooses L. Consider for example the negative EV lottery (2,.9;−40). The value of *DA* from which a risk/loss neutral agent chooses to take the lottery is around 2.4. For a loss averse and risk averse agent with $\alpha = .8$ and $\lambda = 2$, the threshold value is around 4. For a risk neutral and loss tolerant agent

with $\alpha = 1$ and $\lambda = .5$, it is around 1. In the second specification of the model, the craving parameter is multiplicative (like in Konova et al. (*80*), for example):

$$v(x)^{craving} = \begin{cases} (x \times DA)^{\alpha} \; if \; x \geq 0 \\ -\lambda|x|^{\alpha} \; if \; x < 0 \end{cases}, \quad \text{(S3)}$$

with $DA > 1$. Under this alternative specification, the threshold of DA from which the agent chooses L is $\left(\frac{1-p}{p}\right)^{1/\alpha}\left(\frac{\lambda}{x}\right)^{1/\alpha}|\gamma|$.

Computation of the estimated probability of a winning bet in the learning version of the experimental task

At each trial t of a given session, the agent compares the likelihood of the two possible models of the world (M_1 vs M_2) given the data available until (included) trial t $\underline{X_t} = (X_1, X_2, \cdots, X_t)$:

- If the bowman is a master (M_1), the data $\underline{X_t}$ have density $f_1(\underline{X_t}|\,\sigma)$:

$$f_1\left(\underline{X_t}\middle|\sigma\right) = \prod_{k=1}^{t} \frac{1}{\sqrt{(2\pi)}\sigma} e^{\left\{\frac{-X_k^2}{2\sigma^2}\right\}}, \quad \text{(S4)}$$

where σ is uniformly distributed between 0.1 and 2 by design.

- If the bowman is an apprentice (M_2), the data $\underline{X_t}$ have density $f_2(\underline{X_t})$:

$$f_2\left(\underline{X_t}\middle|\sigma\right) = \prod_{k=1}^{t} \frac{1}{\pi(X_k^2+1)}. \quad \text{(S5)}$$

At each trial, the agent assesses how likely it is that the session has a master bowman versus an apprentice bowman in light of the available data $\underline{X_t}$. The metric used is the "marginal or predictive density" (Berger and Pericchi, 2001) of $\underline{X_t}$ under each model (M_1 and M_2):

$$m_1\left(\underline{X_t}\right) = \int_{0.1}^{2} f_1\left(\underline{X_t}\middle|\sigma\right) \times \pi(\sigma)d\sigma, \quad \text{(S6)}$$

$$m_2\left(\underline{X_t}\right) = f_2(\underline{X_t}), \quad \text{(S7)}$$

where, $\pi(\sigma)$, the prior used for σ, reflects the fact that σ is uniformly distributed between 0.1 and 2: $\pi(\sigma) = \frac{1}{2-0.1}$. The posterior probability of each model given the data $\underline{X_t}$ is:

$$P\left(M_1|\underline{X_t}\right) = \frac{P(M_1)m_1\left(\underline{X_t}\right)}{P(M_1)m_1\left(\underline{X_t}\right)+P(M_2)m_2\left(\underline{X_t}\right)}, \tag{S8}$$

$$P\left(M_2|\underline{X_t}\right) = \frac{P(M_2)m_2\left(\underline{X_t}\right)}{P(M_1)m_1\left(\underline{X_t}\right)+P(M_2)m_2\left(\underline{X_t}\right)}, \tag{S9}$$

where $P(M_1)$ and $P(M_2)$ denote the prior probability of being in a session with a master and an apprentice, respectively. $P(M_1) = P(M_2) = 1/2$ by design (the participant knows that there are equal chances of facing an apprentice or a master in each session). At trial t, the probability of a winning bet is thus estimated to be:

$$p(t) = P(M_1 \mid \underline{X_{t-1}})\, p_g + P(M_2 \mid \underline{X_{t-1}})\, p_c\,, \tag{S10}$$

where p_g (resp. p_c) denotes the probability of a winning bet in a session with a master bowman (resp. apprentice bowman). To compute the value of p_g, one can first derive the standard deviation estimate $\tilde{\sigma}(t) = \sqrt{\frac{1}{t}\sum_{k=1}^{t} X_k^2}$. Using this estimate, one assesses the likelihood of a winning bet in a session with a master bowman to be

$$p_g \equiv p_g(t) = 1 - 2\left(1 - \Phi\left(\frac{4}{\tilde{\sigma}(t)}\right)\right). \tag{S11}$$

.

In the base behavioral model, the probability of a winning bet in a session with an apprentice bowman is (using the definition of the Cauchy density):

$$p_c = \frac{1}{\pi}\int_{-4}^{4} \frac{1}{x^2+1} dx = \frac{1}{\pi}[tan^{-1}(4) - tan^{-1}(-4)] = .844. \tag{S12}$$

In the analyses conducted for the study, we used approximations for the values of p_c and p_g (p_c = 0.8 and p_g = 1) to account for participant imprecise knowledge and rounding.

Special task instructions used in all the experiments run for the study

Animations. Animations or "distribution builders" showing 300 successive sample shots from master and apprentice bowmen were displayed on screen, allowing the task participants to grasp the shot distributions from the different kinds of bowmen through directly sampling many times from those distributions. (For a demo, see http://bowmangame.weebly.com/task-instructions.html.) Thus, all the participants could gather without having to engage in the explicit

computations (provided below) that the expected value of betting is positive with any kind of master bowman and negative with an apprentice, even those unfamiliar with basic statistics concepts such as expected value, probability, etc.

Provision of explicit statistics. For participants familiar with statistics concepts, a "FAQs" document (available at https://tinyurl.com/y5fmb4ns) distributed to all participants as part of the instructions for the task explicitly mentioned the key statistics for the task. The experimenter re-emphasized the fact that the expected value of betting is positive with any kind of master bowman and negative with an apprentice bowman when reviewing these FAQs with the task participants just before they started performing the task.

Evidence of effectiveness. Our main motivation for ensuring that the task participants grasped the stochastic structure of the task is that otherwise, they would likely underestimate the probability of losing with an apprentice, given people's natural tendency to ignore the probability of small probability disasters (*81*).The evidence suggests that the task participants did not underestimate the probability of losing with an apprentice but, if anything, *over*-estimated it (Fig. S10), consistent with the key finding documented in the main text that participants tended to systematically skip in the first trials of each session (they would have chosen to bet if they treated the probability of a losing bet with an apprentice as being negligible).

The participants were highly incentivized to perform well in the experimental task.

Payment rule. In each experiment run for the study, participant payoff from the task consisted of the participants' final net accumulated outcomes from all sessions, plus/minus a starting account balance. During the task instructions, the participants were explained that the account balance would be a value between −\$500 and \$500 that would be revealed after they have completed the task. They were further explained the rationale behind this feature, which is to avert wealth effects in participant behavior, since in each trial during the task, the participants did *not* know the current value of their wealth (their current net accumulated outcomes +/− the amount of the account balance). [Participants were told: "For scientific purposes, it is important that you don't know where you stand during the game. For example, imagine you lost several times and your current net accumulated outcomes are say −\$78. You may think you're in the red but you don't know that: if for instance the account balance has been set to say \$100, your current wealth in the game is

actually positive. Conversely, imagine you won a lot and your accumulated outcomes are $78. This does not mean your current wealth is positive: what if the current balance is −$100 for example (recall it is a number between −$500 and $500). Morale of the story: just focus on making the right choice on each trial.".] The experimenter set the amount of the account balance before the participants started performing the task by writing the amount on a sheet of paper and placing the sheet in an envelope in the middle of the lab room; all this was done in front of the task participants. (The experimenter randomly picked a number before each session based on participant behavior in the previous sessions, under the constraint that the expected cost of the session per participant shall be at least $20 per hour as per the lab guidelines.)

Payoffs were capped at $110 (due to budgetary constraints) and bounded below at $5 (the showup reward provided independent of task performance as per the lab protocol), except for that in Experiment 5, where the payoff lower bound was −$95 (a monetary loss of −$95, more below).

Rationale for using this rule. Such a payment rule provides task participants with high monetary incentives in two key regards. First, it generates a bimodal payoff distribution. For example, in Experiment 1, 59 out of 124 task participants won more than $100 (mean: $63, std: $42) vs. 40 ended up with the show-up reward of $5 (given independent of subject performance as per the lab protocol). The payoff gap between high and low task performers was even larger in Experiment 5 where bad performance can lead to negative payoffs (see next). Such bimodality of participant earnings was stressed both in the recruitment email and the task instructions, both for ethical reasons, and to ensure that the task participants had strong incentives to perform well in the task.

The second incentivizing aspect of such a rule is that it pays for the outcome from all decisions made, thereby averting the issue of "diluted incentives" arising with the alternative "pay one" approach (paying for the outcome of only a subset of the choices made), see (59). However, the "pay all" rule potentially presents the issue of giving rise to wealth effects. The account balance feature is to address this problem, as explained above.

Additional information regarding the experimental design used for the study.

Experiment 2. The task instructions used for Experiment 2 stress that the "*no deception rule*" is in effect in the experiment, to ensure that the task participants believe the information provided to them about the bowman type before each session begins.

Experiment 4. To avert "experimental demand effects" whereby participants in Experiment 4 may use the option feature merely because it is presented to them, the participants were told in the task instructions for Experiment 4 that using the option to bet/skip is not necessarily desirable, and that it is fine to choose to ignore that feature altogether. Furthermore, to avert that participants may use the option buttons merely to speed up the pace of the task, the instructions stressed that using the option does *not* accelerate the pace of the task in any way. To prove this point, a demo of the task was shown to the participants at the end of the instruction phase.

Averting participant boredom was our main motivation for using the learning version of the task (the one used in Experiments 1, 3, and 5) rather than the version in which no learning is involved (the one used in Experiment 2). With the latter, the optimal strategy in a session with an apprentice (use the 'skip in all' option at the first trial) is so obvious that participants may deviate from it merely as a result of feeling bored. It is also likely that some task participants would deem the optimal strategy "too easy to be true" and would start imagining scenarios to make the task more meaningful (64).

Experiment 5. The payoff rule described above was augmented with the following package: 1- Each participant came to an office (not the lab) and received a $100 monetary endowment two weeks before the experimental session. 2- The participants were told they were not to bring the endowment on the session day. 3- In case of negative earnings from the task, the participants had to pay off their debt to the experimenter up to $95. 4- The debt was to be paid within a week after the session date (by cash or banking transfer).

Features 3-4 imply that the participants in Experiment 5 were exposed to real losses relative to their standard of living. Feature 4 made it clear to the participants that not paying off their debt was not an option. (Participants were told that in case of misconduct, they would not be allowed to participate in any experiments in the lab in the future, and their main teacher would be informed of the misconduct. All the participants who ended up with negative earnings paid off their debt except for two participants whose data were therefore discarded.)

To ensure that Features 3-4 do not conflict with UNSW Research Ethics Committee rules, we introduced the money endowment (Feature 1). Our choice of the endowment amount of $100 corresponds to the maximal level achievable given budgetary constraints. Our motivation was to ensure that the amount was large enough to generate meaningful downside risk for the participants. For example, if the endowment was $50, the maximum possible loss from the task (to not infringe

the ethics committee's rules) would be "only" \$45. Setting it at \$100 means that participants can lose up to \$95 from the task. For the average undergraduate student, the prospect of losing \$95 matters relative to their conditions of living.

Feature 2 is to induce in the participants the feeling of playing with their own money. Not only the endowment is to be received several weeks before the session day (Feature 1); it is also not visible during the session since participants do not bring the cash (Feature 2). The idea was to make the endowment as intangible as possible on the session day. This aspect of the design was inspired by the evidence that the less tangible a payment is, the more people are willing to pay (*82*), which points to the existence of some kind of "money blindness" in people when the money is intangible (i.e., when it is not visible, when it was received a long time ago, etc.). In the same logic (making the endowment as intangible as possible on the session day), the participants were told in the task instructions that they would have to pay off the amount corresponding to their losses up to \$95; the endowment was not mentioned (the text of the task instructions can be found at https://tinyurl.com/y5fmb4ns).

Power analyses (experimental data)

For the test comparing the prevalence rate of the picking pennies bias when the participants were instructed about the bowman type and when they were not, power to detect a medium effect size (*Cramer's V*) with a 5% significance level and given our sample size in this test (N=168) is 99.8%. (For reference, standard values used to define "small", "medium" and "large" V are 0.1, 0.3, and 0.5.) Table S15 shows that the minimum effect size needed to detect an effect with satisfactory (at least 80%) power is in the 0.15, 0.19 range. The observed effect size documented in the main text (0.05) is way below this minimum value, even for a significance level of 10%. This suggests that the reported absence of evidence in this test is not due to the test being underpowered but to the effect being either absent or minute.

For the test comparing the accuracy of the penny-pickers to the accuracy of the other task participants (pooling the data from Experiment 3 and Experiment 5), power to detect an effect (*Cohen's D*) with a 5% significance level and given our sample size in this test (N=99) is 64.8% for a medium effect size, and 96.3% for a large effect size. (For reference, standard values used to define "small", "medium" and "large" effect sizes are 0.2, 0.5, and 0.8.) Table S16 shows that the minimum effect size needed to detect an effect with 90% power and a significance level of 5% is 0.69. The observed effect size documented in the main text is 0.99.

For the test comparing the prevalence rate of the picking pennies bias when a commitment device was offered to task participants and when no device was offered, power to detect a medium effect size with a 5% significance level and given our sample size in this test (N=184) is 99.6%. Table S17 shows that the minimum effect size needed to detect an effect with 80% power and a significance level of 5% is 0.18. The observed effect size documented in the main text is 0.19.

For the test comparing the prevalence rate of the picking pennies bias when the task participants were exposed to real monetary losses and when they were not, power to detect a medium effect size with a 5% significance level and given our sample size in this test (N=223) is 99.4%. Table S18 shows that the minimum effect size needed to detect an effect with 80% power is 0.18 (for a significance level of 5%) and 0.16 (for a significance level of 10%). The observed effect size documented in the main text (0.03) is way below this minimum value.

For the test comparing the fit of the CbD and base models, power to detect a medium effect size (*Cohen's D*) with a 5% significance level and given our sample size in this test ($N = 120$) is 100%. Table S19 shows that the minimum effect size needed to detect an effect with 90% power and a significance level of 5% is 0.29. The observed effect size documented in the main text is 0.86.

Supplementary model comparison analyses (experimental data)

We ran an extensive set of robustness checks for the model comparison analysis reported in the article. We re-ran the model comparison under the assumption of risk neutrality (α_1, α_2, α_3 and λ are set to 1) and, in a separate analysis, we allowed for loss tolerance ($\lambda \geqslant 0$ vs. $\lambda \geqslant 1$ in the main analysis). Whatever the constraints imposed on the risk preference parameters, the conclusion is always that the CbD model fits the penny-pickers' behavior significantly better than the base model does. We also checked that the conclusion holds across different model specifications. We found that the evidence for the CbD model is strengthened in the setting where α_1 and α_2 are constrained to be equal. We also ran the model comparison with a simplified version of the CbD model in which the Pavlovian factor (DA) is a fixed free parameter (see "Alternative specifications of the CbD model" above). We found that the simplified CbD model underperforms the CbD model presented in the main text, but the main conclusion still holds. Finally, we re-ran the above analyses under the assumption that participant choice is deterministic, i.e., the task participant chooses to bet with probability 1 if $U(L) > 0$, and to skip otherwise. In this case, we minimized the free parameters of the models by minimizing the total squared prediction error compounded

over the set of trials for the first eight sessions. We measured goodness of fit by the percentage of correct choice predictions in the last seven sessions. All the above results are qualitatively unchanged in that setting.

Supplementary model comparison analyses (simulated data)

To assess the validity of the estimation procedure used in the model comparison analysis, we simulated the CbD model in N runs of the learning version of the experimental task, setting each model parameter to the average fitted value obtained across task participants in these experiments ($\hat{\theta}$= 0.9; $\hat{\kappa}_1$ = 0.4; $\hat{\kappa}_2$ = 0.6; $\hat{\beta}$ = 1.1, $\hat{\alpha}_1$ = 0.7, $\hat{\alpha}_2$ = 0.8, $\hat{\alpha}_3$ = 0.8, $\hat{\lambda}$ = 1.1). We then fitted the CbD model to behavior in each of the N simulated runs, and the prediction accuracy of the model was then assessed, using the procedure described above. Similarly, we fitted the base model and assessed its accuracy to predict behavior in each of the N simulated runs. We set N to 120 (the sample size in the main test comparing the goodness of fit of the CbD and base models). Fig. S11 A shows that the CbD model outperforms the base model in the large majority (91%) of the simulated runs. We re-did the same procedure, this time simulating 120 times the base model, and then fitting the CbD and base models to behavior in each of the 120 simulated runs. This time the base model outperforms the CbD model for most of the simulated subjects (Fig. S11 B). We also looked at the distribution of the fitted parameter value across simulated runs for the parameters of the craving function. We found that the mode of the fitted value matches the true value, but variance is quite large (so we caution against using the participant-specific fitted value in the current paradigm; the average value across participants shall be more reliable).

Supplementary analysis strengthening the evidence for a selling pressure on cheap calls price (financial data)

We sorted options into delta and volatility baskets and compare the returns of the cheap calls to the returns of the other calls. Strikingly, cheap call selling consistently delivers EV<0 even without incorporating transaction costs: the MID returns of the cheap calls are indeed positive across *all* volatility levels except for the top level (Table S16, Panel A), and across *all* delta levels, in contrast to that with the other calls, for which the MID returns are negative across all delta levels (Table S16, Panel B). [Note: A fortiori, the SELL returns are negative across all delta levels for the cheap calls, and positive for the other calls.]

The finding that the MID returns of the cheap calls are positive across all volatility levels, except for the top level, is consistent with the idea of a selling pressure that consistently dominates the countervailing buying pressure coming from the volatility premium/demand-for-insurance. The finding that the MID returns are positive across all delta levels suggests that this selling pressure also dominates the countervailing buying pressure coming from the gambling motive. The delta of a given option is indeed a proxy for the degree of attractiveness of the option both on the demand side (lower delta options have lower cost and higher potential returns if the option finishes in the money) and on the supply side, having in mind the craving motive (lower delta options have lower probability of finishing in the money and higher average median returns: 90.3% on average for the options in two lowest delta deciles vs 0.05% for the two highest delta deciles).

Supplementary analysis suggesting an increased buying pressure for the cheap calls relative to the other calls (financial data)

In the same way that we computed the difference between SELL and MID returns to measure the selling pressure for an asset, we computed the difference between BUY and MID returns, which can be used as a measure of the average buying pressure. The difference is −14.09% for the cheap calls vs. −6.31% for the other calls (Table S6), pointing to a *higher* buying pressure for the cheap calls, which would be consistent with the "good deal effect" documented in financial economics (*83*)—and it could also reflect a higher gambling motive for the buyers of the cheap calls. The evidence of an increased buying pressure on the cheap calls rules out the possibility that the negative expected value from cheap call selling reflects a depressed demand for these calls.

Supplementary fixed effects regressions (financial data)

We ran the main fixed effects regressions (Table S11) without {MP<1} included and on all options pooled (in reference to the existing literature), the cheap calls, and the other calls, in three separate regressions (Table S17). The results for all options fully replicate the existing body of knowledge; those for the cheap calls and the other calls fully confirm that the cheap call selling anomaly—the positive MID returns and negative SELL returns of the cheap calls—is distinctive to the cheap calls. Moreover, and importantly, we replicated all the foregoing findings in regressions of our full model (Table S11, regression (3)) augmented with option specific

characteristics directly tied to the volatility premium and unhedgeable risk—the so-called *"Greeks"* (Table S18).

Tests of the idea that cheap call selling reflects a rational motive in the sellers (financial data)

Hedging. The ISE data fully confirm the existence of the cheap call selling anomaly: the MID returns (resp. SELL returns) for open sell transactions are positive (resp. negative) for the cheap calls and negative (resp. positive) for all other options, see Table S19. This finding rules out the possibility that the cheap call selling anomaly reflects trading by market makers who engage in cheap call selling and offset the risk through dynamic replication and delta/vega hedging (using the underlying instrument and other options). The cheap call selling anomaly *cannot* reflect this as we did remove market makers from the ISE data for all the current analyses. As for non market makers, the cost of dynamic replication and delta hedging cheap calls is excessively costly for them, due to the bid-ask spreads and the gamma risk from a move when the option goes into the money. (To be more specific, the daily cost of delta rebalancing far exceeds the benefit of selling a cheap call: average ratio of delta to price for the cheap calls: 0.48; for all other options: 0.01.) We conclude, therefore, that the cheap call selling anomaly is unlikely to reflect hedging.

Liquidity motive. We also considered the possibility that the anomaly comes from firms that are providing liquidity across a range of options and are willing to give up some returns from selling the cheap calls to gain volume in other options. Such liquidity motive may well exist, but it is not consistent with several of the current findings. For example, it fails to explain why the size of the anomaly correlates with the degree of craving power of the asset the way it does (Fig. S8), and why the anomaly concerns not only firms but customers as well (Table S13).

Misaligned incentives. It is well known that the investment management industry rewards high Sharpe ratio managers, and the Sharpe ratio can be manipulated with option-like strategies. We asked, therefore, whether cheap call selling could reflect an attempt to achieve higher Sharpe ratios. The results documented by Goetzmann et al. (2002) suggest that the answer is negative *(84)*. The evidence indeed shows that selling calls, combined with selling puts, can achieve a Sharpe ratio maximizing portfolio beyond what is achievable with a standard risk-free plus risky asset portfolio. But the strategy only works with calls closer to at-the-money, relative to the cheap calls.

With out-of-the-money calls, the strategy fails. We conclude, therefore, that there is little reason to believe that cheap call selling reflects the incentive to achieve higher Sharpe Ratios.

Replication of the main experimental findings in an independent sample

The experimental findings were fully replicated in an independent sample of 77 participants at Brown University. The lab conditions replicated those of Experiment 3 in all aspects. However, the setting was arguably quite different in several regards, which includes the fact that participants differed in terms of nationality and degree of sophistication (arguably—Brown University is one of the top universities in the US), and the sessions were run by different experimenters (two research assistants from Brown University collected the data; none of the authors of the current study were involved in the data collection at Brown University). The picking pennies bias presents itself consistently in that study as well (Table S20).

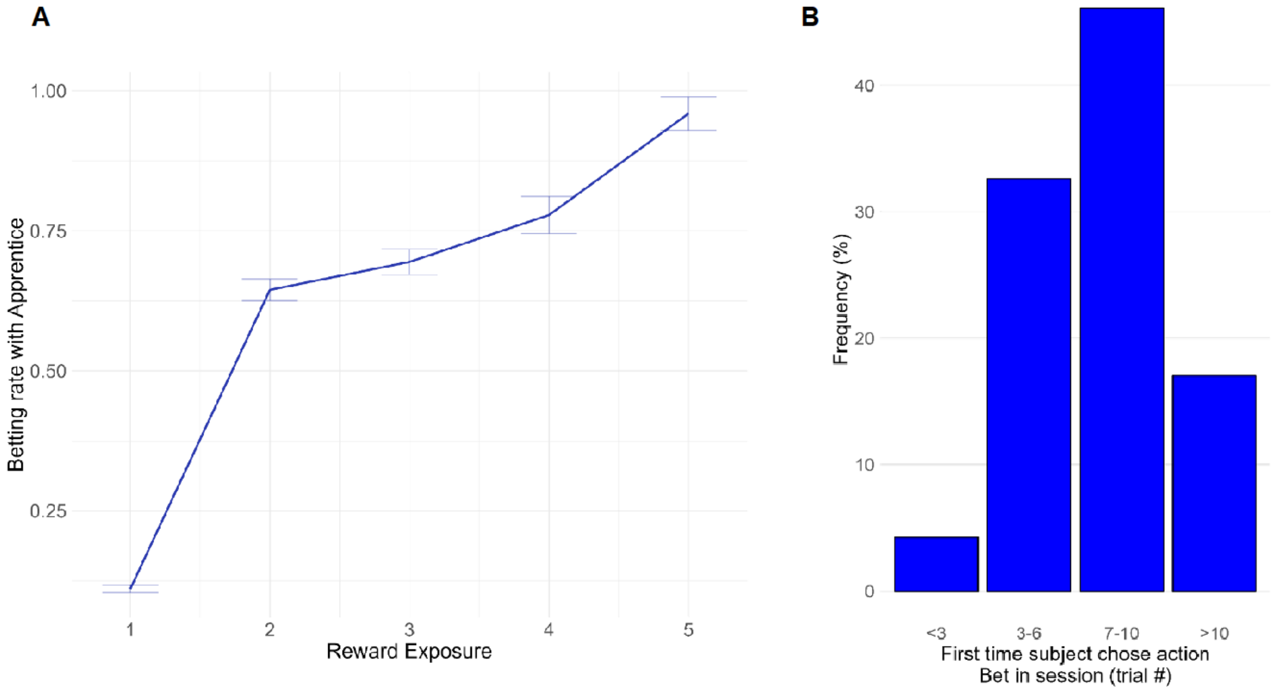

Fig. S1. Dynamics of the picking pennies bias. (A) The probability of the bias increases with reward exposure. X-axis: reward exposure (frequency of winning bets in previous trials). The variable was split into 5 bins of equal range. Error bars show standard errors of the mean (SEM). Y-axis: betting rate in the sessions with an apprentice computed across all sessions and task participants. **(B)** The graph reports the first time the penny-pickers started to bet in a session with an apprentice on average, at the participant level. For both graphs, the statistics were computed for the learning version of the task, pooling data from Experiments 1, 3, 4, and 5. The results are qualitatively the same for each experiment studied separately.

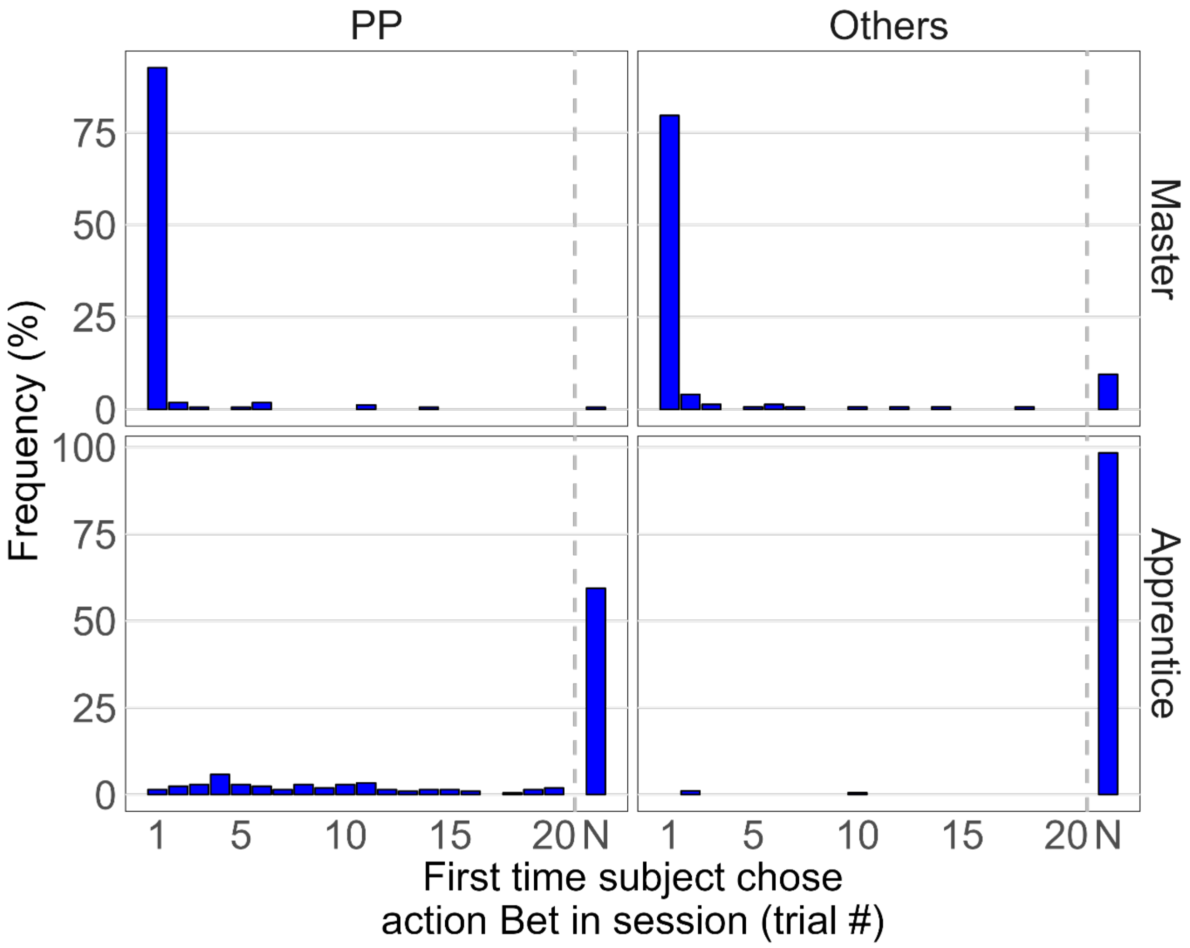

Fig. S2. Distribution of the first time the participants bet within the sessions in the “no ambiguity” version of the task (Experiment 2). The distribution was derived across all participants and sessions of Experiment 2, per session type (sessions with a master vs. sessions with an apprentice) and participant type (penny-pickers: “PP”; other participants: “Others”). N: sessions in which the participants chose to skip in all trials of the session.

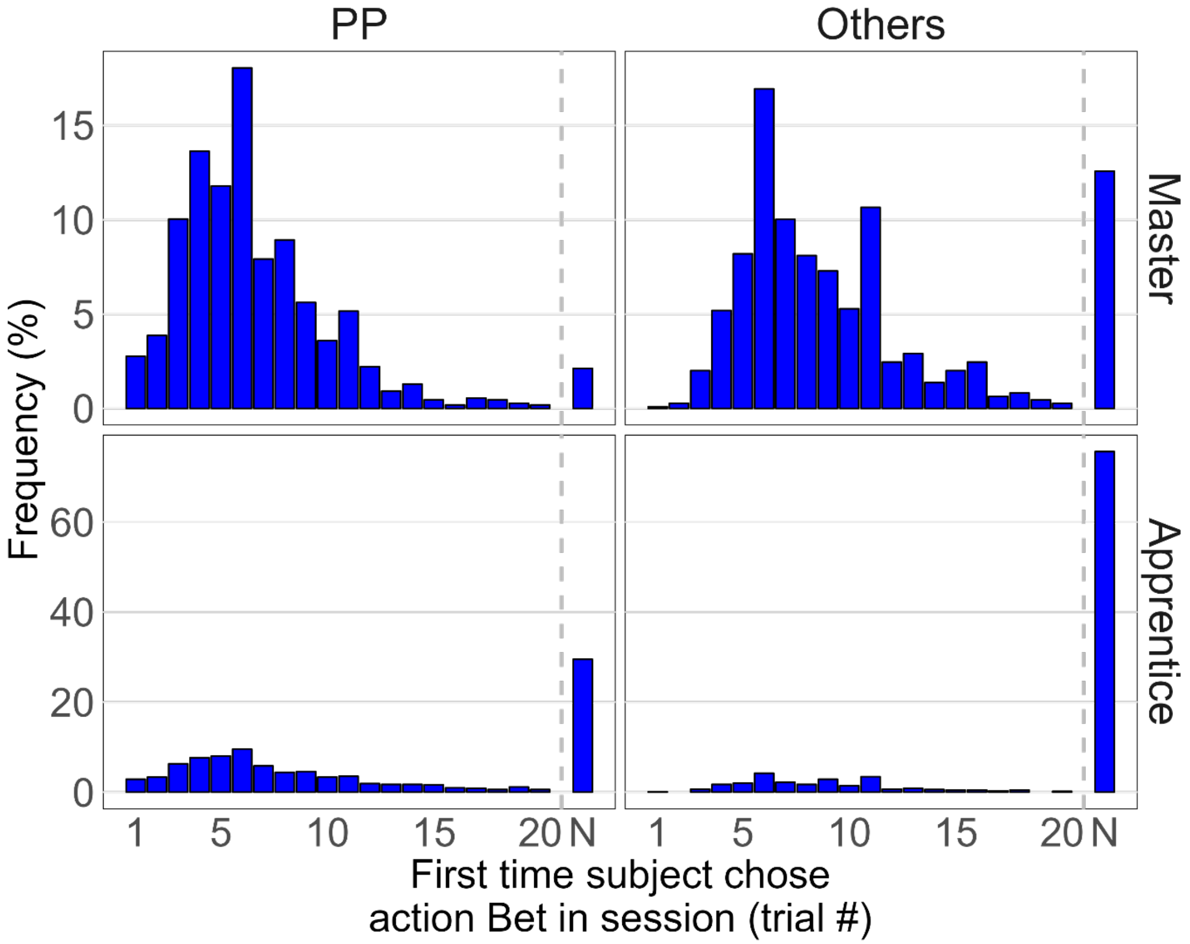

Fig. S3. Distribution of the first time the participants bet within the sessions in the learning version of the task. The distribution was derived across all participants and sessions of Experiment 1, 3 and 5 (pooled here; the results are qualitatively the same for each experiment studied separately), per session type (sessions with a master vs. sessions with an apprentice) and participant type (penny-pickers: “PP”; other participants: “Others”). N: sessions in which the participants chose to skip in all trials of the session.

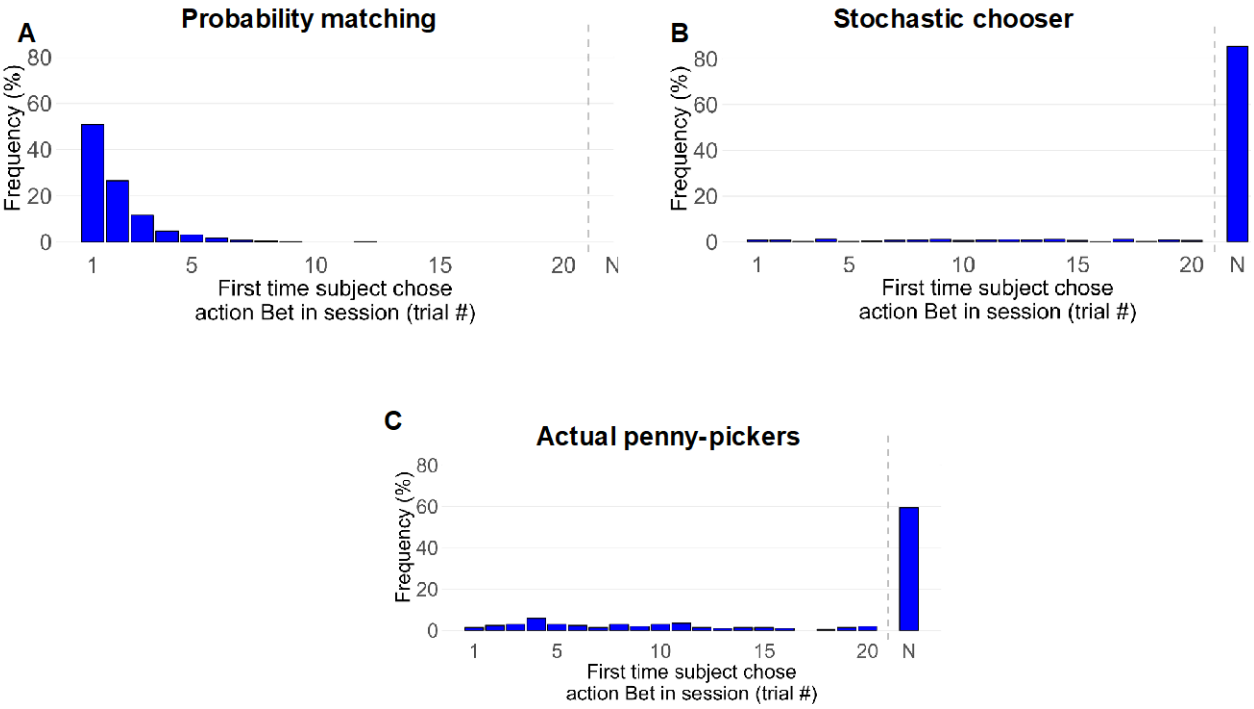

Fig. S4. Distribution of the first time the participants bet within the sessions with an apprentice. (A) The distribution was derived across 100 simulated runs with an agent that follows the probability matching rule to make a decision on each trial. In each simulated run, on each trial, the agent bets 80% of the time (which "matches" the probability of a winning bet in the sessions with an apprentice). **(B)** The distribution was derived across 100 simulated runs with a stochastic chooser (the base agent described by Eq. (1) and (2) in the article) with β =0.7, which corresponds to the maximum degree of choice randomness inferred in our sample (based on the fitted parameter values for β). Under such a value, a stochastic chooser bets with probability 0.01 on each trial in the sessions with an apprentice. **(C)** The distribution was derived across all penny-pickers and sessions with an apprentice in Experiment 2 (the graph is the same as the bottom left graph in Fig. S2). N: sessions in which the participants chose to skip in all trials of the session.

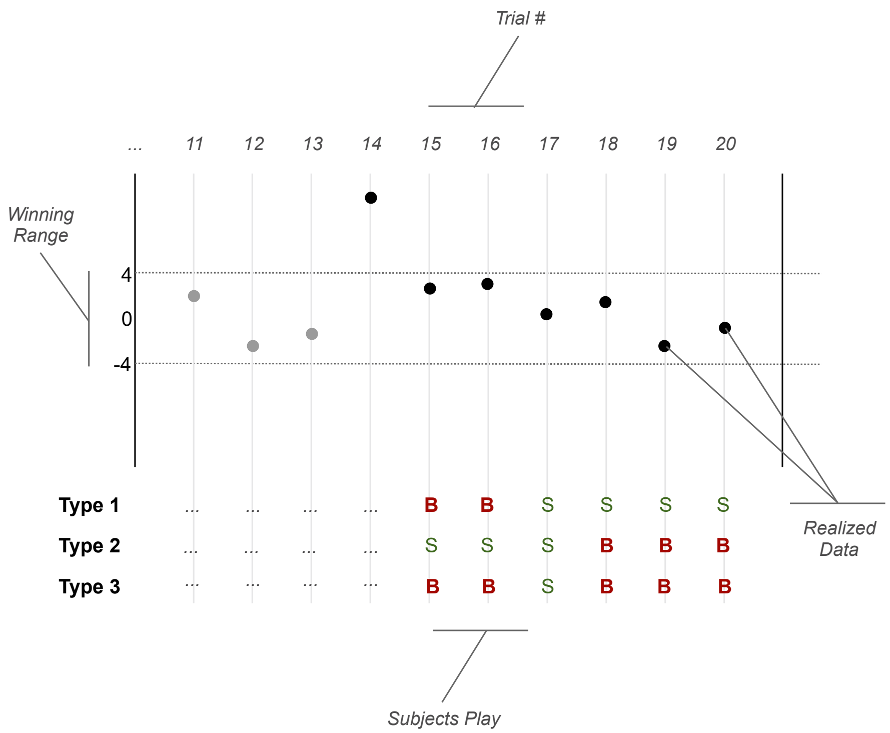

Fig. S5. Description of the picking pennies bias using an exemplar session. In this example, a "black swan" (as defined in the main text) occurs in trial 14 of the session, and behavior is described from that trial on. Type 1 (29.4% of the penny-pickers): participant bets in either of the two trials following the black swan and the overall betting rate thereafter is less than 50%. Type 2 (60.9%): participant does not bet in the two trials directly following the black swan but they bet sometime later in the session (N.B., about 35% of them bet before the fifth trial following the black swan). Else, if the overall betting rate in the trials following a black swan is more than 80%, the session is labelled Type 3 (9.7%). Else, the session is labelled "Others" (1.8%).

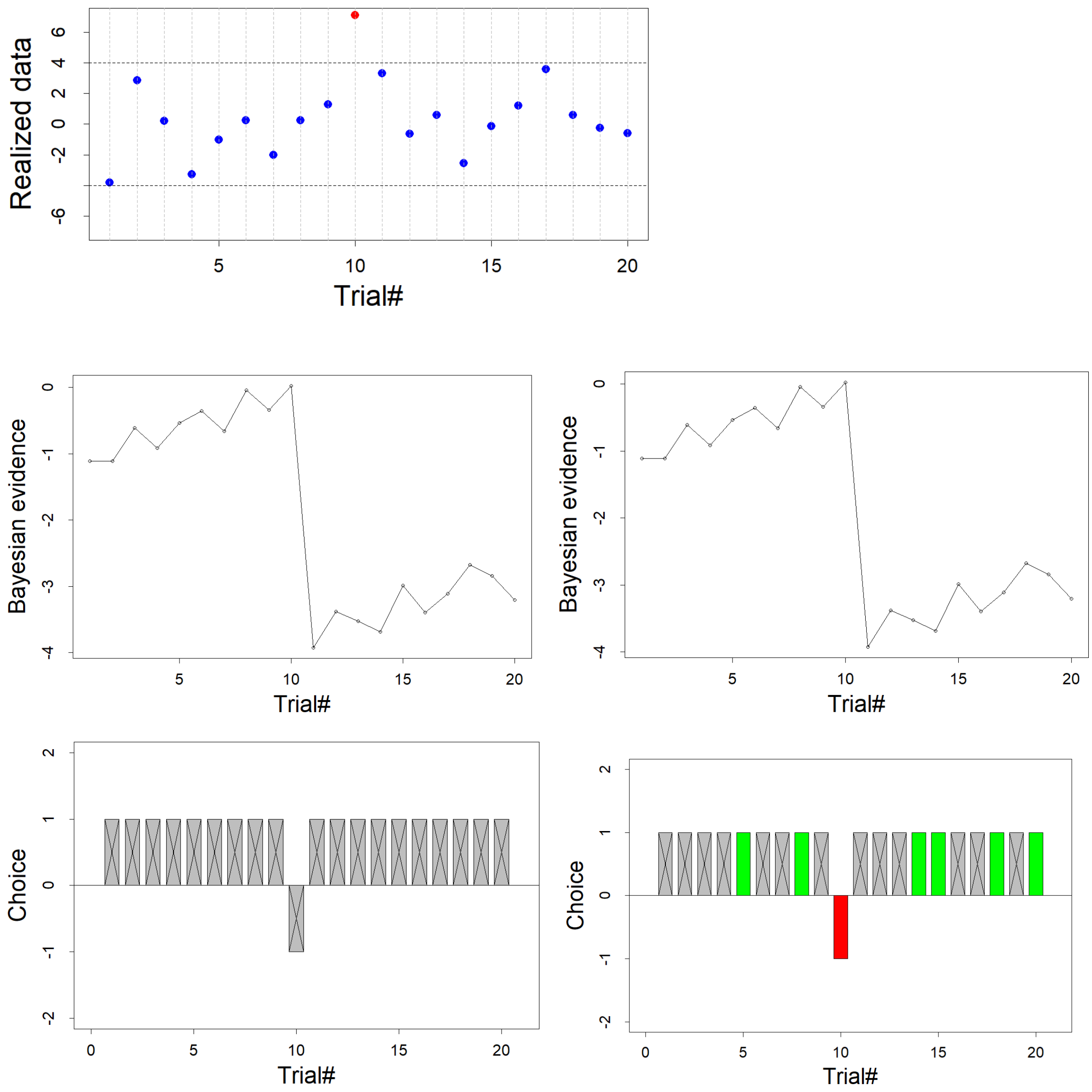

Fig. S6. Simulated run of a session with an apprentice bowman. (A) Behavior of the base model. **(B)** Behavior of the CbD model. The top graph indicates the shot realized at each trial of the simulated run. Here, a shot falling more than seven meters away from the target ("black-swan") occurs at Trial 10. The other shots fall within the winning range. The middle graph reports the Bayesian evidence that the session is with a master bowman (computationally, it is defined as log $\left(m_1\left(\underline{X_1}\right)/m_2\left(\underline{X_1}\right)\right)$, see Eqs. (S6) and (S7) in Supplementary Text). The bottom graph shows the choice predicted by the model in each trial. A crossed grey box indicates a foregone gain at the corresponding trial (the agent chooses to skip and the shot falls within the winning range). A red box indicates a realized loss (the agent chooses to bet and the shot falls outside the winning range). A green box indicates a realized gain (the agent chooses to bet and the shot falls within the winning range). Model parameters were set to the average fitted values obtained across task participants (see Supplementary Text). The base model predicts that the agent skips throughout the session. Under the CbD model, the agent starts betting at Trial 5, skips in the three trials following the black swan, and bets with positive probability thereafter, which corresponds to Type 2 in Fig S5

(observed in about 80% of the 2,000 simulated runs; in about 20% of the runs, the agent chooses to bet immediately in one of the two trials after the black swan with an overall betting rate below 50% in following trials, see Type 1 in Fig S5).

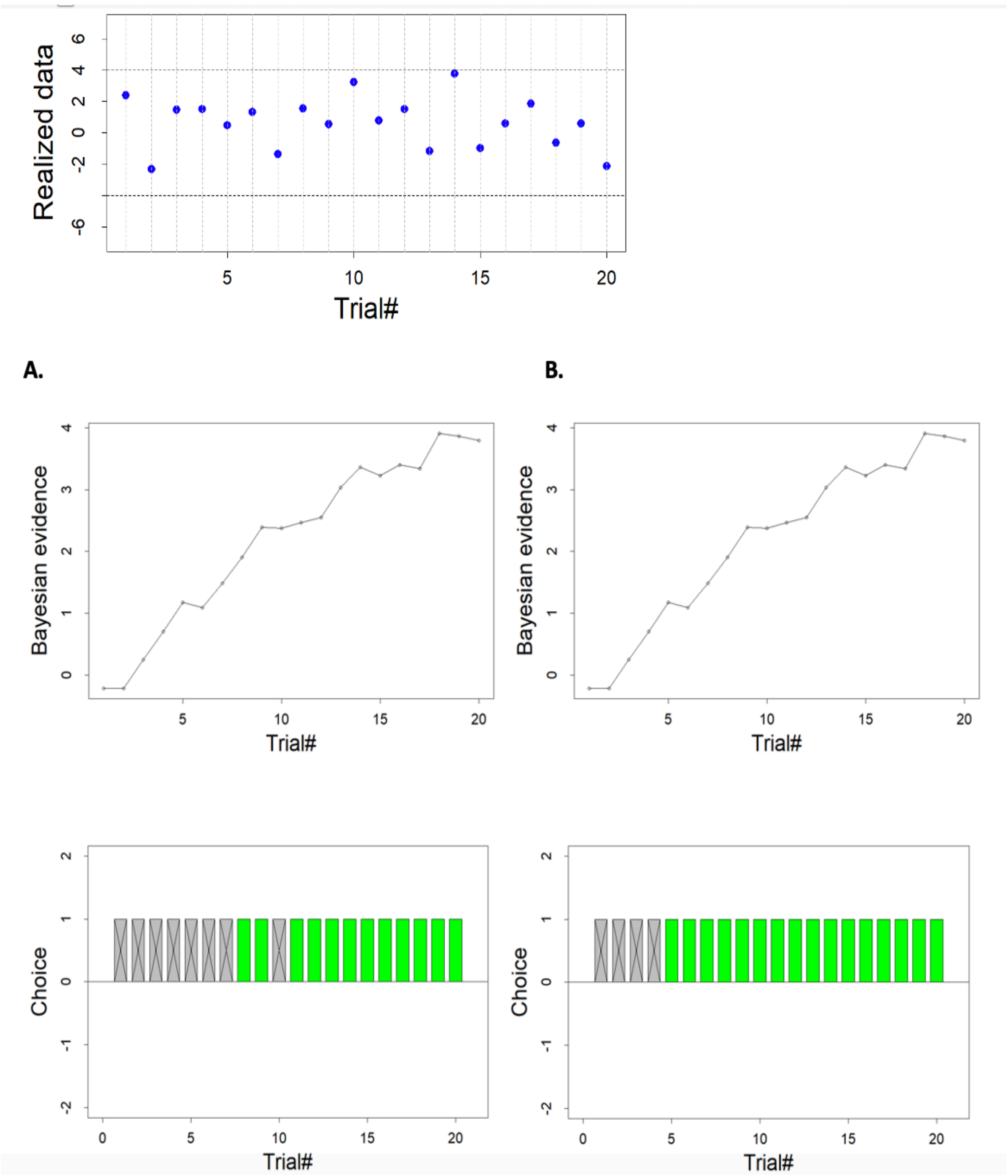

Fig. S7. Simulated run of a session with a master bowman. (A) Behavior of the base model. **(B)** Behavior of the CbD model. See Fig. S6 for the legend. In this simulated run, under the base model, the agent starts betting at Trial 8. The CbD agent starts betting earlier and the overall betting rate is higher, which "matches" the pattern observed in the penny pickers (see *"Penny-pickers bet more than the other participants in sessions with a master bowman"* in the main text).

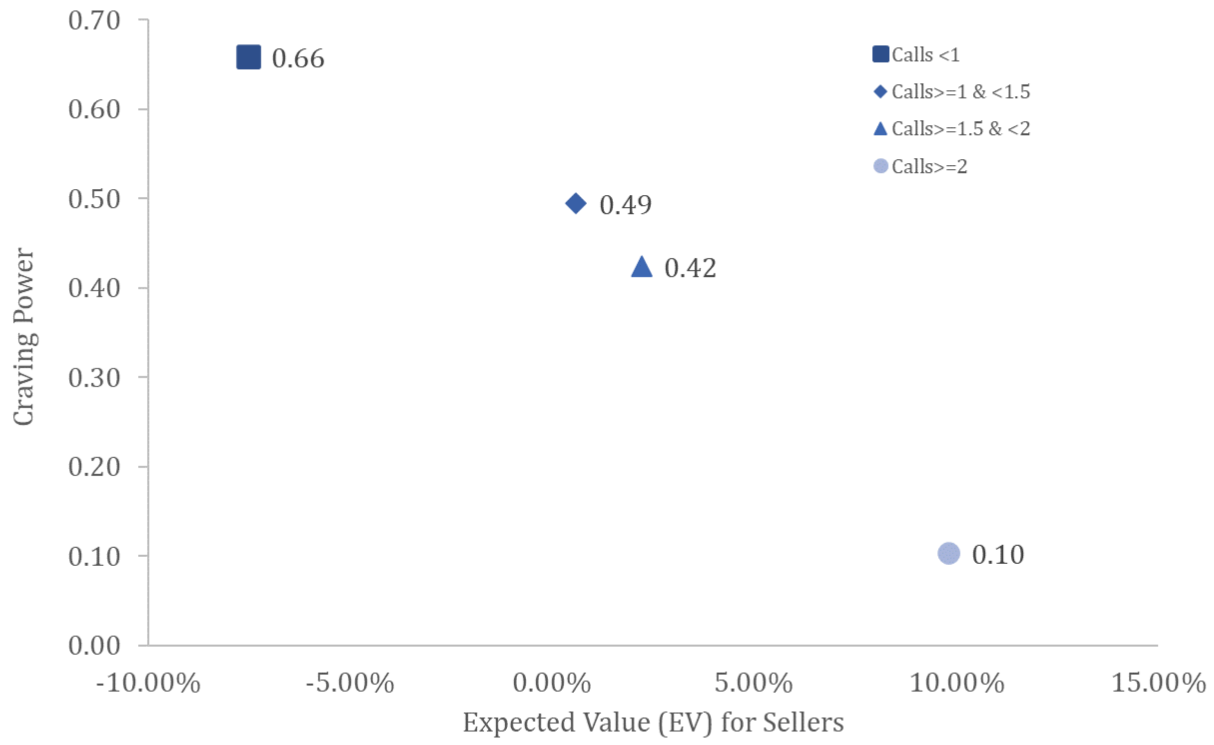

Fig. S8. Relationship between the expected value from selling a given call category and the degree of craving power of selling this type of calls. X-axis: Expected value (EV) from selling the option. Y-axis: Degree of craving power, measured by the median returns from selling the option × the percentage of the options finishing out of the money.

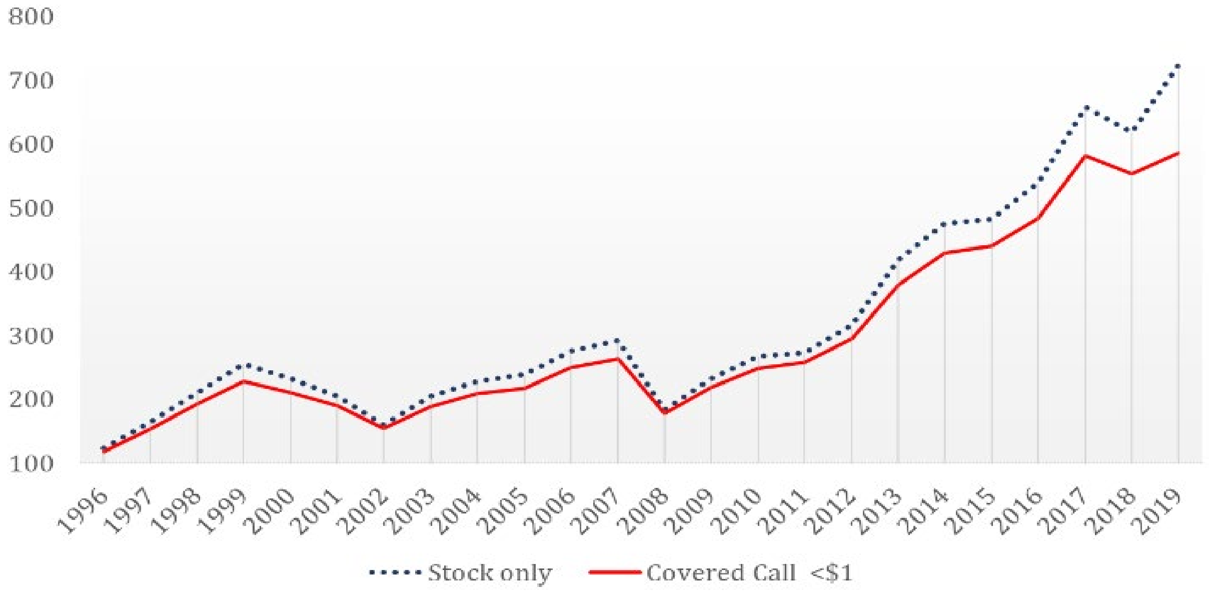

Panel A: Calls < $1

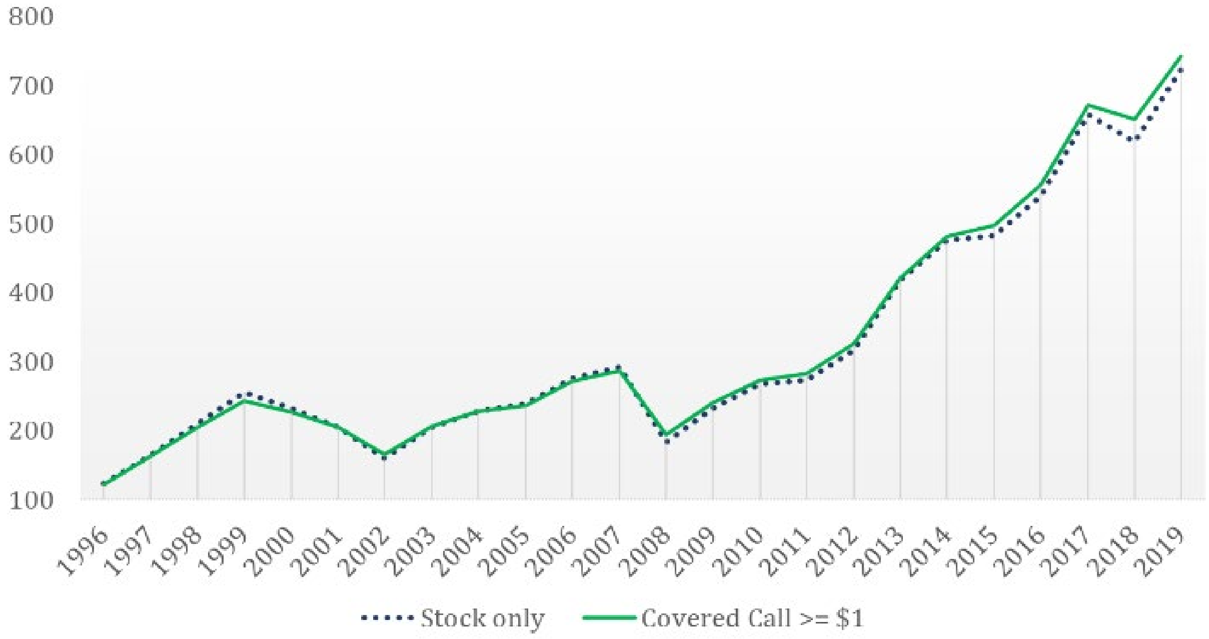

Panel B: Calls ≥ $1

Fig. S9. Performance of covered call strategy based on cheap call selling (Panel A) vs. selling the other calls (Panel B) for the period 1996-June 2019. Plain curve: Performance (cumulative returns) of covered call strategy. Dashed curve: performance of simply being long the underlying stock. The gap between the two curves reflects the performance of selling calls over that period. The cumulative returns are normalized so that the portfolio starts at a value of 100 on January 1996. The figure clearly shows that cheap call selling has consistently yielded negative value over the period (dashed curve is above the plain curve), whereas the opposite is true for the other calls.

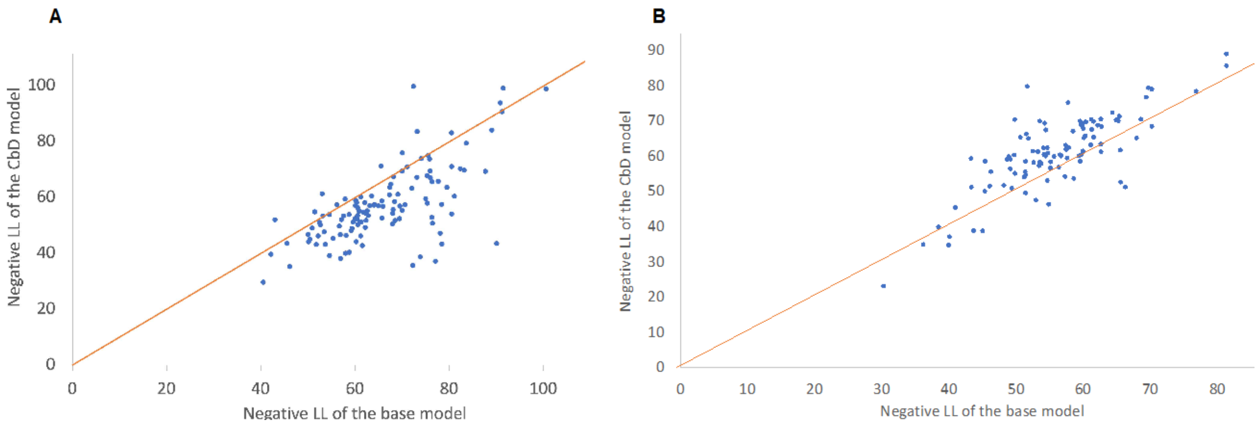

Fig. S10. Comparative goodness of fit of the base and CbD models for the penny-pickers (A) and the other task participants (B). Each data point corresponds to one participant. For each participant, the graph reports the out-of-sample LL of the base model (X-axis) and the CbD model (Y-axis). The CbD model fits better when the data point is below the 45-degree line.

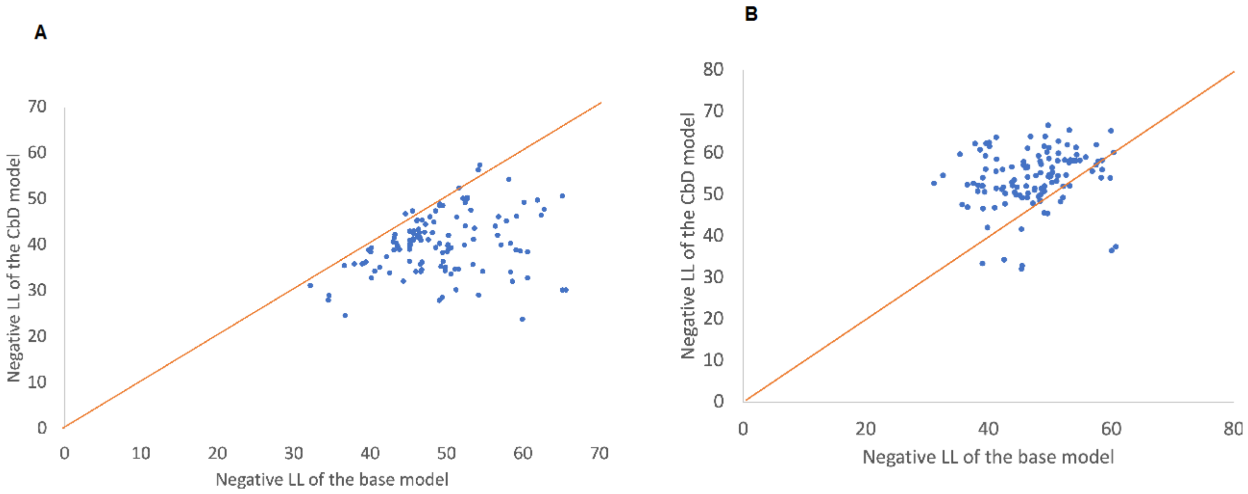

Fig. S11. Model recovery for the estimation procedure used in the model comparison analysis. Comparative goodness of fit (out-of-sample LL) of the base model (X-axis) and the CbD model (Y-axis) for simulated CbD agents (A) and for simulated base agents (B). Each data point corresponds to one simulated agent. The CbD model fits better when the data point is below the 45-degree line.

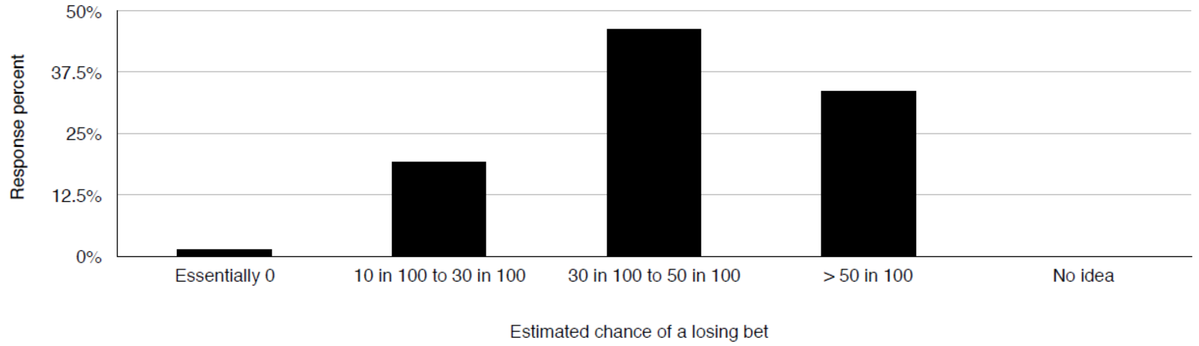

Fig. S12. Participant estimates of the probability of a losing bet in a session with an apprentice in Experiments 1-5 The graph shows the distribution of the estimated probability of a losing bet with an apprentice bowman reported by the participants in a debriefing questionnaire filled out at the end of the experimental session. X-axis: Estimated chance of a losing bet in a session with an apprentice. Y-axis: Response percent across participants. The mode estimate across participants is in the 30%-50% range; the true statistic is approximately 20%.

	N	Mean	SD	Median	Min	Max	Skew	Missed
**Exp 2**	14079	0.56	0.34	0.47	0.07	4.59	3.03	1
**Exp 3**	16259	0.62	0.43	0.49	0.07	4.94	2.92	1
**Exp 5**	14099	0.71	0.46	0.57	0.05	3.75	2.39	1
**Total**	44437	0.63	0.42	0.51	0.05	4.94	2.77	3

Table S1. Summary statistics for reaction time (seconds) and missed trials in the experiments run for the study. Note that reaction time data are not available for Experiments 1 and 4 due to a mistake in the code.

	All black swan events			Only memorable events		
	7m	8m	9m	7m	8m	9m
**Exp 1**	59.7	53.2	52.4	43.5	37.1	37.1
**Exp 2**	52.3	52.3	52.3	52.3	52.3	52.3
**Exp 3**	44.6	44.6	44.6	38.7	36.8	36.8
**Exp 4**	33.3	30	30	21.7	18.3	18.3
**Exp 5**	51.1	48.9	48.9	34	31.9	31.9

Table S2. Percentage of penny-pickers in each experiment for different criteria used to measure the picking pennies bias. The criteria vary in the threshold value used to define a black swan (distance from target: 7 meters; 8 meters; 9 meters), and in whether all the instances of penny picking are included in the analysis ("All black swan events"), or only the instances when participants lose $40 as a consequence of betting ("Only memorable black swan events").

	7m		8m		9m	
	**PP**	**Others**	**PP**	**Others**	**PP**	**Others**
**Exp 1**	12.20	12.01	10.39	10.37	9.50	9.67
**Exp 2**	15.40	15.71	13.70	14.32	11.44	12.37
**Exp 3**	13.10	14.72	11.37	13.39	10.68	11.76
**Exp 4**	13.00	12.51	11.82	11.04	9.45	9.14
**Exp 5**	12.94	14.45	11.00	13.13	9.93	11.34
**Total**	12.84	13.47	11.15	11.94	9.96	10.51

Table S3. Average number of black swans seen by the penny-pickers ("PP") and the other participants ("Others"). The average is computed for each experiment under the different threshold used to define a black swan in the experimental task (a shot that falls more than 7m/8m/9m away from target).

A

	Trial when pp started
Mean	8.61
SD	3.80
Median	7.25
Mode	4.67/7.25

B

	7m	8m	9m
	Mean trial when pp started		
Experiment 1	13.4	13.5	13.7
Experiment 3	12.4	12.2	12.3
Experiment 5	11.6	11.6	11.7
Total Mean	12.9	12.8	13.0
Total SD	2.83	2.93	2.99
Total Median	13	13	13
Total Mode	11	13	13

Table S4. When the picking pennies behavior started within a session. (A) Trial when the picking pennies behavior started (recall a session comprises 20 trials overall), averaged across the sessions in which the participants picked pennies in Experiment 2. **(B)** Trial when the picking pennies behavior started averaged across the sessions in which the participants picked pennies in the learning version of the task (Experiments 1, 3, and 5). The standard deviation (SD), median, and mode are also reported.

	7m				8-9m			
	Master		Apprentice		Master		Apprentice	
	PP	Other	PP	Other	PP	Other	PP	Other
Exp 1	66.4%	56.6%	27.4%	9.7%	66.1%	57.8%	28.1%	11.1%
Exp 2	91.7%	80%	6.2%	0%	91.7%	80%	6.2%	0%
Exp 3	77.7%	47.4%	42.3%	6.1%	78.1%	48.1%	43.2%	6.7%
Exp 4	65%	54.3%	23.7%	7.2%	66.3%	54.4%	25.7%	7.4%
Exp 5	67.6%	45.7%	26.4%	7%	67.9%	46.3%	27.2%	7.3%
Total	72.8%	55.3%	25.4%	7.1%	73.5%	56%	25.9%	7.9%

Table S5. Betting rates in each experiment per session type and participant type. Participants were classified (penny-pickers: "PP"; other participants: "Others") based on the different criteria we used to define penny picking. The statistics turn out to be the same for the 8m and 9m criteria so we merged them here.

		All Options			Calls < $1			Calls ≥ $1		
		MID	BUY	SELL	MID	BUY	SELL	MID	BUY	SELL
Average	%	-0.088	-0.169	0.005	0.074	-0.067	-0.238	-0.007	-0.077	0.071
	$	-0.967	-1.209	0.724	0.042	-0.035	-0.118	-0.756	-1.060	0.452
Median	%	-0.684	-0.734	0.633	-0.947	-1.000	0.886	-0.494	-0.547	0.442
	$	-1.014	-1.166	0.868	-0.354	-0.406	0.300	-1.090	-1.285	0.904
SD	%	1.777	1.661	1.917	3.244	2.981	3.566	1.582	1.494	1.686
	$	9.120	9.134	9.113	1.640	1.610	1.675	11.494	11.500	11.495
Skew	%	7.371	7.318	-7.431	13.107	12.931	-13.325	6.583	6.546	-6.639
	$	0.497	0.430	-0.552	9.913	10.151	-9.636	-0.365	-0.527	0.206
OTM (in %)			0.509			0.704			0.402	
Unique N			17,071,406			5,256,144			7,124,416	
N			183,983,273			22,121,539			79,203,260	

Table S6. Summary statistics of option returns The table shows the MID, BUY, and SELL returns for all options, cheap calls ("Calls < $1"), and the other calls ("Calls ≥ $1"). Both percent returns and dollar returns are shown (to highlight that the percent returns are not driven by outliers in the current sample). The table also reports median returns (Median), standard deviation (SD), the proportion of options that expire out-of-the-money (OTM), which is when the call sellers win money, the number of unique options in the sample (Unique N), and the total number of options in the sample (N).

	Dependent Variable: participant type		
	7m	8m	9m
Intercept	0.357	0.361	0.333
	(0.479)	(0.474)	(0.500)
Experiment 2	-0.192	0.216	-0.003
	(0.469)	(0.476)	(0.474)
Experiment 3	0.195	0.448	0.423
	(0.381)	(0.386)	(0.384)
Experiment 4	-0.864**	-0.758*	-0.833**
	(0.379)	(0.394)	(0.395)
Experiment 5	0.036	0.300	0.239
	(0.407)	(0.413)	(0.406)
Blackswan number	-0.023	-0.058	-0.059
	(0.036)	(0.042)	(0.048)
First bet (Late)	-0.955*	-0.707	-0.710
	(0.492)	(0.493)	(0.494)
First bet (Never)	-3.067***	-2.907***	-2.857***
	(0.62)	(0.564)	(0.564)
McFadden R^2	0.18	0.16	0.16
N	327	327	327

Table S7. Logistic regression predicting the picking pennies bias. The dependent variable is a dummy for participant type (0: Non penny-picker; 1: Penny-picker). The independent variables are predictors for Experiment [dummy to compare Experiment 1 (the reference) to Experiments 2-5], the (z-scored) total number of black swans seen by the participant ("Black swan number"), when the first bet decision most commonly occurred in the session [dummy coded "Early" = first 10 trials (reference), and "Late" = last 10 trials], and whether betting never occurred ("Never"). Here penny picking means betting after a memorable black swan as defined in the main text (the results are qualitatively unchanged when considering all black swan events). We run three regressions, each using a different threshold value to define a black swan event, as discussed in the main text (7 meters, 8 meters, and 9 meters from target). Due to small group sizes, we use penalized loglikelihood estimation (*85*). * < .10, ** < .05, *** < .01.

	7m	8m	9m
Intercept	6.46 ***	5.77**	5.55**
	(2.45)	(2.34)	(2.37)
Experiment 5	0.16	0.11	0.056
	(0.56)	(.56)	(.55)
Black swan number	-0.09	-0.16**	-0.10
	(0.07)	(0.08)	(0.09)
First bet (Late)	-2.40	-2.15	-2.32
	(1.65)	(1.64)	(1.64)
First bet (Never)	-4.06***	-4.061***	-3.91***
	(1.43)	(1.440)	(1.43)
Accuracy	-5.87**	-4.270	-5.09*
	(2.86)	(2.782)	(2.72)
McFadden R^2	0.42	0.41	0.38

Table S8. Logistic regression predicting the picking pennies bias in Experiments 3 and 5. The model uses as dependent variable participant type (0: Non penny picker; 1: Penny Picker). The independent variables include predictors for Experiment [dummy to compare Experiment 5 (the reference) to Experiment 3], the total number of black swans seen by the participant ("Black swan number"), when the first bet decision most commonly occurs in the session [dummy coded ``Early'' = first 10 trials (ref), and "Late" = last 10 trials], whether betting never occurs ("Never"), and the proportion of sessions in which the participant successfully guesses the bowman type ("Accuracy"). The continuous variables are z-scored. Here penny picking means betting after a memorable black swan as defined in the main text (the results are qualitatively unchanged when considering all black swan events). We run three regressions, each using a different threshold value to define a black swan event, as discussed in the main text (7 meters, 8 meters, and 9 meters from target). Due to small group sizes (e.g. few penny pickers never bet), penalized log-likelihood estimation is used (*85*). * < .10, ** < .05, *** < .01.

	Dependent Variable: betting after a black swan	
	7m	8-9m
Intercept	-1.278***<br>(0.168)	-1.029***<br>(0.179)
Experiment 2	-0.364<br>(0.327)	-0.412<br>(0.328)
Experiment 3	0.218<br>(0.218)	0.148<br>(0.225)
Experiment 4	0.563**<br>(0.276)	0.605**<br>(0.296)
Experiment 5	-0.145<br>(0.241)	-0.195<br>(0.252)
First bet (Late)	-0.074<br>(0.116)	-0.153<br>(0.125)
Previous decision (bet)	1.499***<br>(0.098)	1.346***<br>(0.106)
Wealth	0.066<br>(0.066)	0.041<br>(0.071)
Last 5 outcomes	0.178***<br>(0.049)	0.200***<br>(0.053)
Session number	-0.245***<br>(0.051)	-0.257***<br>(0.055)
Black swan distance (>8m)	0.176<br>(0.144)	
Conditional *R2*	0.27	0.26
N	2969	2397

Table S9. Logistic mixed effects model predicting penny picking. The dependent variable is a dummy for betting after a black swan event (0: Skip; 1: Bet), for all five experiments. The independent variables are dummy coded variables for experiment, to compare Experiment 1 (reference) to Experiments 2-5, "First bet" (0: penny picking starts in the first 2 trials after black swan occurrence; 1: it starts in the third trial or after), the nature of the previous decision (0: Skip, 1: Bet), the current net accumulated outcomes ("wealth"), the mean outcome from the previous 5 trials ("last 5 outcomes"; the results are qualitatively unchanged when using the previous outcome, last 3 outcomes, and last 10 outcomes instead), session number, and for the 7 meters version of the model, the distance of the black swan from target (0: between 7 and 8 meters; 1: beyond 8 meters). The criteria used to measure penny picking are the same as in Table S8. As the split for the 8 meters and 9 meters criteria is identical, the results are combined into one column here. Wealth, mean outcome for last 5 trials, and session number were z-scored to standardize betas. By-participant intercepts were used to control for subject-wise error rates. Due to small group sizes for some participants, fixed effects were initialized with Cauchy priors (*86*). We assessed conditional R2 following Nakagawa and Schielzeth's method (*87*). * < .10, ** < .05, *** < .01.

	Base model	PP x Previous decision	PP x Wealth	PP x Last 5 outcomes	PP x Session Number
Intercept	-1.524***	-1.749***	-1.750 ***	-1.729 ***	-1.734 ***
	(.047)	(.048)	(.048)	(.048)	(.048)
Experiment 2	0.130	0.117	0.109	0.111	0.093
	(.082)	(.082)	(.081)	(.082)	(.083)
Experiment 3	0.037	0.065	0.065	0.053	0.056
	(.075)	(.074)	(.074)	(.075)	(.075)
Experiment 4	-0.075	-0.071	-0.074	-0.078	-0.077
	(.072)	(.072)	(.071)	(.072)	(.072
Experiment 5	-0.180 **	-0.167 **	-0.168 **	-0.173 **	-0.169 **
	(.078)	(.078)	(.077)	(.078)	(.078)
Bowman (apprentice)	-1.860 ***	-1.823 ***	-1.824 ***	-1.817 ***	-1.820 ***
	(.024)	(.024)	(.024)	(.024)	(.024)
Penny picking (PP)	0.804 ***	1.340 ***	1.333 ***	1.311***	1.318 ***
	(.057)	(.059)	(.059)	(.060	(.060)
Previous decision (bet)	3.496 ***	3.958 ***	3.956 ***	3.895***	3.892 ***
	(.022)	(.029)	(.029)	(.030	(.030)
Wealth	0.078 ***	0.077 ***	0.097 ***	0.053 **	0.091 ***
	(.018)	(.017)	(.027)	(.027)	(.031)
Last 5 outcomes	0.148 ***	0.120 ***	0.120 ***	0.302 ***	0.300 ***
	(.010)	(.010)	(.010)	(.018)	(.018)
Session number	-0.181 ***	-0.177 ***	-0.181 ***	-0.175 ***	-0.211 ***
	(.012)	(.012)	(.013)	(.013)	(.020)
PP x Previous decision	-	-1.169 ***	-1.166 ***	-1.112 ***	-1.109 ***
		(.044)	(.044)	(.044)	(.044)
PP x Wealth	-	-	-0.035	-0.033	-0.005
			(.034)	(.035)	(.038)
PP x Last 5 outcomes	-	-	-	-0.254 ***	-0.254 ***
				(.021)	(.021)
PP x Session number	-	-	-	-	0.062 **
					(.025)
AIC	60498.50	59810.59	59811.57	59663.86	59660.10
χ^2	-	689.91, p < .001	1.0212, p = .3122	149.7, p < .001	5.7609, p = .01639
Conditional R^2	.64	.65	.65	.65	.65
N	99313	99313	99313	99313	99313

Table S10. Logistic mixed effects model predicting betting for all participants in all trials. The dependent variable is a dummy for betting decision (0: Skip; 1: Bet). The independent variables include dummy coded variables for experiment [dummy to compare Experiment 1 (the reference) to Experiments 2-5], bowman type (0: Master; 1: Apprentice), participant type (0: Non penny-picker, 1: Penny-picker), the previous decision, session number, ``wealth" and ``last 5 outcomes" (see Table S9 for the definitions),

and interaction terms between penny picking and previous decision, wealth, last 5 outcomes, and session number. By-participant intercepts are used to control for subject-wise error rates. Due to small group sizes for some participants, fixed effects were initialized with Cauchy priors (*86*). The stepwise process of adding interaction terms involved adding an interaction term and comparing AIC for the previous model and the augmented model as well as running a chi-squared test to see whether the augmented model has better fit. All models use the strict criterion for penny picking as defined in the main text. We assessed conditional R2 following Nakagawa and Schielzeth's method (*87*).

	Panel A: MID Returns			Panel B: SELL Returns		
	(1)	(2)	(3)	(1)	(2)	(3)
MP	-0.006**	-0.008***	-0.008***	0.006**	0.008***	0.008***
	(2.51)	(2.91)	(2.93)	(2.51)	(2.94)	(2.96)
SIZE	-0.002***	-0.002***	-0.002***	0.002***	0.002***	0.002***
	(3.10)	(2.85)	(2.77)	(3.14)	(2.86)	(2.78)
1YR	-0.033	-0.031	-0.029	0.034	0.031	0.029
	(1.14)	(1.08)	(1.02)	(1.16)	(1.09)	(1.02)
BM	0.146***	0.131***	0.123***	-0.152***	-0.135***	-0.127***
	(2.90)	(2.81)	(2.76)	(2.90)	(2.80)	(2.75)
Δ	0.113*	0.45***	0.433***	0.017	-0.366***	-0.349***
	(1.83)	(6.62)	(6.21)	(0.27)	(5.33)	(4.96)
IV	-0.067	-0.068	-0.108	0.066	0.068	0.11
	(0.81)	(0.84)	(1.08)	(0.78)	(0.82)	(1.09)
Mat	0.085***	0.11***	0.105***	-0.077***	-0.105***	-0.1***
	(8.36)	(10.32)	(10.04)	(7.48)	(9.79)	(9.48)
SP	-0.324***	-0.322***	-0.308***	0.304***	0.303***	0.287***
	(12.28)	(11.56)	(11.11)	(11.64)	(10.88)	(10.40)
$1_{\text{Low Yield}}$			0.074***			-0.076***
			(8.88)			(9.05)
$1_{\text{Low VIX}}$			-0.02***			0.021***
			(7.76)			(7.92)
MP<1		0.333***	0.325***		-0.378***	-0.37***
		(13.53)	(13.89)		(15.11)	(15.54)
α	0.094*	-0.166***	-0.148***	-0.248***	0.048	0.03
	(1.66)	(3.25)	(2.76)	(4.31)	(0.92)	(0.55)
Firms			5830			5830
Observations			31,288,565			31,288,565

Table S11. Fixed effects call returns. Regressions corresponding to Eq. (11) in article, without (regression (1)) and with (regression (2)) the dummy MP < 1 included. The model underlying regression (3) is Eq. (11) augmented with dummy variables $1_{\text{Low Yield}}$ and $1_{\text{Low VIX}}$ to control for time variation in market conditions. $1_{\text{Low Yield}}$ (resp. $1_{\text{Low VIX}}$) is 1 in periods when the Bloomberg/Barclays US Corporate High Yield Index minus the yield on US Treasury bonds (resp. the *VIX* index) is below its long-term average, and 0 otherwise. The dependent variable is the MID returns (Panel A) and the SELL returns (Panel B) of the call options. Clustered and heteroskedasticity robust t-statistics are in parentheses. * < 0.10, ** < 0.05, *** < 0.01.

		All Options			Puts < $1			Puts ≥ $1		
		MID	BUY	SELL	MID	BUY	SELL	MID	BUY	SELL
Average	%	-0.230	-0.280	0.175	0.276	0.162	-0.408	0.173	0.128	-0.221
	$	-0.471	-0.605	0.338	0.155	0.107	-0.203	0.763	0.569	-0.957
Median	%	-0.944	-1.000	0.889	-0.923	-1.000	0.857	0.111	0.076	-0.149
	$	-0.575	-0.650	0.500	-0.175	-0.200	0.150	0.625	0.450	-0.800
SD	%	1.911	1.782	2.063	2.457	2.279	2.670	0.879	0.846	0.918
	$	3.682	3.657	3.714	1.044	1.023	1.067	3.946	3.921	3.978
Skew	%	14.051	13.413	-14.811	5.525	5.302	-5.801	1.144	1.095	-1.214
	$	1.299	1.143	-1.446	2.157	2.153	-2.159	2.153	-0.178	0.089
OTM (in %)		0.493			0.849			0.440		
Unique N		49,503			9,532			24,555		
N		1,256,229			124,455			386,765		

Table S12. "Cheap put selling anomaly" for options defined on negative beta assets. Summary statistics of MID, BUY, and SELL returns for all options, cheap puts (puts <$1), and the other puts (puts ≥ $1), defined on negative beta assets such as VIX. Both percent returns and dollar returns are shown (to highlight that the percent returns are not driven by small outliers). The table also reports median returns (Median), standard deviation (SD), the proportion of options that expire out-of-the-money (OTM), which is when the put sellers win money, the number of unique options in the sample (Unique N), and the total number of options in the sample (N). As predicted by CbD hypothesis, selling puts on negative beta assets results in EV<0, a median return that is positive, and SKEW<0, even after accounting for bid-ask spreads. Selling puts above $1 also has an EV<0 but the effect is significantly smaller than that with the cheap puts, the median return is negative, and SKEW is not different from zero.

	Firm Open Sell	Firm Open Buy	Customer Open Sell	Customer Open buy
Calls < $1	275.80	229.69	73.88	109.61
All Other Options	218.44	207.31	60.93	81.84
Difference	57.357***	22.383***	12.950***	27.769***
	(15.97)	(7.51)	(27.81)	(41.45)
Observations	250998	276101	1759959	1846315

Table S13. Average volume of open buy and open sell transactions, separated by trader class. Volume is measured by the number of new positions initiated. "Difference": return difference between cheap calls and all other options. Robust t-statistics are in parentheses. Significance levels: * p < 0:10, ** p < 0:05, and *** p < 0:01.

	Firms		Customers	
	Open Sell	Open Buy	Open Sell	Open Buy
MP	-1.73**	-1.84***	-0.937***	-1.286***
	(1.97)	(3.26)	(3.89)	(4.98)
SIZE	-0.001***	-0.001***	-0.001***	-0.001***
	(6.81)	(4.50)	(2.96)	(4.65)
1YR	-0.043	0.035	0.003	-0.006
	(1.20)	(1.00)	(0.48)	(0.54)
BM	39.302*	14.311	-1.095	-0.971
	(1.86)	(1.29)	(1.13)	(0.41)
Δ	-14.69***	-0.999	3.722***	-1.922***
	(3.69)	(0.32)	(6.37)	-(1.00)
IV	76.496***	85.229***	52.091***	85.925***
	(6.77)	(7.32)	(9.28)	(12.32)
Mat	0.455***	0.221***	-0.097***	-0.179***
	(7.29)	(4.75)	(6.19)	(6.87)
SP	-43.926***	-12.896*	-12.077***	-19.6**
	(3.34)	(1.82)	(2.19)	(2.26)
$1_{Low\ Yield}$	24.265***	59.17***	16.091***	14.939***
	(3.07)	(7.77)	(6.90)	(4.39)
$1_{Low\ VIX}$	9.188	6.653	0.055	7.01***
	(0.97)	(1.13)	(0.03)	(2.73)
MP<1	54.879***	13.436	11.423***	34.108***
	(7.75)	(1.62)	(5.86)	(9.68)
α	118.074***	114.386***	44.139***	55.318***
	(7.00)	(8.08)	(9.40)	(9.21)
Firms	3,101	3,162	3,308	3,315
Observations	600,574	703,266	4,257,236	3,744,950

Table S14. Firm fixed effects regression on call volume by trader class. The dependent variable is either the volume of an open buy or an open sell transaction for firms (left) and customers (right) as defined by the *International Securities Exchange*. The independent variables are the same as those used in Table S11, regression (3). Clustered and heteroskedasticity robust t-statistics are in parentheses. * < 0.10, ** < 0.05, *** < 0.01.

		(1)		(2)		(3)		(4)		(5)	
		**Power**									
		0.80	0.90	0.80	0.90	0.80	0.90	0.80	0.90	0.80	0.90
Alpha	0.05	0.18	0.21	0.59	0.69	0.25	0.29	0.25	0.29	0.25	0.29
	0.1	0.16	0.19	0.53	0.62	0.22	0.26	0.22	0.26	0.22	0.26

Table S15. Minimum effect sizes needed to find a significant effect given our sample size (N) for each test, for different levels of significance (alpha) and power. (1) χ^2 test comparing the proportion of penny-pickers in the "no ambiguity" vs. learning versions of the task. Effect size is measured as *Cramer's V*, N=168. **(2)** Two-sample t-test comparing the accuracy of the penny-pickers vs. the other participants, using the data from Experiments 3 and 5 (N =99). Accuracy is defined as the fraction of correct replies as explained in the article (*note*: accuracy was only measured in Experiments 3 and 5). Effect size is measured as *Cohen's D*. **(3)** χ^2 test comparing the proportion of penny-pickers in Experiment 4 vs. Experiments 1 and 3 (N=184). Effect size is measured as *Cramer's V*. **(4)** χ^2 test comparing the proportion of penny-pickers in Experiment 5 vs. Experiments 1 and 3. Effect size is measured as *Cramer's V*, N=223. **(5)** Paired t-test comparing the goodness of fit of the CbD vs. base models. Effect size is measured as *Cohen's D*, N=120.

Panel A: IV Groups

	SELL			MID			SELL SKEW	SELL SKEW
	Call $<1	Call $≥1	Diff	Call $<1	Call $≥1	Diff	for calls <$1	for calls ≥$1
IV<.2	-0.404***	-0.192***	-0.212***	0.258***	0.116	0.142**	-9.97	-3.85
	(5.38)	(4.58)	(2.74)	(1.95)	(1.22)	(2.19)		
.2<IV<.35	-0.238***	-0.102***	-0.136***	0.084***	0.027	0.058**	-7.94	-6.29
	(5.38)	(4.58)	(2.74)	(1.95)	(1.22)	(2.19)		
.35<IV<.5	-0.202***	-0.075***	-0.126***	0.049	0	0.048**	-12.47	-6.87
	(4.60)	(3.41)	(2.57)	(1.13)	(0.01)	(2.00)		
.5<IV<.65	-0.123***	-0.038***	-0.085***	-0.029	-0.039***	0.01	-9.01	-6.09
	(3.05)	(1.74)	(1.86)	(0.73)	(1.83)	(0.22)		
.65<IV	-0.017	0.027	-0.044	-0.137***	-0.1***	-0.037	-13.90	-15.37
	(0.45)	(1.53)	(1.07)	(3.75)	(5.71)	(0.91)		
H-L	0.387***	0.219***	0.168***	-0.395***	-0.216***	-0.179***		
	(6.29)	(7.58)	(3.22)	(6.56)	(7.60)	(3.50)		

Panel B: Delta Groups

	SELL			MID			SELL SKEW	SELL SKEW
	Call $<1	Call $≥1	Diff	Call $<1	Call $<1	Call $≥1	Diff	Call $<1
Δ<.1	-0.193***	0.079	-0.272***	0.133	-0.169***	0.202***	-63.41	-20.88
	(3.07)	(1.64)	(3.43)	(0.53)	(3.52)	(2.58)		
.1<Δ<.2	-0.264***	-0.074**	-0.19***	0.116***	-0.019***	0.134***	-21.99	-10.90
	(6.94)	(2.07)	(3.63)	(3.11)	(0.52)	(2.62)		
.2<Δ<.3	-0.264***	-0.111***	-0.154***	0.116***	0.018	0.098**	-12.90	-6.61
	(8.47)	(3.73)	(3.56)	(3.82)	(0.62)	(2.33)		
.3<Δ<.4	-0.25***	-0.114***	-0.136***	0.102***	0.024	0.077**	-11.81	-4.67
	(9.19)	(4.46)	(3.64)	(3.85)	(0.97)	(2.12)		
.4<Δ<.5	-0.228***	-0.102***	-0.126***	0.08***	0.017	0.063**	-9.51	-3.45
	(9.52)	(4.52)	(3.84)	(3.45)	(0.76)	(1.98)		
.5<Δ<.6	-0.217***	-0.082***	-0.134***	0.07***	0.003	0.067**	-10.27	-3.27
	(10.06)	(4.14)	(4.58)	(3.33)	(0.15)	(2.33)		
.6<Δ<.7	-0.194***	-0.069***	-0.125***	0.05***	-0.005	0.055**	-9.07	-3.13
	(10.28)	(3.93)	(4.87)	(2.72)	(0.30)	(2.19)		
.7<Δ<.8	-0.17***	-0.062***	-0.108***	0.029*	-0.007	0.036*	-6.53	-6.71
	(10.68)	(4.22)	(4.95)	(1.90)	(0.46)	(1.69)		
.8<Δ<.9	-0.15***	-0.057***	-0.092***	0.013	-0.007	0.021	-6.21	-5.41
	(12.11)	(4.91)	(5.44)	(1.12)	(0.63)	(1.25)		
.9<Δ<1	-0.143***	-0.053***	-0.09***	0.016*	0.002	0.014	-9.29	-5.15
	(14.65)	(6.95)	(7.26)	(1.72)	(0.24)	(1.19)		
H (10 & 9)-L (1 &2)	0.082*	-0.058***	0.14***	-0.159	0.091**	-0.251***		
	(1.79)	(1.46)	(3.08)	(1.33)	(2.33)	(3.38)		

Table S16. Difference in mean option returns sorted into implied volatility quintiles and delta deciles. "Diff" denotes the difference in mean returns between the cheap calls and the calls priced above one dollar. Returns are sorted into implied volatility (IV) quintiles (Panel A) and delta (Δ) deciles (Panel B). Robust t-statistics are given in parentheses. * < 0.10, ** < 0.05, *** < 0.01.

	MID Returns		SELL Returns	
	Calls < $1	Calls ≥ $1	Calls < $1	Calls ≥ $1
MP	-1.281***	-0.007***	1.485***	0.007***
	(10.88)	(3.04)	(12.45)	(3.08)
SIZE	-0.005***	-0.002***	0.005*	0.002***
	(1.86)	(3.12)	(1.85)	(3.14)
1YR	-0.071	-0.016	0.075	0.016
	(1.01)	(0.65)	(1.00)	(0.66)
BM	0.014	0.374***	-0.023	-0.387***
	(1.08)	(7.09)	(1.14)	(7.14)
D	3.453***	0.323***	-3.48***	-0.243***
	(15.86)	(5.22)	(15.81)	(3.89)
IV	-0.336	-0.14	0.352	0.151*
	(1.57)	(1.61)	(1.62)	(1.72)
Mat	0.316***	0.083***	-0.316***	-0.078***
	(9.78)	(9.02)	(9.70)	(8.38)
SP	-0.365	-0.296***	-0.458	0.276***
	(0.94)	(15.49)	(1.16)	(14.14)
$1_{Low\ Yield}$	0.126***	0.053***	-0.129***	-0.055***
	(8.26)	(6.71)	(8.36)	(6.88)
$1_{Low\ VIX}$	-0.039***	-0.015***	0.04***	0.015***
	(8.58)	(5.92)	(8.74)	(6.04)
α	0.005	-0.135***	-0.201	0.02
	(0.03)	(3.09)	(1.05)	(0.45)
Firms	5,432	5,765	5,432	5,765
Observations	5,921,492	25,360,438	5,921,492	25,360,438

Table S17. Firm fixed effects call returns, by call category (cheap calls vs. calls priced above $1). The underlying model is the same as the one in Table S11, regression (3), but excluding the MP<1 dummy variable, and by call category. See Table S11 for the legend.

Panel A: MID Returns

	All Options				Calls				Calls >=$1	Calls<$1
	1	2	3	4	5	6	7	8	9	10
MP	-0.001	-0.0004	-0.0003	-0.0004	-0.003**	-0.005***	-0.004***	-0.004***	-0.004***	-1.147***
	(0.97)	(0.59)	(0.52)	(0.55)	(2.06)	(2.83)	(2.70)	(2.77)	(2.90)	(5.44)
SIZE	-0.001***	-0.0009***	-0.0009***	-0.0009***	-0.002*	-0.002*	-0.001	-0.001	-0.001	-0.006**
	(2.81)	(2.92)	(2.75)	(2.74)	(1.77)	(1.80)	(1.65)	(1.55)	(0.93)	(2.16)
1YR	-0.019	-0.0167	-0.0141	-0.0145	-0.0331	-0.0312	-0.0261	-0.0232	-0.0111	-0.0678
	(1.28)	(1.19)	(1.07)	(1.10)	(1.14)	(1.09)	(0.94)	(0.85)	(0.46)	(0.98)
BM	0.006	0.0031	-0.0019	-0.0026	0.096*	0.089*	0.071**	0.065**	0.254***	0.016
	(0.97)	(0.57)	(0.33)	(0.46)	(1.92)	(1.90)	(1.99)	(1.95)	(5.58)	(1.20)
Δ	0.233***	0.215***	0.222***	0.217***	0.043	0.32***	0.225***	0.214***	0.241***	2.905***
	(12.52)	(12.20)	(12.56)	(12.25)	(0.81)	(6.59)	(5.14)	(4.55)	(5.17)	(7.15)
ν	0.118***	0.115**	0.111**	0.084*	0.088	0.168***	0.132**	0.116**	0.069	10.086***
	(2.75)	(2.53)	(2.33)	(1.87)	(1.50)	(2.83)	(2.26)	(1.96)	(1.32)	(4.94)
τ	0.882***	0.844***	0.841***	0.765***	1.666***	1.624***	1.633***	1.56***	1.492***	20.239***
	(2.87)	(2.86)	(2.91)	(2.79)	(2.84)	(2.84)	(2.91)	(2.88)	(3.29)	(9.12)
SP	-0.117***	-0.097***	-0.074***	-0.06***	-0.259***	-0.264***	-0.2***	-0.19***	-0.166***	-0.196
	(6.56)	(5.95)	(4.78)	(3.97)	(6.51)	(6.77)	(5.66)	(5.46)	(6.36)	(0.50)
$1_{LOW\ YIELD}$				0.041***				0.069***	0.047***	0.127***
				(9.24)				(8.19)	(6.07)	(7.80)
$1_{Low\ VIX}$				-0.013***				-0.018***	-0.012***	-0.033***
				(11.84)				(7.34)	(5.28)	(8.00)
MP<1		0.255***		0.214***		0.266***		0.141***		
		(8.02)		(6.69)		(10.02)		(6.12)		
γ			1.72***	1.651***			3.65***	3.579***	5.754***	0.694
			(10.38)	(9.84)			(12.08)	(11.86)	(14.55)	(1.17)
α	0.039	0.006	-0.082***	-0.052	0.285***	0.097	-0.038	-0.06	-0.232***	0.21
	(1.35)	(0.23)	(2.77)	(1.52)	(3.93)	(1.32)	(0.51)	(0.68)	(2.62)	(0.88)
Firms				5,879				5,828	5,762	5,432
Observations				55,926,006				31,272,660	25,351,162	5,921,498

Panel B: SELL
Returns

	All Options				Calls				Calls >=$1	Calls<$1
	1	2	3	4	5	6	7	8	9	10
MP	0.003**	0.005***	0.004***	0.004***	0.003**	0.005***	0.004***	0.004***	0.004***	1.348***
	(1.98)	(2.82)	(2.71)	(2.79)	(1.98)	(2.82)	(2.71)	(2.79)	(2.98)	(6.30)
SIZE	0.002*	0.002*	0.001	0.001	0.002*	0.002*	0.001	0.001	0.001	0.006**
	(1.68)	(1.70)	(1.55)	(1.45)	(1.68)	(1.70)	(1.55)	(1.45)	(0.82)	(2.15)
1YR	0.034	0.0318	0.0265	0.0235	0.034	0.0318	0.0265	0.0235	0.011	0.0676
	(1.16)	(1.10)	(0.94)	(0.85)	(1.16)	(1.10)	(0.94)	(0.85)	(0.47)	(0.97)
BM	-0.1*	-0.091*	-0.073**	-0.067*	-0.1*	-0.091*	-0.073**	-0.067*	-0.26***	-0.015
	(1.91)	(1.89)	(1.98)	(1.94)	(1.91)	(1.89)	(1.98)	(1.94)	(5.61)	(1.17)
Δ	0.093*	-0.229***	-0.129***	-0.118***	0.093*	-0.229***	-0.129***	-0.118***	-0.1583***	-2.924***
	(1.78)	(4.75)	(2.98)	(2.53)	(1.78)	(4.75)	(2.98)	(2.53)	(3.39)	(7.04)
ν	-0.048	-0.141**	-0.103*	-0.086	-0.048	-0.141**	-0.103*	-0.086	-0.044	-10.197***
	(0.76)	(2.31)	(1.72)	(1.42)	(0.76)	(2.31)	(1.72)	(1.42)	(0.82)	(4.95)
τ	-1.719***	-1.67***	-1.679***	-1.605***	-1.719***	-1.67***	-1.679***	-1.605***	-1.533***	-20.621***
	(2.84)	(2.84)	(2.91)	(2.88)	(2.84)	(2.84)	(2.91)	(2.88)	(3.29)	(9.17)
SP	0.234***	0.241***	0.173***	0.163***	0.234***	0.241***	0.173***	0.163***	0.138***	-0.616
	(5.74)	(6.03)	(4.79)	(4.59)	(5.74)	(6.03)	(4.79)	(4.59)	(5.15)	(1.54)
$1_{LOW\ YIELD}$				-0.071***				-0.071***	-0.049***	-0.13***
				(8.32)				(8.32)	(6.19)	(7.87)
$1_{Low\ VIX}$				0.019***				0.019***	0.012***	0.034***
				(7.48)				(7.48)	(5.37)	(8.17)
MP<1		-0.309***		-0.178***		-0.309***		-0.178***		
		(11.37)		(7.55)		(11.37)		(7.55)		
γ			-3.837***	-3.765***			-3.837***	-3.765***	-6.145***	-0.729
			(12.30)	(12.08)			(12.30)	(12.08)	(15.18)	(1.20)
α	-0.433***	-0.214***	-0.072	-0.049	-0.433***	-0.214***	-0.072	-0.049	0.141	-0.403
	(5.76)	(2.85)	(0.93)	(0.54)	(5.76)	(2.85)	(0.93)	(0.54)	(1.55)	(1.66)
Firms			5,828				5,828		5,762	5,432
Observations			31,272,660				31,272,660		25,351,162	5,921,498

Table S18. Firm fixed effects regressions on option returns including "Greeks" option specific characteristics. The underlying model is the same as the one in Table S11, regression (3), augmented with the option specific characteristics vega, theta, and gamma. See Table S11 for the legend. The results fully replicate those documented in the main text. For the MID specification on all options, when {MP<1} is not included (specifications 1,3,5 and 7), we observe a negative coefficient on MP, a positive coefficient on delta, a negative coefficient on vega, a positive coefficient on theta, a negative coefficient on the bid-ask spread variable, and a positive coefficient on gamma. This confirms that buying higher priced options and options with wider bid-ask spreads yield lower returns, lower delta options have lower returns, and options with higher volatility have lower returns. The coefficient on {MP<1} is positive and significant in the MID regressions when the variable is included (specifications 2, 4, 6, and 8), showing that the cheap calls have a different return profile relative to the other options. Also note how, in the SELL regressions, the coefficient on {MP<1} is negative when the variable is included, and how that variable helps bring the constant term close to zero and insignificant. The results show that cheap call selling results in 17% worse returns when gamma is included. Separating the sample into calls above $1 and calls below $1 (specifications 9,10) further replicate the results documented in Table S17. This shows that the evidence for the cheap call selling anomaly presents itself consistently even after fully controlling for key aspects such as the volatility premium, the gambling premium, and unhedgeable risk.

Panel A: Open Sell Transaction

	Customers		Firms	
	MID Returns	SELL Returns	MID Returns	SELL Returns
Calls < $1	0.055	-0.250	0.055	-0.342
# observations	741,676	741,676	93,597	93,597
All Other Options	-0.124	0.037	-0.127	-0.080
# observations	2,004,484	2,004,484	298,295	298,295
Difference	0.179***	-0.286***	0.181***	-0.262**
	(2.68)	(3.07)	(2.76)	(2.14)

Panel B: Open Buy Transaction

	Customers		Firms	
	MID Returns	SELL Returns	MID Returns	SELL Returns
Calls < $1	-0.067	-0.276	-0.023	-0.274
# observations	580,764	580,764	115,974	115,974
All Other Options	-0.140	-0.240	-0.104	-0.242
# observations	1,800,263	1,800,263	329,084	329,084
Difference	0.0728	-0.0357	0.0805	-0.032
	(0.76)	(0.20)	(1.38)	(0.56)

Table S19. The cheap call selling anomaly replicated in transaction-level data. This table reports the MID and SELL returns for open buy and open sell transactions of cheap calls vs. all other options, separated by trader class. "Difference": return difference between cheap calls and all other options. Robust t-statistics are in parentheses. Significance levels: * p < 0:10, ** p < 0:05, and *** p < 0:01. The table shows that the MID returns (resp. SELL returns) for open sell transactions are positive (resp. negative) for the cheap calls vs. negative (resp. positive) for all other options, consistent with the CbD hypothesis proposed in the article. For open buy transactions, both MID and SELL returns are negative, consistent with the gambling motive evidenced in prior work (see references in main text).

		Percentage of penny-pickers	Penny picking frequency				
			Pick pennies in at least (%):			Mean	SD
			2 sessions	3 sessions	4 sessions		
All black swan events	7m	63.6	85.7	67.3	57.1	2.90	2.97
	8m	62.3	85.4	66.7	58.3	2.79	2.92
	9m	62.3	85.4	62.5	52.1	2.66	2.84
Only memorable events	7m	53.2	82.9	56.1	46.3	1.91	2.29
	8m	53.2	82.9	56.1	39.0	1.82	2.20
	9m	51.9	77.5	52.5	40.0	1.70	2.09

Table S20. Replication of the main experimental findings in an independent sample at Brown University (N = 77).

REFERENCES AND NOTES

1. R. A. Rescorla, R. L. Solomon, Two-process learning theory: Relationships between Pavlovian conditioning and instrumental learning. *Psychol. Rev.* **74**, 151–182 (1967).

2. M. Guitart-Masip, E. Duzel, R. Dolan, P. Dayan, Action versus valence in decision making. *Trends Cogn. Sci.* **18**, 194–202 (2014).

3. K. Breland, M. Breland, The misbehavior of organisms. *Am. Psychol.* **16**, 681–684 (1961).

4. P. Dayan, B. W. Balleine, Reward, motivation, and reinforcement learning. *Neuron* **36**, 285–298 (2002).

5. P. Dayan, Y. Niv, B. Seymour, N. D. Daw, The misbehavior of value and the discipline of the will. *Neural Netw.* **19**, 1153–1160 (2006).

6. P. C. Holland, Differential effects of omission contingencies on various components of Pavlovian appetitive conditioned responding in rats. *J. Exp. Psychol. Anim. Behav. Process.* **5**, 178–193 (1979).

7. W. A. Hershberger, An approach through the looking-glass. *Anim. Learn. Behav.* **14**, 443–451 (1986).

8. M. Guitart-Masip, L. Fuentemilla, D. R. Bach, Q. J. M. Huys, P. Dayan, R. J. Dolan, E. Duzel, Action dominates valence in anticipatory representations in the human striatum and dopaminergic midbrain. *J. Neurosci.* **31**, 7867–7875 (2011).

9. J. F. Cavanagh, I. Eisenberg, M. Guitart-Masip, Q. Huys, M. J. Frank, Frontal theta overrides Pavlovian learning biases. *J. Neurosci.* **33**, 8541–8548 (2013).

10. S. J. Gershman, M. Guitart-Masip, J. F. Cavanagh, Neural signatures of arbitration between Pavlovian and instrumental action selection. *PLOS Comput. Biol.* **17**, e1008553 (2021).

11. A. D. Redish, *The Mind Within the Brain: How We Make Decisions and How Those Decisions Go Wrong* (Oxford Univ. Press, 2013).

12. P. Winkielman, K. Berridge, Irrational wanting and subrational liking: How rudimentary motivational and affective processes shape preferences and choices. *Polit. Psychol.* **24**, 657–680 (2003).

13. R. B. Rutledge, N. Skandali, P. Dayan, R. J. Dolan, Dopaminergic modulation of decision making and subjective well-being. *J. Neurosci.* **35**, 9811–9822 (2015).

14. R. B. Rutledge, P. Smittenaar, P. Zeidman, H. R. Brown, R. A. Adams, U. Lindenberger, P. Dayan, R. J. Dolan, Risk taking for potential reward decreases across the lifespan. *Curr. Biol.* **26**, 1634–1639 (2016).

15. N. D. Schüll, *Addiction by Design: Machine Gambling in Las Vegas* (Princeton Univ. Press, 2014).

16. J. Conlisk, The utility of gambling. *Insur. Math. Econ.* **13**, 166–167 (1993).

17. M. Toce-Gerstein, D. R. Gerstein, R. A. Volberg, A hierarchy of gambling disorders in the community. *Addiction* **98**, 1661–1672 (2003).

18. G. Zuckerman, *The Greatest Trade Ever: The Behind-the-Scenes Story of How John Paulson Defied Wall Street and Made Financial History* (National Geographic Books, 2010).

19. N. N. Taleb, *The Black Swan: The Impact of the Highly Improbable* (Penguin UK, 2008).

20. R. J. Shiller, *Irrational Exuberance: Revised and Expanded Third Edition* (Princeton Univ. Press, 2016).

21. A. W. Lo, *Adaptive Markets: Financial Evolution at the Speed of Thought* (Princeton Univ. Press, 2019).

22. D. Kahneman, A. Tversky, Prospect theory: An analysis of decision under risk. *Econometrica* **47**, 263–292 (1979).

23. A. L. Brown, T. Imai, F. Vieider, C. Camerer, *Meta-Analysis of Empirical Estimates of Loss Aversion* (Center for Open Science, 2020).

24. M. Hsu, M. Bhatt, R. Adolphs, D. Tranel, C. F. Camerer, Neural systems responding to degrees of uncertainty in human decision-making. *Science* **310**, 1680–1683 (2005).

25. A. Tversky, D. Kahneman, Advances in prospect theory: Cumulative representation of uncertainty. *J. Risk Uncertainty* **5**, 297–323 (1992).

26. P. P. Wakker, *Prospect Theory: For Risk and Ambiguit*y (Cambridge Univ. Press, 2012).

27. D. Prelec, The probability weighting function. *Econometrica* **66**, 497–527 (1998).

28. T. Hens, M. Vlcek, Does prospect theory explain the disposition effect? *J. Behav. Financ.* **12**, 141–157 (2011).

29. Y. Li, L. Yang, Prospect theory, the disposition effect, and asset prices. *J. Financ. Econ.* **107**, 715–739 (2013).

30. S. M. McClure, N. D. Daw, P. Read Montague, A computational substrate for incentive salience. *Trends Neurosci.* **26**, 423–428 (2003).

31. J. Zhang, K. C. Berridge, A. J. Tindell, K. S. Smith, J. W. Aldridge, A neural computational model of incentive salience. *PLOS Comput. Biol.* **5**, e1000437 (2009).

32. A. Olsson, E. A. Phelps, Learned fear of "unseen" faces after pavlovian, observational, and instructed fear. *Psychol. Sci.* **15**, 822–828 (2004).

33. C. D. Fiorillo, P. N. Tobler, W. Schultz, Discrete coding of reward probability and uncertainty by dopamine neurons. *Science* **299**, 1898–1902 (2003).

34. P. N. Tobler, C. D. Fiorillo, W. Schultz, Adaptive coding of reward value by dopamine neurons. *Science* **307**, 1642–1645 (2005).

35. M. Pessiglione, B. Seymour, G. Flandin, R. J. Dolan, C. D. Frith, Dopamine-dependent prediction errors underpin reward-seeking behaviour in humans. *Nature* **442**, 1042–1045 (2006).

36. K. Louie, Asymmetric and adaptive reward coding via normalized reinforcement learning. *PLOS Comput. Biol.* **18**, e1010350 (2022).

37. P. Glimcher, Understanding the hows and whys of decision-making: From expected utility to divisive normalization. *Cold Spring Harb. Symp. Quant. Biol.* **79**, 169–176 (2014).

38. C. L. Wyvell, K. C. Berridge, Incentive sensitization by previous amphetamine exposure: Increased cue-triggered "wanting" for sucrose reward. *J. Neurosci.* **21**, 7831–7840 (2001).

39. Y. Niv, N. D. Daw, D. Joel, P. Dayan, Tonic dopamine: Opportunity costs and the control of response vigor. *Psychopharmacology* **191**, 507–520 (2007).

40. P. Anselme, M. J. F. Robinson, Incentive motivation: The missing piece between learning and behavior, in *The Cambridge Handbook of Motivation and Learning* (Cambridge Univ. Press, 2019).

41. H. M. Dorfman, S. J. Gershman, *Controllability Governs the Balance Between Pavlovian and Instrumental Action Selection* (Cold Spring Harbor Laboratory, 2019).

42. P. Anselme, O. Güntürkün, How foraging works: Uncertainty magnifies food-seeking motivation. *Behav. Brain Sci.* **42**, e35 (2019).

43. R. Karlsson Linnér, P. Biroli, E. Kong, S. F. W. Meddens, R. Wedow, M. A. Fontana, M. Lebreton, S. P. Tino, A. Abdellaoui, A. R. Hammerschlag, M. G. Nivard, A. Okbay, C. A. Rietveld, P. N. Timshel, M. Trzaskowski, R. de Vlaming, C. L. Zünd, Y. Bao, L. Buzdugan, A. H. Caplin, C.-Y. Chen, P. Eibich, P. Fontanillas, J. R. Gonzalez, P. K. Joshi, V. Karhunen, A. Kleinman, R. Z. Levin, C. M. Lill, G. A. Meddens, G. Muntané, S. Sanchez-Roige, F. J. van Rooij, E. Taskesen, Y. Wu, F. Zhang, A. Auton, J. D. Boardman, D. W. Clark, A. Conlin, C. C. Dolan, U. Fischbacher, P. J. F. Groenen, K. M. Harris, G. Hasler, A. Hofman, M. A. Ikram, S. Jain, R. Karlsson, R. C. Kessler, M. Kooyman, J. MacKillop, M. Männikkö, C. Morcillo-Suarez, M. B. McQueen, K. M. Schmidt, M. C. Smart, M. Sutter, A. R. Thurik, A. G. Uitterlinden, J. White, H. de Wit, J. Yang, L. Bertram, D. I. Boomsma, T. Esko, E. Fehr, D. A. Hinds, M. Johannesson, M. Kumari, D. Laibson, P. K. E. Magnusson, M. N. Meyer, A. Navarro, A. A.

Palmer, T. H. Pers, D. Posthuma, D. Schunk, M. B. Stein, R. Svento, H. Tiemeier, P. R. H. J. Timmers, P. Turley, R. J. Ursano, G. G. Wagner, J. F. Wilson, J. Gratten, J. J. Lee, D. Cesarini, D. J. Benjamin, P. D. Koellinger, J. P. Beauchamp, Genome-wide association analyses of risk tolerance and risky behaviors in over 1 million individuals identify hundreds of loci and shared genetic influences. *Nat. Genet.* **51**, 245–257 (2019).

44. R. H. Thaler, E. J. Johnson, Gambling with the house money and trying to break even: The effects of prior outcomes on risky choice. *Manage. Sci.* **36**, 643–660 (1990).

45. B. Köszegi, M. Rabin, A model of reference-dependent preferences. *Q. J. Econ.* **121**, 1133–1165 (2006).

46. T. Post, M. J. van den Assem, G. Baltussen, R. H. Thaler, Deal or no deal? decision making under risk in a large-payoff game show. *Am. Econ. Rev.* **98**, 38–71 (2008).

47. N. Thakral, L. T. Tô, Daily labor supply and adaptive reference points. *Am. Econ. Rev.* **111**, 2417–2443 (2021).

48. J. Chapman, E. Snowberg, S. Wang, C. Camerer, *Looming Large or Seeming Small? Attitudes Towards Losses in a Representative Sample* (National Bureau of Economic Research, 2022).

49. C. D. Adams, Variations in the sensitivity of instrumental responding to reinforcer devaluation. *Q. J. Exp. Psychol. Sec. B* **34**, 77–98 (1982).

50. A. Dickinson, *Instrumental Conditioning in Animal Learning and Cognition* (Academic Press, 2013).

51. A. Dezfouli, B. W. Balleine, Actions, action sequences and habits: Evidence that goal-directed and habitual action control are hierarchically organized. *PLOS Comput. Biol.* **9**, e1003364 (2013).

52. N. D. Daw, Y. Niv, P. Dayan, Uncertainty-based competition between prefrontal and dorsolateral striatal systems for behavioral control. *Nat. Neurosci.* **8**, 1704–1711 (2005).

53. R. S. Sutton, A. G. Barto, *Reinforcement Learning, Second Edition: An Introduction* (MIT Press, 2018).

54. E. Payzan-LeNestour, Can people learn about 'black swans'? Experimental evidence. *Rev. Financ. Studies* **31**, 4815–4862 (2018).

55. J. Elster, *Reason and Rationality* (Princeton Univ. Press, 2008).

56. A. Alekseev, G. Charness, U. Gneezy, Experimental methods: When and why contextual instructions are important. *J. Econ. Behav. Org.* **134**, 48–59 (2017).

57. A. Lusardi, O. S. Mitchell, Baby boomer retirement security: The roles of planning, financial literacy, and housing wealth. *J. Monet. Econom.* **54**, 205–224 (2007).

58. E. Payzan-LeNestour, P. Bossaerts, Learning about unstable, publicly unobservable payoffs. *Rev. Financ. Studies* **28**, 1874–1913 (2015).

59. G. Charness, U. Gneezy, B. Halladay, Experimental methods: Pay one or pay all. *J. Econ. Behav. Organ.* **131**, 141–150 (2016).

60. G. Gigerenzer, U. Hoffrage, How to improve Bayesian reasoning without instruction: Frequency formats. *Psychol. Rev.* **102**, 684–704 (1995).

61. U. Hoffrage, S. Lindsey, R. Hertwig, G. Gigerenzer, Communicating statistical information. *Science* **290**, 2261–2262 (2000).

62. A. N. Sanborn, N. Chater, Bayesian brains without probabilities. *Trends Cogn. Sci.* **20**, 883–893 (2016).

63. O. Heffetz, Are reference points merely lagged beliefs over probabilities? *J. Econ. Behav. Organ.* **181**, 252–269 (2021).

64. J. Ameriks, A. Caplin, J. Leahy, T. Tyler, Measuring self-control problems. *Am. Econ. Rev.* **97**, 966–972 (2007).

65. A. Szollosi, B. R. Newell, People as intuitive scientists: Reconsidering statistical explanations of decision making. *Trends Cogn. Sci.* **24**, 1008–1018 (2020).

66. M. Guitart-Masip, Q. J. M. Huys, L. Fuentemilla, P. Dayan, E. Duzel, R. J. Dolan, Go and no-go learning in reward and punishment: Interactions between affect and effect. *Neuroimage* **62**, 154–166 (2012).

67. E. Cartoni, B. Balleine, G. Baldassarre, Appetitive Pavlovian-instrumental transfer: A review. *Neurosci. Biobehav. Rev.* **71**, 829–848 (2016).

68. R. D. McLean, J. Pontiff, Does academic research destroy stock return predictability? *J. Financ.* **71**, 5–32 (2016).

69. N. Feltovich, Reinforcement-based vs. belief-based learning models in experimental asymmetric-information games. *Econometrica* **68**, 605–641 (2000).

70. R. Hertwig, When to consider boosting: Some rules for policy-makers. *Behav. Public Policy* **1**, 143–161 (2017).

71. J. Pan, A. M. Poteshman, The information in option volume for future stock prices. *Rev. Financ. Stud.* **19**, 871–908 (2006).

72. D. Easley, M. O'Hara, P. S. Srinivas, Option volume and stock prices: Evidence on where informed traders trade. *J. Financ.* **53**, 431–465 (1998).

73. M. Cremers, M. Halling, D. Weinbaum, Aggregate jump and volatility risk in the cross-section of stock returns. *J. Financ.* **70**, 577–614 (2015).

74. P. Carr, L. Wu, Variance risk premiums. *Rev. Financ. Stud.* **22**, 1311–1341 (2009).

75. V. Bhansali, L. Harris, Everybody's doing it: Short volatility strategies and shadow financial insurers. *Financ. Anal. J.* **74**, 12–23 (2018).

76. K. J. M. Cremers, J. Driessen, P. Maenhout, Explaining the level of credit spreads: Option-implied jump risk premia in a firm value model. *Rev. Financ. Stud.* **21**, 2209–2242 (2008).

77. G. Bakshi, N. Kapadia, Delta-hedged gains and the negative market volatility risk premium. *Rev. Financ. Stud.* **16**, 527–566 (2003).

78. D. Muravyev, Order flow and expected option returns. *J. Financ.* **71**, 673–708 (2016).

79. M. A. Petersen, Estimating standard errors in finance panel data sets: Comparing approaches. *Rev. Financ. Stud.* **22**, 435–480 (2008).

80. A. B. Konova, K. Louie, P. W. Glimcher, The computational form of craving is a selective multiplication of economic value. *Proc. Natl. Acad. Sci. U.S.A.* **115**, 4122–4127 (2018).

81. R. Hertwig, G. M. Barron, E. U. Weber, I. Erev, Decisions from experience and the effect of rare events in risky choice. *SSRN Electron. J.* **15**, 534–539 (2008).

82. D. Prelec, D. Simester, Always leave home without it: A further investigation of the credit-card effect on willingness to pay *Marketing Lett.* **12**, 5–12 (2001).

83. B. Eraker, M. Ready, Do investors overpay for stocks with lottery-like payoffs? An examination of the returns of OTC stocks. *J. Financ. Econ.* **115**, 486–504 (2015).

84. W. Goetzmann, J. Ingersoll, M. Spiegel, I. Welch, *Sharpening Sharpe Ratios* (National Bureau of Economic Research, 2002).

85. D. Firth, Bias reduction of maximum likelihood estimates. *Biometrika* **80**, 27–38 (1993).

86. A. Gelman, A. Jakulin, M. G. Pittau, Y.-S. Su, A weakly informative default prior distribution for logistic and other regression models. *Ann. Appl. Stat.* **2**, 1360–1383 (2008).

87. S. Nakagawa, H. Schielzeth, A general and simple method for obtaining R^2 from generalized linear mixed-effects models. *Methods Ecol. Evol.* **4**, 133–142 (2013).
